# Prophylaxis Regimens for *Pneumocystis jirovecii* Pneumonia in Non-HIV, Non-Malignant Immunocompromised Adults: A Systematic Review and Network Meta-Analysis

**DOI:** 10.3390/jof12080627

**Published:** 2026-08-20

**Authors:** Xiaojing Wu, Yue Zhang, Keming Wang, Liuluan Zhu

**Affiliations:** Beijing Key Laboratory of Viral Infectious Diseases, Beijing Institute of Infectious Diseases, Beijing Ditan Hospital, Capital Medical University, Beijing 100015, China; wuxiaojing914@163.com (X.W.); yuezhang@ccmu.edu.cn (Y.Z.);

**Keywords:** *Pneumocystis jirovecii* pneumonia, prophylaxis, trimethoprim-sulfamethoxazole, non-HIV immunocompromised patients

## Abstract

*Pneumocystis jirovecii* pneumonia (PCP) prophylaxis is established in people with human immunodeficiency virus (HIV), but guidance for non-HIV, non-malignant immunocompromised adults remains fragmented. We searched eight databases and registries through 18 May 2026 (PROSPERO CRD420261396046). Because the evidence did not support a clinically exchangeable network, we evaluated four linked routes: comparative effectiveness, active-regimen safety, trimethoprim-sulfamethoxazole (TMP-SMX) discontinuation burden, and descriptive second-line evidence. Fifty-four full-length peer-reviewed reports were included. The primary effectiveness synthesis comprised 27 studies, 34,473 participants, and 243 PCP events. Compared with no prophylaxis, standard-dose TMP-SMX (odds ratio [OR] 0.30, 95% confidence interval [CI] 0.18–0.48; low certainty) and low-dose TMP-SMX (OR 0.08, 95% CI 0.03–0.18; low certainty) were associated with lower PCP incidence. Lower-intensity TMP-SMX strategies were associated with fewer treatment-limiting discontinuations than standard/conventional-dose TMP-SMX (OR 0.234, 95% CI 0.145–0.377). Across 33 TMP-SMX studies/cohorts, the pooled discontinuation proportion was 12.3% (95% CI 9.4–15.9%). Breakthrough PCP occurred in 0.5% of 1056 second-line recipients. TMP-SMX had the clearest protective association; however, low-dose evidence did not establish superiority or non-inferiority, and second-line data did not permit comparative estimates or regimen ranking.

## 1. Introduction

*Pneumocystis jirovecii* pneumonia (PCP) is associated with substantial mortality among immunocompromised adults, and non-HIV-associated PCP is generally more severe than HIV-associated PCP [1,2,3,4]. With the increasing use of systemic glucocorticoids, conventional and biologic immunosuppressants, and targeted therapies, the population at risk of PCP without HIV has expanded, creating an urgent need for evidence in these patients [5,6,7,8]. This review focuses on adults without HIV who have non-malignant conditions requiring immunosuppression, including rheumatic or autoimmune diseases and solid-organ transplantation. Autoimmune diseases are characterized by immune dysregulation and aberrant immune activation, whereas solid-organ transplantation involves immune responses to an allograft [9,10,11]. In both settings, the key common context for PCP is therapeutic immunosuppression. By contrast, malignancy and its treatment are often accompanied by multiple disease-related and treatment-related immune deficits that may vary over time [12]. We therefore excluded patients with malignancy to focus on a growing population in whom PCP risk is more clearly centered on therapeutic immunosuppression.

Trimethoprim-sulfamethoxazole (TMP-SMX) is the accepted first-line agent for PCP prophylaxis [13]. Much of the comparative evidence for PCP prophylaxis derives from populations with HIV or malignancy-related immunosuppression, whereas evidence on dose strategies, treatment-limiting discontinuation, and second-line prophylaxis remains limited in rheumatic disease and solid-organ transplant populations [14,15,16,17]. Direct extrapolation is uncertain because these populations differ substantially in the mechanisms of immune impairment, concomitant therapies, underlying PCP risk, and drug-toxicity profiles [18]. In non-HIV adults with non-malignant conditions, TMP-SMX tolerability is a particularly important but often underappreciated clinical challenge [19,20]. The overall real-world burden of TMP-SMX discontinuation due to adverse events remains poorly quantified [21]. When TMP-SMX cannot be continued, evidence for second-line agents, such as atovaquone, dapsone, and aerosolized pentamidine, remains limited [22,23,24].

Previous reviews have addressed overall prophylactic efficacy or specific dose schedules and agents [13,25], but they have not jointly examined regimen effectiveness, tolerability, discontinuation burden, and post-intolerance switching in non-HIV, non-malignant adults. Such fragmented evidence provides limited support for the sequential decisions encountered in clinical practice. We therefore conducted a systematic review and network meta-analysis (NMA) to address four interrelated questions: comparative effectiveness for preventing PCP (Route A–E), the safety of effective TMP-SMX dosing strategies (Route A–S), the burden of TMP-SMX discontinuation (Route B), and descriptive evidence for second-line agents after TMP-SMX intolerance or discontinuation (Route C). Rather than constructing a simple treatment hierarchy, this review aimed to provide a structured, population-specific evidence map that clarifies what can be concluded from the current data, where uncertainty remains, and how PCP prophylaxis can be better individualized for non-HIV, non-malignant immunocompromised adults.

## 2. Materials and Methods

### 2.1. Protocol and Eligibility

This systematic review and network meta-analysis was registered in the International Prospective Register of Systematic Reviews (PROSPERO; CRD420261396046) and conducted according to the Preferred Reporting Items for Systematic Reviews and Meta-Analyses (PRISMA) 2020 statement and the PRISMA extension for network meta-analysis. Eligible studies enrolled adult non-HIV, non-malignant immunocompromised patients receiving PCP prophylaxis or included in eligible no-prophylaxis, observation, or placebo groups. Eligible populations included rheumatic or autoimmune diseases, solid-organ transplantation, and long-term corticosteroid or immunosuppressive therapy. Eligible regimens included trimethoprim-sulfamethoxazole (TMP-SMX), atovaquone, dapsone, pentamidine, and no prophylaxis/observation/placebo. Since our focus is on treatment-related immunosuppression rather than disease-related or mixed immunosuppression, studies involving HIV infection, malignancy, hematopoietic stem-cell transplantation, primary immunodeficiency, pediatric populations, or inseparable mixed populations were excluded. Randomized and non-randomized comparative studies were eligible for comparative analyses; single-arm or descriptive studies were used only when they provided route-specific data for TMP-SMX discontinuation burden or second-line prophylaxis.

#### Search Strategy, Study Selection, and Data Extraction

PubMed/MEDLINE, Embase, Web of Science, the Cochrane Central Register of Controlled Trials (CENTRAL), China National Knowledge Infrastructure (CNKI), Wanfang, ClinicalTrials.gov, and the World Health Organization International Clinical Trials Registry Platform (WHO ICTRP) were searched from inception to 18 May 2026. Search terms combined PCP, prophylaxis, eligible agents, and non-HIV immunocompromised populations; full strategies are provided in Supplementary Table S1. Two reviewers independently screened records and assessed full reports using prespecified criteria. Data were extracted independently by XW and KW using a prespecified form. Disagreements, unclear cases, duplicate reports, overlapping cohorts, registry records, and route allocation were resolved by discussion and full-text adjudication; YZ and LZ participated in these decision discussions. The most complete report from an overlapping cohort was retained to avoid double counting. Extracted items included design, population, immunosuppressive context when reported, prophylaxis regimen and duration, comparator, TMP-SMX dose or strategy, follow-up, PCP definition, denominators, PCP events, treatment-limiting discontinuation, and second-line outcomes. Conference-only records were audited for a subsequent full-length peer-reviewed report. When a corresponding eligible full report was available, it was used as the sole source of quantitative data and the preliminary abstract was not counted separately. Abstract-only records without a corresponding eligible full report were retained in the screening and adjudication audit trail but excluded from all quantitative analyses.

### 2.2. Outcomes and Analytical Framework

The primary effectiveness outcome was confirmed or probable PCP. The primary safety and tolerability outcome was treatment-limiting discontinuation that the source study attributed to an adverse event (AE), adverse drug reaction (ADR), or toxicity. Secondary outcomes included all-cause and PCP-related mortality, total and serious AEs, specific toxicities, hypersensitivity, hyperkalemia, and breakthrough PCP, where reported.

For Routes A-S and B, AE, ADR, and toxicity were treated as source-study attribution terms rather than interchangeable clinical diagnoses. An event was included in the harmonized primary safety endpoint only when the report linked it to prophylaxis discontinuation, interruption, or inability to continue treatment. General AE counts, isolated laboratory abnormalities, and all-cause discontinuation were not used as substitutes for this endpoint. We retained each study’s original terminology and operational definition during extraction. This approach improved clinical comparability around treatment-limiting events; however, differences in attribution, severity thresholds, surveillance, follow-up, and clinician stopping decisions could still introduce outcome misclassification and heterogeneity.

Quantitative synthesis was organized into four protocol-informed routes according to comparator structure, outcome availability, and clinical context. Network meta-analysis (NMA) was restricted to Route A-E, in which four treatment nodes formed a connected primary effectiveness network. Within this connected network, NMA allowed direct and indirect evidence to be synthesized in a single framework, including estimation of the relative effect of low-dose versus standard-dose TMP-SMX when direct head-to-head evidence was limited. Where informative closed loops were available, agreement between direct and indirect evidence was also assessed. These network-level inferences could not be obtained from separate pairwise meta-analyses alone. Route A-S included only active TMP-SMX comparisons, Route B estimated the non-comparative burden of treatment-limiting discontinuation, and Route C remained descriptive. The sparse structure of the connected network and the clinical differences across studies were considered in the assessment of transitivity and in the interpretation of NMA estimates.

### 2.3. Interventions and Dose Classification

For the effectiveness network, treatment nodes were classified as standard/conventional-dose TMP-SMX, source-defined low/reduced-intensity TMP-SMX, pentamidine, and no prophylaxis/placebo. Dose classification used a source-relative, arm-level approach rather than a universal milligram threshold. The low/reduced-intensity category included regimens explicitly described by the source as low, reduced, half, or very low dose, as well as regimens clearly lower in dose or frequency than the study’s conventional or reference strategy, including reduced-frequency and dose-escalation regimens. Standard/conventional dosing comprised the study-defined reference regimen or a commonly used prophylactic schedule. Because terminology varied across study eras and clinical settings, the same absolute regimen could be described differently by different authors. Exact source terminology, dose/frequency, and classification rationale are reported in Supplementary Table S17; an unspecified regimen was never classified as low/reduced intensity. Across the A-E base case, A-S base case, and Route B main analysis, arm-level dose classification covered 46 unique studies and 56 unique TMP-SMX source arms: 18 arms were classified as low/reduced intensity, 19 had an extractable standard/conventional regimen, and 19 remained dose-unclear. Six dose-unclear arms entered the A-E standard/unspecified node in the base-case analysis. The strict-dose A-E sensitivity excluded eight studies/nine arms, including these six unclear-dose arms and three prespecified borderline or special-regimen arms. Atovaquone was excluded from the base-case effectiveness network because most data reflected second-line use after TMP-SMX intolerance or discontinuation; dapsone was not estimable in the connected base-case network.

### 2.4. Risk of Bias, Statistical Analysis, and Certainty Assessment

Risk of bias was assessed independently by XW and KW using the Cochrane Risk of Bias 2 tool (RoB 2) for randomized studies, Risk Of Bias In Non-randomized Studies of Interventions (ROBINS-I) for non-randomized comparative studies, and an adapted Joanna Briggs Institute (JBI)-style checklist for single-arm or descriptive studies. Differences were resolved through discussion involving XW, KW, YZ, and LZ until consensus. Because most included evidence was observational, the assessment explicitly considered confounding and selection bias, including baseline PCP risk, clinical indication for prophylaxis, dose selection, and treatment monitoring. Risk-of-bias and certainty assessments informed interpretation of the pooled estimates but could not statistically remove residual confounding from the study-level data (Supplementary Tables S5–S7 and S19). Certainty ratings were informed by the Grading of Recommendations Assessment, Development and Evaluation (GRADE) framework and Confidence in Network Meta-Analysis (CINeMA) principles [26]. All quantitative analyses and figure generation were performed using reproducible Python scripts (Python version 3.12.13) with NumPy (version 2.3.5), SciPy (version 1.17.0), pandas (version 2.2.3), and Matplotlib (version 3.10.8).

For Route A-S, study-specific log ORs were pooled using a random-effects inverse-variance model; study weight was therefore determined by the inverse of the estimated log-OR variance rather than by total sample size alone. When a study contained a zero cell, the prespecified continuity correction of 0.5 was applied to all four cells of its 2 × 2 table.

Before conducting the NMA, we assessed the plausibility of transitivity by comparing clinically important effect modifiers across treatment nodes, including population domain, transplant organ, baseline PCP risk, immunosuppressive regimen, selection into prophylaxis or second-line therapy, prophylaxis duration, follow-up, PCP ascertainment, and AE monitoring. These features were incompletely reported and were not evenly distributed (Supplementary Table S16); transitivity was therefore regarded as only partly plausible. This limitation was considered when interpreting indirect and pooled NMA estimates.

## 3. Results

### 3.1. Study Selection

**Figure 1:**
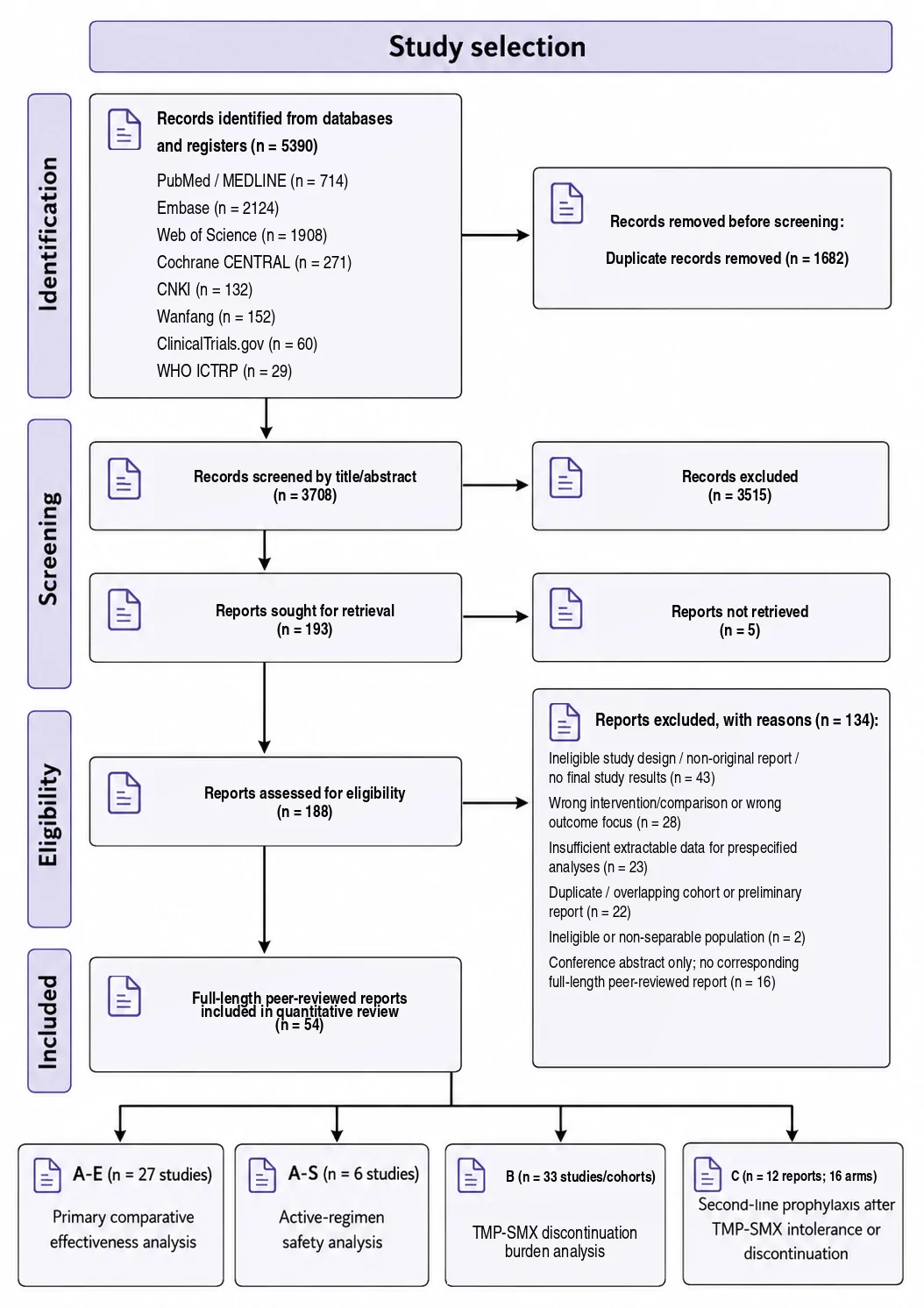
PRISMA flow diagram. Record identification, deduplication, screening, eligibility assessment, and final inclusion in the systematic review and quantitative analyses. Reasons for the 134 assessed-report exclusions are shown within the diagram; five sought reports were not retrieved. After separation of a falsely deduplicated conference-supplement cluster, 1682 duplicates were removed, 3708 records were screened, 193 reports were sought, 188 were assessed, and 54 full-length peer-reviewed reports were included. Route totals are non-additive. A-E, primary comparative effectiveness analysis; A-S, active-regimen safety analysis; CENTRAL, Cochrane Central Register of Controlled Trials; CNKI, China National Knowledge Infrastructure; PRISMA, Preferred Reporting Items for Systematic Reviews and Meta-Analyses; WHO ICTRP, World Health Organization International Clinical Trials Registry Platform.

The search strategy yielded 5390 records. After removing 1682 duplicates, 3708 records were screened by title and abstract, of which 3515 were excluded. Reports were sought for 193 records; 5 reports could not be retrieved, and 188 reports were assessed for eligibility. Of these, 134 were excluded after assessment, with reasons detailed in Supplementary Table S2. Ultimately, 54 full-length peer-reviewed reports were included in the quantitative synthesis (Figure 1).

**Figure 2:**
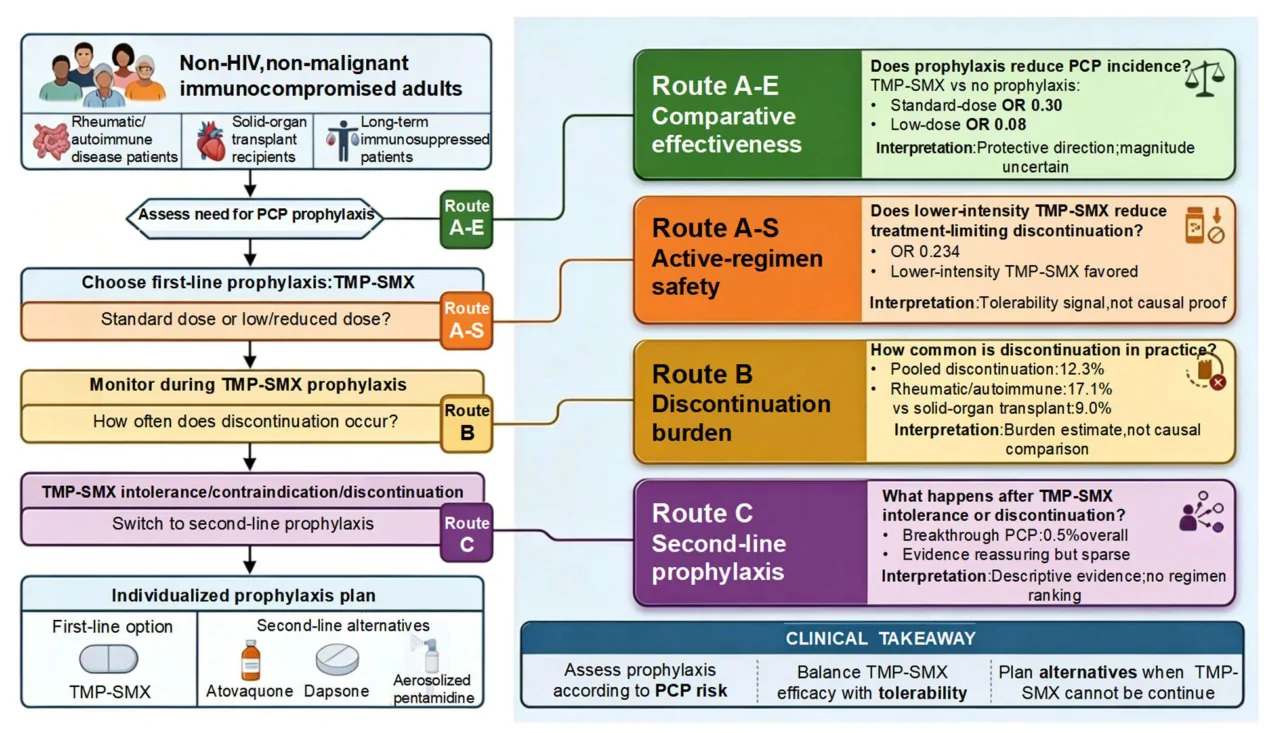
Clinical decision pathway and route-specific evidence synthesis for PCP prophylaxis in non-HIV, non-malignant immunocompromised adults. The left panel follows the sequence from prophylaxis indication through first-line TMP-SMX, treatment-limiting intolerance, and second-line selection. The right panel maps the four analytical routes: A-E comparative effectiveness, A-S active-regimen tolerability, B discontinuation burden, and C descriptive second-line outcomes. The evidence map supports interpretation and does not rank second-line regimens. PCP, *Pneumocystis jirovecii* pneumonia; TMP-SMX, trimethoprim-sulfamethoxazole. HIV, human immunodeficiency virus; OR, odds ratio.

These reports contributed to one or more predefined analytical routes within the final synthesis framework: 27 studies to Route A-E, 6 studies to Route A-S, 33 independent studies or cohorts to Route B, and 12 reports to Route C. Route totals are not additive because some reports contributed data to multiple routes. The sequential analytical framework is shown in Figure 2, and eligibility criteria and route-specific decisions are summarized in Supplementary Table S3.

### 3.2. Study and Population Characteristics

The characteristics of included studies are summarized in Table 1 and detailed in Supplementary Table S4. Reports were published between 1989 and 2025 and mainly involved rheumatic or autoimmune disease and solid-organ transplantation. Supplementary Table S4 now provides report-level information on population, prophylaxis regimen, comparator, prophylaxis duration, follow-up, baseline immunosuppressive therapy or clinical context, and PCP diagnostic or ascertainment criteria, with NR used when information was not reported. Reporting of these clinically important variables was incomplete and uneven across studies, and this limitation was considered when interpreting clinical heterogeneity and transitivity.

The primary comparative effectiveness dataset included 27 studies, 57 arms, 34,473 participants, and 243 PCP events. The active-regimen safety analysis included 6 studies, 12 pooled TMP-SMX comparison arms, 830 participants, and 137 treatment-limiting discontinuations. Route B included 33 independent studies or cohorts, 6996 TMP-SMX recipients, and 983 discontinuations. Route C included 12 reports, 16 second-line prophylaxis arms, 1056 recipients, and 5 breakthrough PCP events. These route-specific participant and event counts should not be summed across analyses.

### 3.3. Risk of Bias and Outcome Availability

Risk-of-bias and study-quality assessments are summarized in Supplementary Figures S1–S5 and Supplementary Tables S5–S10. Of the 54 reports, 43 (79.6%) were retrospective or observational, 5 (9.3%) were prospective or quality-improvement cohorts, and 6 (11.1%) were randomized or pilot randomized studies. The dominant concerns were confounding by indication, risk-based selection for prophylaxis, historical or non-concurrent controls, open-label stopping decisions, incomplete AE reporting, and sparse second-line evidence. Thus, the evidence supports cautious statements about the direction of the TMP-SMX association versus no prophylaxis, but it is substantially less secure for causal dose comparisons or active-regimen ranking.

Outcome availability is summarized in the supplementary outcome-feasibility tables. PCP incidence and discontinuation due to AEs, ADRs, or toxicity were the outcomes with sufficient extractable arm-level or route-specific denominator data for quantitative synthesis. Mortality, total AEs, specific toxicities, hyperkalemia, hypersensitivity, and breakthrough PCP outside the second-line setting were inconsistently reported, often lacked clear denominators, or had too few events for reliable network meta-analysis. These outcomes were therefore summarized narratively or not quantitatively synthesized where appropriate. Because contrasts contained few studies and events, reporting bias was judged qualitatively only; no funnel plot or small-study-effects test was undertaken.

### 3.4. Analytical Framework for Quantitative Synthesis

The quantitative synthesis was organized around the clinical sequence of PCP prophylaxis decisions in non-HIV, non-malignant immunocompromised adults. The first question was whether prophylaxis was associated with fewer PCP events than no prophylaxis, observation, or placebo. This was addressed in Route A-E, the primary comparative effectiveness analysis.

Because TMP-SMX is the main prophylactic agent in clinical practice but may be limited by adverse events, the second question was whether lower-intensity TMP-SMX strategies were associated with fewer treatment-limiting discontinuations than standard or conventional TMP-SMX dosing. This was addressed in Route A-S using active TMP-SMX comparator studies only.

Many clinically relevant studies reported TMP-SMX discontinuation without a comparator group. These studies could not estimate comparative safety but could answer a clinically important burden question: how often is TMP-SMX stopped because of adverse events, adverse drug reactions, or toxicity in eligible patients? Route B therefore quantified the observed TMP-SMX discontinuation burden and explored variation across population domains and dose or strategy categories.

Finally, when TMP-SMX cannot be continued, clinicians often switch to atovaquone, dapsone, or aerosolized pentamidine. Patients receiving these second-line agents are not exchangeable with first-line TMP-SMX recipients because they have already been selected by TMP-SMX intolerance, contraindication, toxicity, or discontinuation. Route C therefore summarized this evidence descriptively rather than combining it with the primary effectiveness network. Figure 2 maps these four routes to the sequential clinical decision pathway.

### 3.5. Route A-E: Primary Comparative Effectiveness Analysis

**Figure 3:**
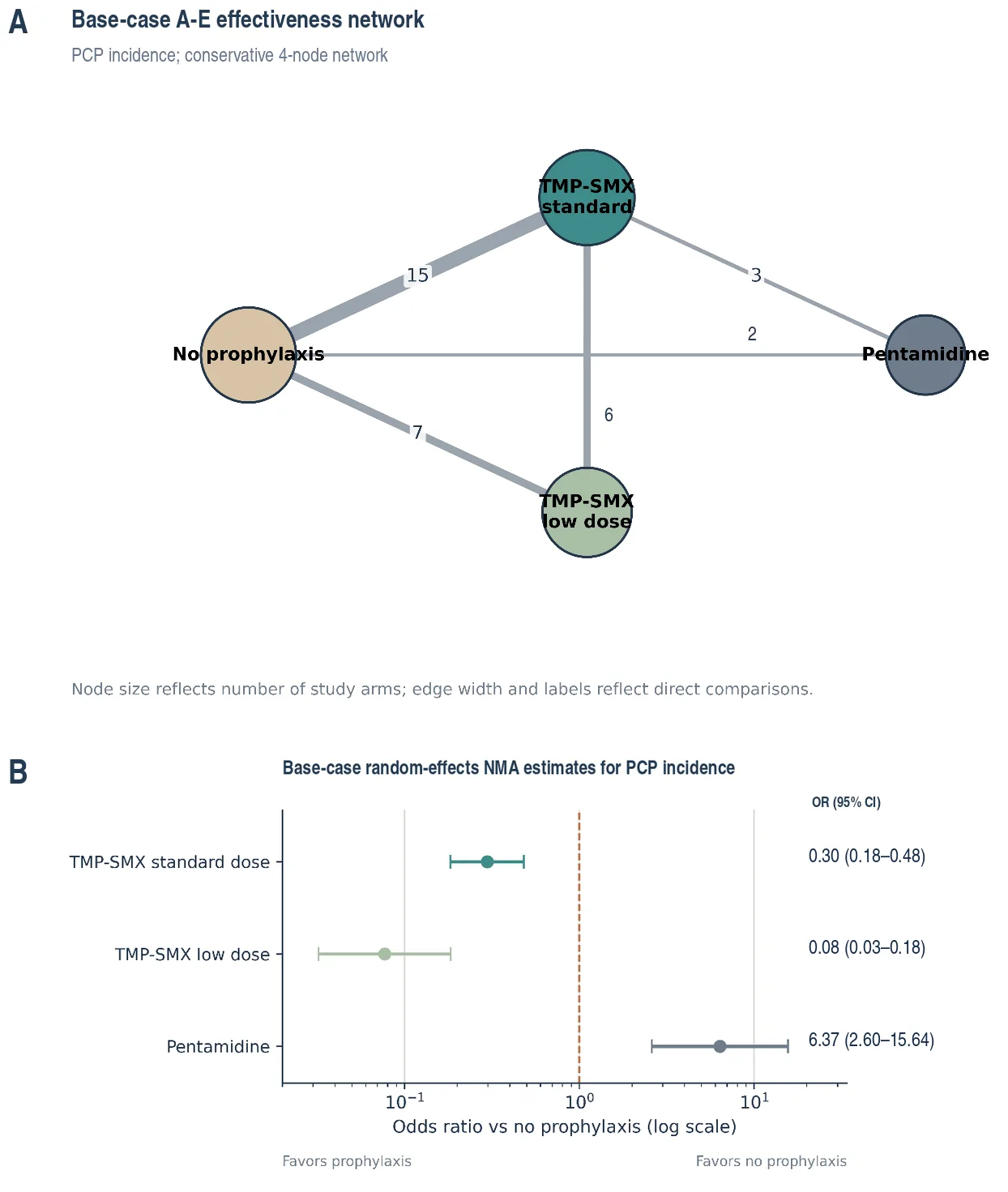
Primary comparative effectiveness network meta-analysis. (**A**) Network geometry. Node area is proportional to the number of study arms; edge width is proportional to the number of direct-comparison studies; edge labels give the direct-comparison study counts. At the edge crossing, labels are offset to identify each comparison. (**B**) Random-effects network ORs for PCP incidence, using no prophylaxis/observation/placebo as the reference. OR < 1 favors the active regimen. CI, confidence interval; OR, odds ratio; PCP, *Pneumocystis jirovecii* pneumonia; TMP-SMX, trimethoprim-sulfamethoxazole. A-E, primary comparative effectiveness analysis. In panel **B**, points are network ORs, horizontal bars are 95% CIs, and the dashed vertical line at OR = 1 denotes no effect.

Route A-E compared prophylaxis strategies for PCP incidence versus no prophylaxis, observation, or placebo. The analysis included 27 studies, 57 arms, 34,473 participants, and 243 PCP events in a connected network of four strategies: no prophylaxis/observation/placebo, standard-dose TMP-SMX, low-dose TMP-SMX, and pentamidine. Across the network, there was no appreciable statistical heterogeneity (tau^2^ = 0.0133; Cochran’s Q = 26.36, df = 27, *p* = 0.499; I^2^ = 0%) and no clear network-wide disagreement between direct and indirect evidence (design-by-treatment Q = 7.80, df = 5, *p* = 0.168). However, these overall tests do not exclude disagreement in an individual comparison, particularly because PCP events and informative closed loops were sparse. The network estimates are shown in Figure 3 and Table 2; direct comparisons, sensitivity analyses, inconsistency, transitivity, and certainty assessments are provided in Supplementary Figures S6–S11 and Supplementary Tables S11–S20.

The final A-E input contained 33 analytic TMP-SMX arms: 12 source-defined low/reduced-intensity arms and 21 standard/unspecified arms. The latter comprised 15 arms with an extractable standard/conventional regimen and six dose-unclear arms. No dose-unclear arm was classified as low/reduced intensity; complete arm-level regimens and classification rationales are provided in Supplementary Table S17. Sensitivity analyses explored the robustness of these node and dose-classification decisions.

Compared with no prophylaxis, observation, or placebo, TMP-SMX standard dose had a network OR of 0.30 (95% CI 0.18–0.48; low certainty), and TMP-SMX low dose had a network OR of 0.08 (95% CI 0.03–0.18; low certainty). Both TMP-SMX standard dose and low dose were associated with lower PCP incidence than no prophylaxis.

The network estimate comparing low-dose with standard-dose TMP-SMX was OR 0.26 (95% CI 0.10–0.66; very low certainty); thus, the low-dose node had the numerically more favorable point estimate. Participants who discontinued standard-dose TMP-SMX were not automatically reclassified into the no-prophylaxis node; treatment groups followed the source-study definitions, although post-discontinuation exposure handling was not uniform across studies. Both TMP-SMX dose nodes were associated with lower PCP incidence than no prophylaxis. Head-to-head evidence comparing the two doses was sparse, so much of the dose-to-dose estimate was obtained indirectly through their comparisons with the common no-prophylaxis reference. Moreover, the direct and indirect estimates disagreed (node-splitting *p* = 0.045). Clinically, the exact OR for low dose versus standard dose is therefore uncertain and should not be interpreted as establishing a causal efficacy advantage or non-inferiority.

Pentamidine had an unfavorable estimate compared with no prophylaxis, observation, or placebo (OR 6.37, 95% CI 2.60–15.64; very low certainty). This finding was based on sparse evidence and was likely affected by channeling or indication bias, because pentamidine was often used in patients who were not suitable for TMP-SMX. It should therefore not be interpreted as causal evidence that pentamidine is intrinsically harmful or inferior.

Atovaquone was not included as a base-case effectiveness node because available evidence largely reflected second-line use after TMP-SMX intolerance or discontinuation, making recipients clinically non-exchangeable with first-line prophylaxis or no-prophylaxis groups. Dapsone was prespecified as a candidate node but was not estimable in the connected base-case network. No regimen ranking was performed.

### 3.6. Route A-S: Active-Regimen Safety Analysis

Route A-S examined whether lower-intensity TMP-SMX strategies were associated with fewer treatment-limiting discontinuations than standard or conventional TMP-SMX dosing. This analysis included only studies with active TMP-SMX comparator arms; no-prophylaxis, observation, and placebo arms were excluded because participants in those arms had no prophylactic drug to discontinue.

**Figure 4:**
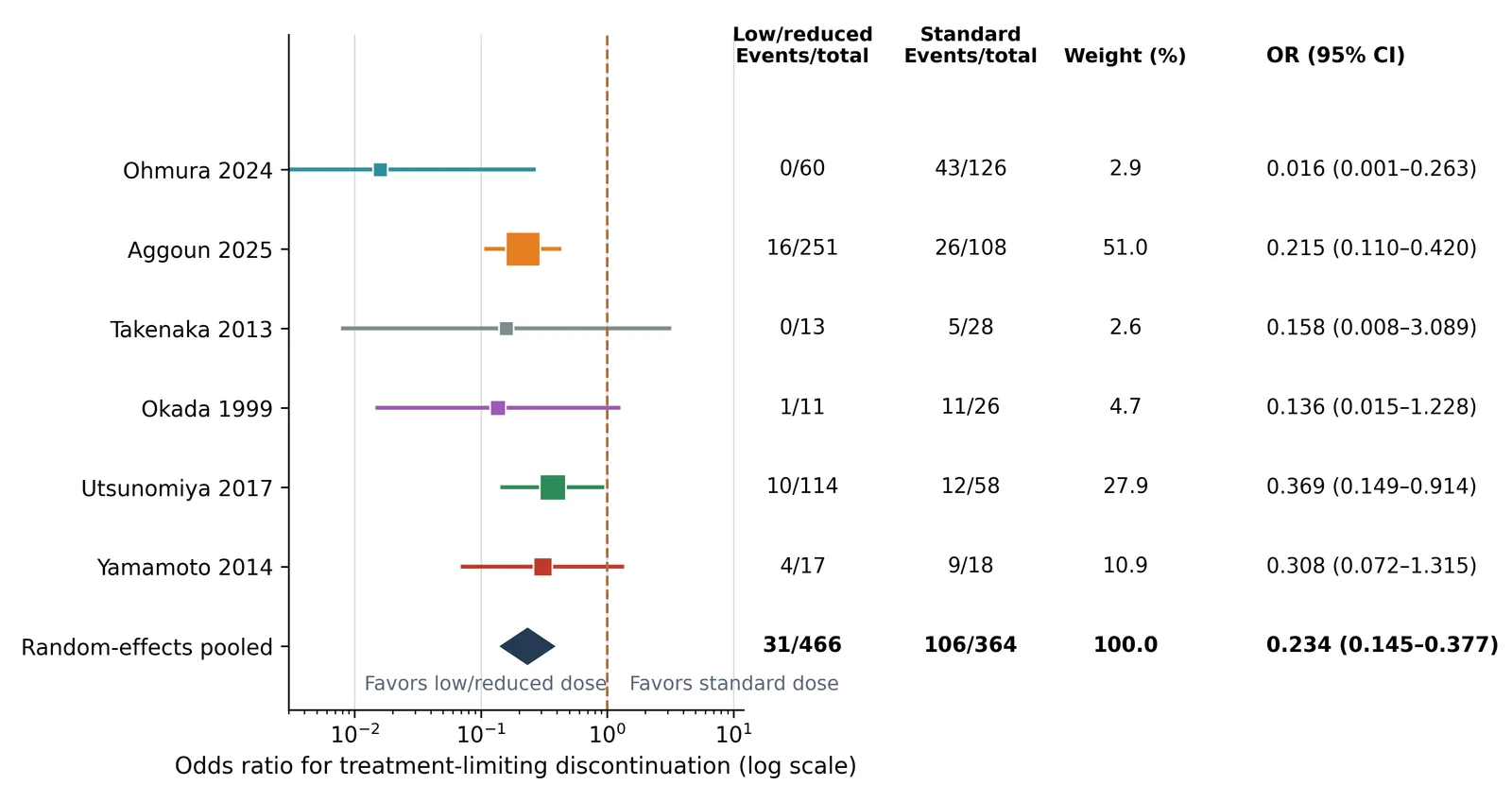
Active-regimen safety analysis: low/reduced-dose versus standard/conventional-dose TMP-SMX. Forest plot of ORs for discontinuation due to AEs. Square area is proportional to the inverse-variance weight of the study-level log OR; the displayed weights are derived from the same model and sum to 100%. OR < 1 favors low/reduced-dose TMP-SMX. The dashed vertical line at OR = 1 denotes no effect, and standard/conventional-dose TMP-SMX is the reference treatment. No-prophylaxis, observation, or placebo arms were excluded. AE, adverse event; OR, odds ratio; TMP-SMX, trimethoprim-sulfamethoxazole. Horizontal bars indicate 95% CIs, and the diamond represents the pooled random-effects estimate. CI, confidence interval [27,28,29,30,31,32].

The active-regimen safety analysis is shown in Figure 4 and summarized in Table 3, with sensitivity analyses in Supplementary Figure S12 and Supplementary Tables S21–S22. Across 6 studies and 12 active TMP-SMX arms, treatment-limiting discontinuation occurred in 31 of 466 participants (6.7%) receiving low/reduced-dose TMP-SMX and 106 of 364 participants (29.1%) receiving standard/conventional-dose TMP-SMX. In the pooled analysis, this corresponded to an OR of 0.234 (95% CI 0.145–0.377); an OR below 1 favors low/reduced-dose TMP-SMX. Statistical heterogeneity was not observed in this analysis (tau^2^ = 0; I^2^ = 0.0%), and sensitivity analyses retained the same direction of association.

This association is consistent with better tolerability of lower-intensity TMP-SMX, but it is not a causal estimate of a universally preferable dosing strategy. Dose selection, renal function, prior intolerance, population mix, monitoring intensity, and open-label stopping decisions could influence the observed discontinuation difference.

### 3.7. Route B: TMP-SMX Discontinuation Burden

Route B estimated the proportion of TMP-SMX recipients who discontinued prophylaxis due to adverse events, adverse drug reactions, or toxicity. Unlike Route A-S, this analysis included TMP-SMX arms or cohorts with extractable discontinuation data even when no comparator group was available. It therefore quantified observed burden rather than comparative regimen effects.

**Figure 5:**
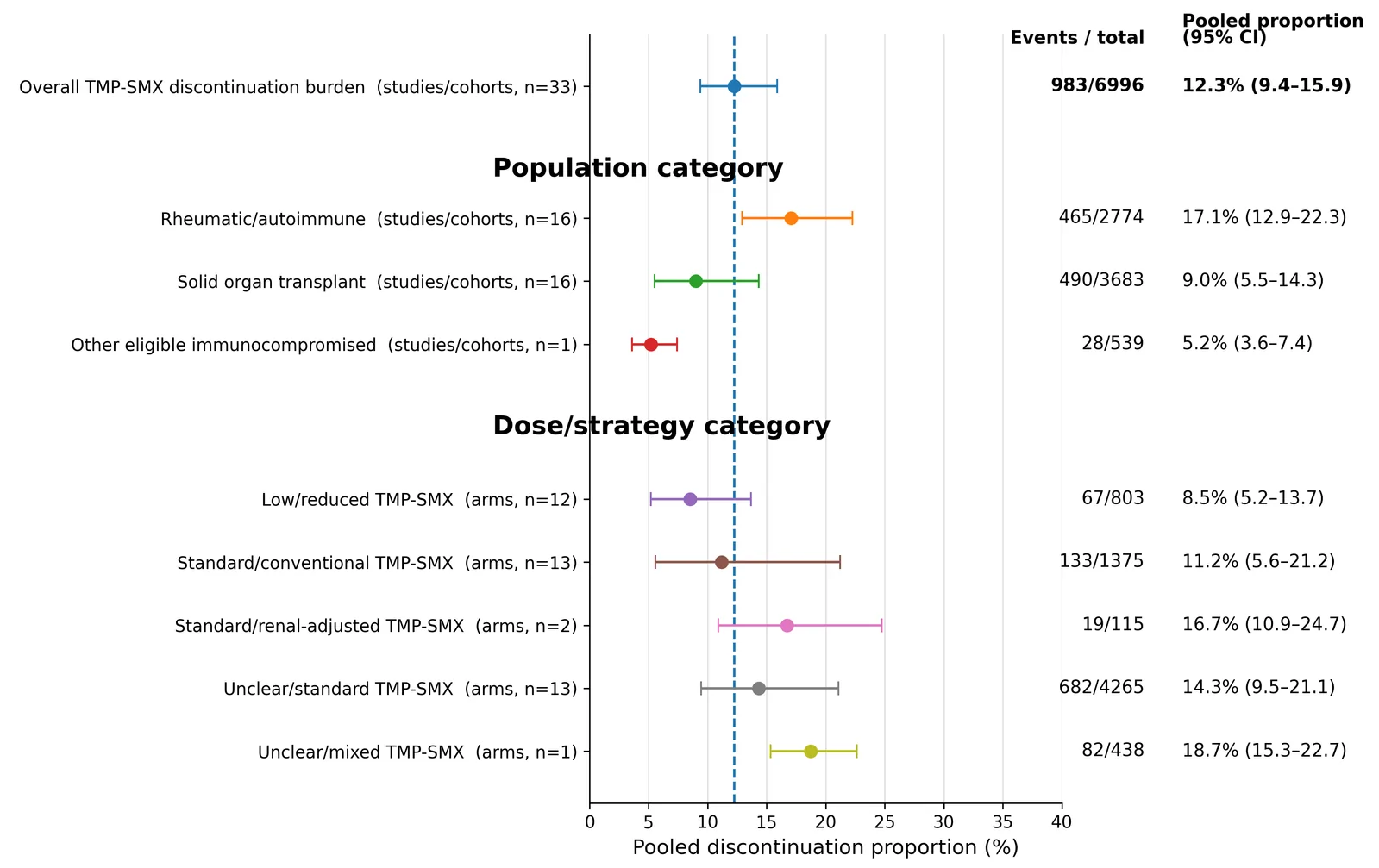
Subgroup pooled proportions of TMP-SMX treatment-limiting discontinuation attributed to adverse events, adverse drug reactions, or toxicity. Overall and population rows report independent studies/cohorts; dose/strategy rows report study arms and are arm-level descriptive analyses. Points represent pooled proportions, horizontal bars 95% CIs, and the dashed line the overall pooled proportion. Dose subgroup classifications reflect the final study-arm audit and do not support causal dose comparisons. CI, confidence interval; TMP-SMX, trimethoprim-sulfamethoxazole.

The TMP-SMX discontinuation burden analysis is summarized in Figure 5 and Table 3, with study-level results in Supplementary Figure S13 and Supplementary Tables S23–S26. Across 33 independent eligible TMP-SMX studies or cohorts, 983 discontinuations were reported among 6996 recipients. The pooled discontinuation proportion was 12.3% (95% CI 9.4–15.9%), with a prediction interval of 2.9–39.9% and substantial heterogeneity (I^2^ = 93.0%).

This finding indicates that TMP-SMX discontinuation is a clinically relevant burden rather than a rare event. Approximately one in eight TMP-SMX recipients discontinued prophylaxis because of adverse events, adverse drug reactions, or toxicity, although the wide prediction interval indicates that the expected burden may vary substantially across clinical settings.

Subgroup analyses showed pooled discontinuation proportions of 17.1% in rheumatic/autoimmune cohorts, 9.0% in solid-organ transplant cohorts, and 5.2% in other eligible immunocompromised cohorts. In the arm-level dose/strategy analysis, pooled proportions were 8.5% for low/reduced dose, 11.2% for standard/conventional dose, 16.7% for standard/renal-adjusted regimens, 14.3% for unclear/standard regimens, and 18.7% for the single unclear/mixed arm. The dose/strategy subgroup analysis used the final arm-level classifications; because the primary estimate was calculated at study level, it was unaffected by subgroup classification. These subgroup estimates are descriptive and do not establish comparative safety.

These subgroup findings should be interpreted as descriptive signals, not causal comparisons. Higher discontinuation in rheumatic or autoimmune disease cohorts may reflect differences in renal function, concomitant immunosuppressive drugs, monitoring intensity, prophylaxis duration, dose definitions, or discontinuation thresholds. Similarly, lower discontinuation in reduced-dose arms is consistent with the Route A-S tolerability signal, but Route B alone cannot prove comparative dose safety because it is based on single-arm proportions.

### 3.8. Route C: Second-Line Prophylaxis After TMP-SMX Intolerance or Discontinuation

Route C described breakthrough PCP among patients receiving second-line prophylaxis after TMP-SMX intolerance, contraindication, toxicity, or discontinuation. This evidence is summarized in Table 3, Supplementary Figure S14, and Supplementary Tables S27–S31. For Schumacher et al., the 2025 full-length peer-reviewed report was used, whereas the linked 2023 abstract was treated as preliminary and was not counted separately [33].

Across 12 reports and 16 second-line prophylaxis arms, 5 breakthrough PCP events occurred among 1056 recipients, corresponding to a crude proportion of 0.5% (exact 95% CI 0.2–1.1%). All 5 events occurred in atovaquone arms (5/495; 1.0%, exact 95% CI 0.3–2.3%). No events were observed in dapsone arms (0/335; exact 95% CI 0.0–1.1%) or aerosolized pentamidine arms (0/226; exact 95% CI 0.0–1.6%).

Breakthrough PCP was infrequently observed in these selected second-line cohorts, but this does not clinically validate atovaquone, dapsone, or pentamidine as equivalent alternatives. Events were sparse, the data were non-randomized or descriptive, recipients were selected after TMP-SMX intolerance or contraindication, and agent-specific follow-up and ascertainment differed. The zero-event dapsone and pentamidine arms must be read with their exact confidence limits, not as evidence of superiority. Route C therefore cannot estimate relative efficacy, establish equivalence to TMP-SMX, rank alternatives, or identify a preferred second-line agent; current choices remain dependent on indirect evidence, guideline context, contraindications, clinical experience, and patient-specific factors.

## 4. Discussion

The findings indicate that TMP-SMX has the clearest protective association against PCP compared with no prophylaxis, observation, or placebo, and that low-dose TMP-SMX also shows a protective direction. Treatment-limiting discontinuation of TMP-SMX is a clinically important burden that varies substantially across settings. For patients who cannot tolerate TMP-SMX or have a contraindication, the available evidence on atovaquone, dapsone, and pentamidine provides some context for second-line selection but remains insufficient to determine a preferred sequence of alternative regimens.

The population of non-HIV, non-malignant patients receiving therapeutic immunosuppression continues to expand, with a corresponding increase in the clinical need for evidence on PCP prophylaxis [5,34,35]. However, evidence in rheumatic or autoimmune disease and solid-organ transplant populations has remained fragmented across diseases, agents, dose strategies, and outcomes [36]. Previous syntheses have not jointly examined prophylactic effectiveness, dose strategies, treatment-limiting discontinuation, and outcomes after second-line switching within the same clinical pathway. To our knowledge, this is the first NMA to synthesize evidence on PCP prophylaxis across adults with rheumatic or autoimmune diseases and solid-organ transplant recipients within the shared clinical context of therapeutic immunosuppression. Its value does not lie in producing a complete hierarchy of all available agents. Rather, it integrates direct and indirect evidence for standard-dose TMP-SMX, low-dose TMP-SMX, pentamidine, and no prophylaxis within a connected four-node network; provides network-level estimates through a common comparator when direct head-to-head evidence is limited; and permits agreement between different evidence paths to be examined where informative loops are available. A series of separate pairwise meta-analyses would not have integrated this evidence structure or shown as clearly which clinical questions could support quantitative synthesis and which required descriptive interpretation.

A further strength is the separation of four questions that are frequently conflated along the complete clinical decision pathway. Route A-E evaluates comparative protection within the connected network; Route A-S compares treatment-limiting discontinuation between TMP-SMX dose strategies; Route B estimates the overall burden of treatment-limiting TMP-SMX discontinuation; and Route C describes outcomes after switching because of intolerance or discontinuation. This framework avoids placing single-arm discontinuation data or clinically selected second-line cohorts in the same treatment-ranking model. Broad searches of English-language and Chinese databases also captured dose-optimization and tolerability evidence that is particularly prominent in East Asian rheumatic and transplant practice [37,38]. Explicit node definitions, adjudication of overlapping populations, multiple sensitivity analyses, GRADE-CINeMA-informed certainty assessment, and an auditable analytical workflow further improve the transparency and reproducibility of the synthesis [39].

Comparison with previous systematic reviews and international guidelines further clarifies both the external consistency and the additional contribution of the present analysis. The 2014 Cochrane review included 13 randomized trials conducted mainly in patients with acute leukemia or solid-organ transplantation and also included pediatric participants. TMP-SMX reduced PCP occurrence by 85%; at a control-group risk of 6.2%, the number needed to treat was 19 (95% CI 17–42). The review found no clear increase in overall adverse events or events requiring discontinuation, but the relevant safety comparison was based on only four trials and 470 participants [40]. Our findings are concordant regarding the protective direction of TMP-SMX, while restricting the population to non-HIV, non-malignant adults and additionally quantifying treatment-limiting discontinuation and outcomes after second-line switching. The pooled discontinuation proportion of 12.3% and its wide prediction interval identify a clinically important, setting-dependent tolerability burden that was not well characterized in the earlier randomized evidence.

Current guidelines mainly define population-specific indications and duration rather than a comparative hierarchy of all prophylactic regimens. The 2022 European Alliance of Associations for Rheumatology recommendations state that prophylaxis appears beneficial in adults with autoimmune inflammatory rheumatic diseases receiving more than 15–30 mg/day of prednisolone or equivalent for more than 2–4 weeks, while emphasizing individualized risk assessment [7]. The American Society of Transplantation guideline recommends routine PCP prophylaxis for at least 6–12 months after solid-organ transplantation, preferably with TMP-SMX [41]. The present findings are consistent with these recommendations in supporting TMP-SMX as the best-supported first-line option when prophylaxis is indicated and add quantitative context concerning dose strategies, discontinuation, and second-line switching. Thus, in addition to being concordant with previous randomized evidence and current guidelines regarding the first-line role of TMP-SMX, the present study extends the evidence framework to encompass dose strategies, treatment-limiting discontinuation, and the sequential clinical pathway after switching to second-line prophylaxis.

Although the point estimates for both effectiveness and treatment-limiting discontinuation favored low-dose TMP-SMX, this finding was not robust. The low-dose versus standard-dose effectiveness comparison had very low certainty, sparse direct head-to-head evidence, a large indirect contribution, and disagreement between the direct and indirect estimates. The two dose nodes also contained different proportions of transplant and rheumatic cohorts and differed in baseline PCP risk, renal function, prescribing and monitoring practices, ability to remain on prophylaxis, and frequency of zero-event arms. Residual confounding and selective prescribing by clinicians could therefore make the low-dose node appear more favorable without representing a true pharmacological efficacy advantage.

Recent systematic reviews support the possibility that reduced or intermittent regimens improve tolerability but do not resolve the uncertainty about comparative efficacy. Masaki et al. included four randomized trials involving 2808 adults with and without HIV. PCP incidence did not differ significantly between intermittent and daily regimens (RR 1.17, 95% CI 0.89–1.53; very-low-certainty evidence), whereas intermittent dosing reduced adverse events requiring temporary or permanent discontinuation (RR 0.51, 95% CI 0.42–0.61; low-certainty evidence) [24]. Huang et al. included 17 predominantly low- to moderate-quality studies and 4890 HIV-uninfected patients. PCP events were rare and appeared similar between dose groups, whereas low-dose TMP-SMX was associated with fewer discontinuations (OR 0.38, 95% CI 0.27–0.52) [20]. The Route A-S result is directionally consistent with both reviews regarding tolerability. Taken together, the available evidence indicates that dose reduction has not been associated with a clear observed loss of protection, but it does not establish greater efficacy for low-dose TMP-SMX or demonstrate non-inferiority within a prespecified margin.

Tolerability is central to whether PCP prophylaxis can be continued through the intended period of risk. Because individual adverse reactions differed substantially in their definitions, severity, and reporting, we did not combine all adverse events, isolated laboratory abnormalities, or all-cause discontinuations as equivalent outcomes. We included only source-attributed adverse event-, adverse drug reaction-, or toxicity-related events that caused prophylaxis to be stopped or interrupted. Even under this more restrictive definition, studies differed in causality assessment, laboratory surveillance, follow-up, and clinician thresholds for stopping treatment. The pooled proportion of 12.3% should therefore be interpreted as an average observed burden of treatment-limiting discontinuation across heterogeneous practice settings rather than as a universal toxicity rate.

Notably, the pooled discontinuation proportion was 17.1% in rheumatic or autoimmune disease cohorts, approximately 1.9 times the 9.0% observed in solid-organ transplant cohorts. This is a descriptive setting-level signal and does not establish population type as an independent cause of discontinuation. Nevertheless, it suggests that clinicians initiating and maintaining TMP-SMX prophylaxis in patients with rheumatic or autoimmune diseases should pay particular attention to tolerability, concomitant medications, and laboratory monitoring.

The second-line data document clinical outcomes after TMP-SMX intolerance, contraindication, or discontinuation but do not yet define a preferred sequence of alternative regimens. Few studies have evaluated second-line prophylaxis specifically in non-HIV, non-malignant immunocompromised adults, events were sparse, and the denominators consisted of patients who had already developed intolerance or had been selected to receive a particular alternative. Atovaquone, dapsone, and aerosolized pentamidine cohorts also differed in indication, organ type, previous toxicity, follow-up, and outcome ascertainment [42,43]. The unfavorable pentamidine estimate in the primary network is more plausibly explained by channeling and confounding by indication, because pentamidine is commonly reserved for patients in whom TMP-SMX is unsuitable, than by an intrinsic harmful effect [44,45].

Current second-line selection therefore continues to rely mainly on indirect evidence, population-specific guidance, contraindications, clinical experience, and individual patient factors. Within the constraints imposed by the available study designs, real-world clinical data, and sparse events, Route C provides the most systematic description possible of clinical outcomes after switching to second-line prophylaxis; it is not intended to determine the relative merits of the alternative agents.

Chiu and Ching reported a pooled breakthrough PCP incidence of 0.7% (95% CI 0.3–1.4%) during intravenous pentamidine prophylaxis, but their evidence was derived mainly from patients with hematologic malignancies or hematopoietic stem-cell transplantation [22]. Because those populations were outside our eligibility criteria, that estimate is best regarded as contextual information about breakthrough during alternative prophylaxis and cannot be used to compare pentamidine with TMP-SMX, atovaquone, or dapsone in the present population. Decisions concerning prophylaxis initiation, dose adjustment, duration, and alternative-agent selection should therefore remain anchored to population-specific guidance and individualized PCP risk, tolerability, comorbidities, concomitant therapy, and monitoring feasibility.

Several limitations remain. First, 79.6% of the reports were retrospective or observational. Confounding by indication, risk-based prescribing, historical controls, and selection into different dose or alternative-treatment strategies may distort both effectiveness and tolerability estimates [44,45]. Patients at lower risk, with better adherence, or under closer monitoring may have been more likely to receive or remain on a reduced regimen, whereas frailer or intolerance-prone patients may also have been channeled to low dose; either mechanism could alter the apparent low-dose effect. Risk-of-bias assessment and certainty downgrading identify these concerns but cannot remove them from aggregated study-level data. Causal comparisons between dose nodes therefore remain uncertain. In addition, because dose terminology varied according to study era and clinical context, the nodes represent source-defined dosing strategies rather than universal absolute-dose thresholds, further limiting exchangeability and the interpretation of active-dose comparisons.

Second, clinical heterogeneity limits transitivity. No single validated cross-population measure was available to harmonize baseline PCP risk between the included rheumatic or autoimmune disease and solid-organ transplant cohorts. Studies also differed in transplanted organ, immunosuppressive regimen and its intensity and duration, prophylaxis indication and duration, renal function, concomitant therapy, follow-up, PCP ascertainment, adverse-event monitoring, and stopping rules. Because these potential effect modifiers were incompletely reported and unevenly distributed across treatment nodes, transitivity was considered only partly plausible. Low statistical heterogeneity does not establish clinical comparability, and population or dose subgroup analyses cannot restore exchangeability or adjust for all between-study differences. Pooled estimates, particularly cross-population indirect contrasts, comparisons between active regimens, and absolute burdens, should therefore be interpreted cautiously.

Third, PCP events were sparse, several study arms had no events, few network loops were informative, and some estimates depended mainly on indirect evidence [26,46]. Fourth, safety reporting was incomplete and heterogeneous. The harmonized treatment-limiting discontinuation rule improved outcome consistency but may still combine different causality and severity thresholds, whereas mortality and specific toxicities frequently lacked comparable denominators or follow-up. Route B consequently retained substantial heterogeneity, and Route C remained descriptive. Publication and availability bias also cannot be excluded despite broad database and registry searches. Abstract-only reports without an eligible full-length publication were retained only in the screening and adjudication audit trail and were excluded from all quantitative analyses.

Future studies should prioritize pragmatic comparative evaluations of TMP-SMX dose strategies in clearly defined non-HIV, non-malignant populations. They should prespecify baseline PCP risk, immunosuppressive exposure, prophylaxis duration, diagnostic criteria, treatment-limiting toxicity, renal adjustment, rechallenge, switching, and completion of the intended high-risk period. Properly designed non-inferiority trials are needed to determine whether reduced regimens preserve prophylactic efficacy within a clinically acceptable margin. Prospective registries could also capture the complete sequential pathway from prophylaxis initiation through dose adjustment or second-line switching.

## 5. Conclusions

TMP-SMX remains the most evidence-supported prophylactic agent for non-HIV, non-malignant immunocompromised adults when PCP prophylaxis is clinically indicated, and low-dose TMP-SMX also shows a protective direction. However, the available evidence remains insufficient to determine the optimal dose strategy. Treatment-limiting discontinuation is sufficiently common to affect successful completion of prophylaxis and varies substantially across clinical settings. Second-line evidence provides useful context when TMP-SMX is not tolerated or is contraindicated but does not yet establish a preferred sequence of alternative agents. Decisions should integrate underlying PCP risk, population-specific guidance, TMP-SMX tolerability, comorbidities, concomitant therapy, and monitoring feasibility.

## Figures and Tables

**Table 1:** Study characteristics.

Characteristic	Category	Studies/Records, *n* (%)	Participants/Events	Notes
Overall	Included full-length peer-reviewed reports contributing to ≥1 quantitative analysis	54		Report-level entries; reports could contribute to more than one route.
Overall	Publication years	1989–2025		Based on first publication year recorded in the screening/result files.
Analysis contribution	Primary comparative effectiveness analysis (A-E)	27 studies	243/34,473 PCP events/partici⁠pants across 57 arms	Base-case network: no prophylaxis/observation/placebo, TMP-SMX standard dose, TMP-SMX low dose, and pentamidine.
Analysis contribution	Active-regimen safety analysis (A-S)	6 studies	137/830 discontinuations/partici⁠pants across 12 TMP-SMX arms	Comparison of low/reduced-dose versus standard/conventional-dose TMP-SMX.
Analysis contribution	TMP-SMX discontinuation burden (B)	33	983/6996	Single-arm burden meta-analysis of 33 independent studies/cohorts; conference abstracts excluded.
Analysis contribution	Second-line prophylaxis (C)	12 reports	5/1056; 16 arms	Descriptive analysis after TMP-SMX intolerance/discontinuation.
Population domain	Rheumatic/autoimmune disease	25 (46.3%)		Assigned from extracted clinical subgroup.
Population domain	Solid organ transplant	29 (53.7%)		Assigned from extracted clinical subgroup. Schumacher 2025
Study design	Randomized or pilot randomized trial	6 (11.1%)		Harmonized from route-specific extraction tables.
Study design	Prospective or quality-improvement cohort	5 (9.3%)		Harmonized from route-specific extraction tables.
Study design	Retrospective/observational cohort or registry	43 (79.6%)		Harmonized from route-specific extraction tables. Schumacher 2025
Outcome contributed	PCP incidence	27 studies	243/34,473	Primary effectiveness outcome for A-E.
Outcome contributed	AE-/ADR-/toxicity-related discontinuation	A-S: 6; B: 33	A-S: 137/830; B: 983/6996	A-S was comparative; B was single-arm/subgroup proportion analysis.
Outcome contributed	Breakthrough PCP during second-line prophylaxis	12 reports	5/1056; 16 arms	Route C was descriptive and not used for comparative NMA.

Abbreviations: A-E, primary comparative effectiveness analysis; A-S, active-regimen safety analysis; NMA, network meta-analysis; PCP, *Pneumocystis jirovecii* pneumonia; TMP-SMX, trimethoprim-sulfamethoxazole. Note: The evidence set comprises 54 full-length peer-reviewed reports. Report-level population, regimen, comparator, prophylaxis duration, follow-up, baseline immunosuppressive therapy or clinical context, PCP diagnostic or ascertainment criteria, and route-specific outcomes are detailed in Supplementary Table S4; Related diagnostic and reporting limitations are further summarized in Supplementary Tables S5–S10.

**Table 2:** Primary comparative effectiveness network meta-analysis of PCP prophylaxis regimens with certainty of evidence.

Comparison	OR	95% CI	Certainty	Interpretation
TMP-SMX standard dose vs. no prophylaxis/observation/placebo	0.30	0.18–0.48	Low	Low-certainty protective association; direct, network, and RR sensitivity estimates were concordant.
TMP-SMX low dose vs. no prophylaxis/observation/placebo	0.08	0.03–0.18	Low	Low-certainty protective association; the apparent magnitude should be interpreted cautiously because of sparse events and transitivity concerns.
TMP-SMX low dose vs. TMP-SMX standard dose	0.26	0.10–0.66	Very low	Very-low-certainty, partly indirect evidence; neither superiority nor non-inferiority is established.
Pentamidine vs. no prophylaxis/observation/placebo	6.37	2.60–15.64	Very low	Very-low-certainty unfavorable estimate, likely affected by channeling or indication bias; not causal evidence of harm or intrinsic inferiority.

Abbreviations: CI, confidence interval; OR, odds ratio; RR, risk ratio. Note: Certainty ratings follow GRADE-CINeMA principles and refer to confidence in the direction of association rather than the precise magnitude. The primary model used odds ratios due to sparse events and zero-event arms; risk-ratio sensitivity analyses were used as robustness checks.

**Table 3:** Extended analyses summary: active-regimen safety, TMP-SMX discontinuation burden, and second-line prophylaxis. Route B and Route C values in this table reflect the revised main analyses excluding conference abstracts.

Route	Outcome	Evidence Base	Studies/ Arms	Participants/ Events	Model	Main Estimate	Heterogeneity/ Checks	Interpretation
A-S	AE-related discontinuation	Low/reduced vs standard TMP-SMX	6 studies; 12 pooled arms	31/466 vs. 106/364	Random-effects inverse-variance OR	OR 0.234 (95% CI 0.145–0.377)	tau^2^ = 0; I^2^ = 0.0%; sensitivity direction unchanged	Association only; selection, residual confounding, and open-label stopping decisions may bias the estimate.
B	AE/ADR/toxicity discontinuation	Eligible TMP-SMX cohorts	33 studies/cohorts	983/6996	Random-effects logit single-arm proportion	12.3% (95% CI 9.4–15.9%); PI 2.9–39.9%	I^2^ = 93.0%; audited dose subgroups in Figure 5	Observed burden, not a dose comparison; source definitions were harmonized only for treatment-limiting cessation.
C	Breakthrough PCP after intolerance	Atovaquone, dapsone, or pentamidine	12 reports; 16 arms	5/1056	Crude exact-binomial proportions	Overall 0.5% (0.2–1.1%); atovaquone 5/495; dapsone 0/335; pentamidine 0/226	Sensitivity excluding Jinno 2022: 0/949	Selected descriptive cohorts only; no clinical validation, equivalence, relative efficacy, or ranking.

Abbreviations: AE, adverse event; ADR, adverse drug reaction; CI, confidence interval; OR, odds ratio; PI, prediction interval; TMP-SMX, trimethoprim-sulfamethoxazole. Note: Events and participants are route-specific and non-additive. Route C uses crude exact-binomial proportions. No second-line regimen ranking was performed.

## Data Availability

All data analyzed in this review were extracted from published reports, trial registry records, or other publicly available study records. No new individual patient-level data were generated. Extracted aggregate data, analysis inputs, analytic code, and methodological documentation are provided in the Supplementary Materials and accompanying reproducibility package.

## Supplementary Materials

The following supporting information can be downloaded at: https://www.mdpi.com/article/10.3390/jof12080627/s1, Figure S1: Risk-of-bias and study-quality summary by analysis route. Route C includes the Schumacher 2025 full report and excludes conference abstracts. Route totals are not additive because some reports contributed to more than one route; Figure S2: RoB 2 traffic-light plot for randomized or pilot randomized studies. Domains follow the Cochrane RoB 2 framework; Figure S3: ROBINS-I traffic-light plot for non-randomized comparative studies. Domains follow the ROBINS-I framework; Figure S4: Adapted JBI traffic-light plot for single-arm/descriptive studies, part 1. Conference abstracts are excluded from the main evidence set; Figure S5: Adapted JBI traffic-light plot for single-arm/descriptive studies, part 2. Schumacher 2025 is included as a full peer-reviewed Route C report; Figure S6: Direct pairwise forest plot of TMP-SMX standard dose versus no prophylaxis/observation/placebo for PCP incidence. Odds ratios below 1 favor TMP-SMX standard-dose prophylaxis; Figure S7: Direct pairwise forest plot of TMP-SMX low dose versus no prophylaxis/observation/placebo for PCP incidence. Odds ratios below 1 favor low-dose TMP-SMX prophylaxis. The estimate should be interpreted in the context of sparse events and transitivity concerns; Figure S8: Direct pairwise forest plot of TMP-SMX low dose versus TMP-SMX standard dose for PCP incidence. Direct evidence did not demonstrate superiority of low-dose over standard-dose TMP-SMX; the network estimate favoring low-dose should therefore be interpreted cautiously; Figure S9: Direct pairwise forest plot of pentamidine versus no prophylaxis/observation/placebo for PCP incidence. Evidence was sparse and potentially affected by channeling or indication bias; the estimate should not be interpreted as causal evidence of harm or inferiority; Figure S10: Sensitivity analysis of the primary comparative effectiveness network meta-analysis. Forest plot of sensitivity network meta-analysis estimates for PCP incidence under alternative node or study-inclusion assumptions; Figure S11: Atovaquone sensitivity network for the primary comparative effectiveness analysis. Network geometry after including atovaquone as a sensitivity node. Atovaquone was not included in the base-case effectiveness network because available data largely reflected second-line use after TMP-SMX intolerance or discontinuation; Figure S12: Sensitivity analyses for the active-regimen safety analysis. Forest plot of sensitivity analyses comparing discontinuation due to adverse events between low-dose and standard-dose TMP-SMX; Figure S13: Study-level forest plot of TMP-SMX discontinuation due to adverse events across 33 eligible independent studies/cohorts. The primary analysis excludes conference abstracts; Figure S14: Crude breakthrough PCP proportions during second-line prophylaxis after TMP-SMX intolerance, contraindication, or discontinuation. The main analysis includes 12 reports and 16 arms. ‘Crude aggregate’ and ‘Crude overall’ are simple event/denominator summaries with exact binomial 95% CIs, not meta-analytic pooled effects. Conference abstracts are excluded; Table S1: Full search strategies by database and registry; Table S2: Reports excluded after eligibility assessment, with reasons (n = 134); Table S3: Eligibility criteria and analysis-route decision framework; Table S4: Detailed characteristics of included study records; Table S5: Risk-of-bias and study-quality assessment of included studies; Table S6: Risk-of-bias and study-quality assessment by analysis route and outcome; Table S7: Limited-information and unresolved reporting items; Table S8: Outcome availability and feasibility of quantitative synthesis; Table S9: Protocol-specified outcomes and final analytical handling; Table S10: Mapping of protocol-specified outcomes to final analysis routes; Table S11: A-E node definitions and base-case input summary; Table S12: Base-case network meta-analysis estimates and league table for PCP incidence; Table S13: Direct pairwise evidence supporting the A-E base-case network; Table S14: Heterogeneity diagnostics for the primary effectiveness NMA and key direct comparisons; Table S15: Inconsistency and node-splitting diagnostics for the A-E network; Table S16: Transitivity and node-adjudication table; Table S17: Sensitivity analyses for the A-E primary comparative effectiveness network meta-analysis; Table S18: Effect-measure and protocol-consistency assessment; Table S19: GRADE-CINeMA-informed certainty of evidence for the primary comparative effectiveness analysis; Table S20: Certainty rationale, interpretation constraints, and summary of findings; Table S21: A-S base-case input and model output; Table S22: A-S sensitivity analyses and interpretation constraints; Table S23: Route B arm-level extraction and base-case input summary; Table S24: Overall, population-subgroup, and dose-category analyses of TMP-SMX discontinuation; Table S25: Reported reasons for TMP-SMX discontinuation; Table S26: Route B sensitivity analyses and candidate-status decisions; Table S27: Candidate study decisions for second-line prophylaxis; Table S28: Arm-level extraction dataset for breakthrough PCP during second-line prophylaxis; Table S29: Breakthrough PCP summary and sensitivity analyses; Table S30: Descriptive second-line safety and toxicity findings.
