# Supplementary material for: Prophylaxis Regimens for Pneumocystis jirovecii Pneumonia in Non-HIV, Non-Malignant Immunocompromised Adults: A Systematic Review and Network Meta-Analysis
Source: J Fungi (Basel). 2026 Aug 20;12(8):627. doi: 10.3390/jof12080627 (PMC13514556; doi:10.3390/jof12080627)
Supplement: Supplementary file 1 [file jof-12-00627-s001.zip › jof-4394804-supplementary.pdf]

Catalogue

Supplementary Figures S1–S14 1
Supplementary Tables S1–S31 12
Section S1. Search strategy and study selection 12
Section S2. Study characteristics 39
Section S3. Risk of bias and evidence quality 52
Section S4. Outcome availability and analytical handling 59
Section S5. Route A-E: primary comparative effectiveness analysis 67
Section S6. Route A-S: active-regimen safety analysis 84
Section S7. Route B: TMP-SMX discontinuation burden 86
Section S8. Route C: second-line prophylaxis after TMP-SMX intolerance or discontinuation 92

Supplementary Figure

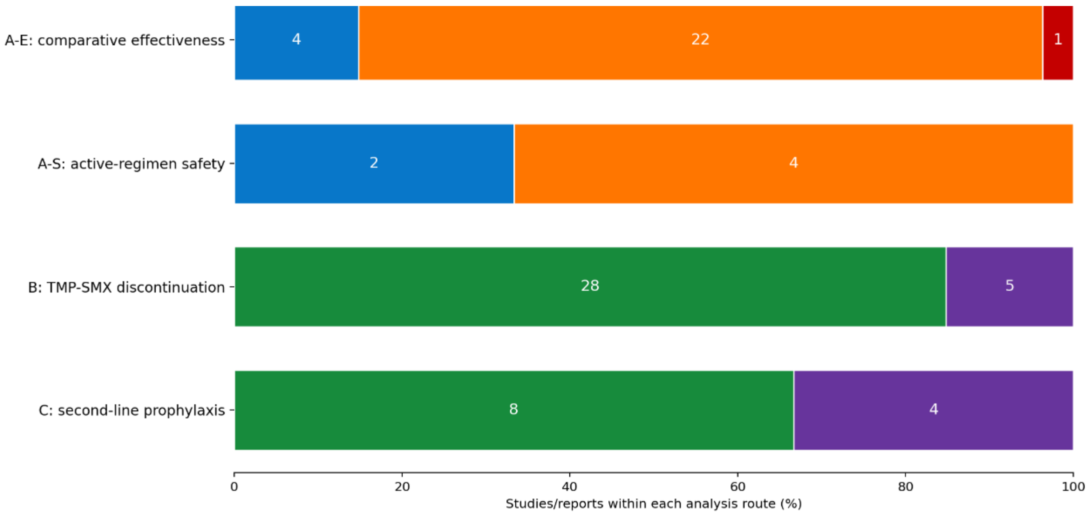

Supplementary Figure S1. Risk-of-bias and study-quality summary by analysis route. Route C includes the Schumacher 2025 full report and excludes conference abstracts. Route totals are not additive because some reports contributed to more than one route.

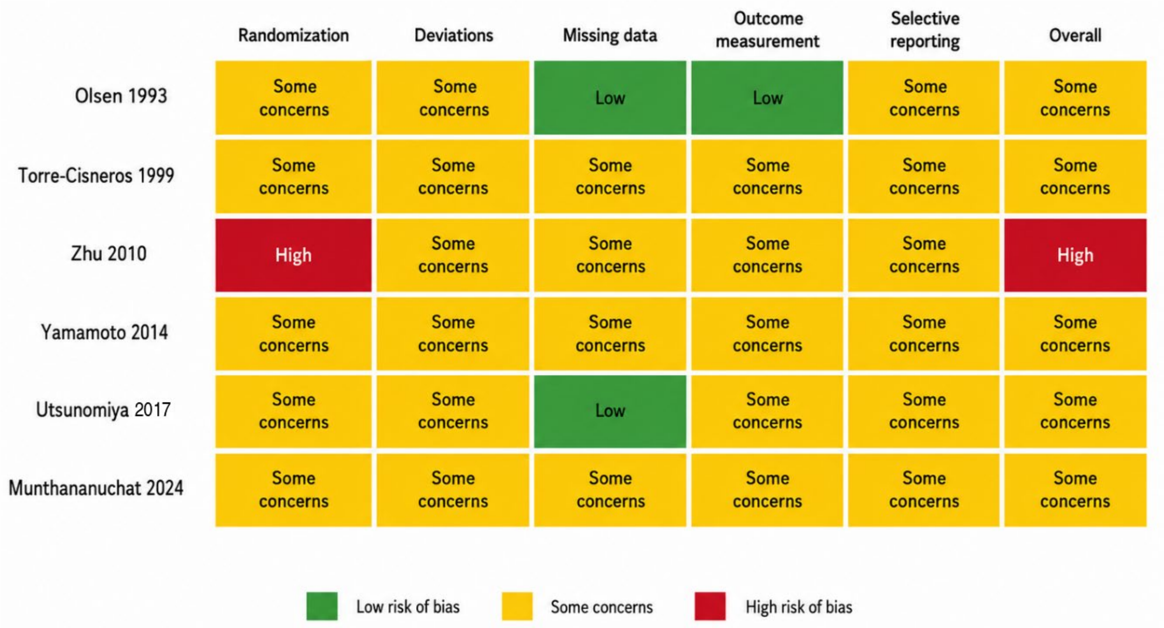

Supplementary Figure S2. RoB 2 traffic-light plot for randomized or pilot randomized studies. Domains follow the Cochrane RoB 2 framework.

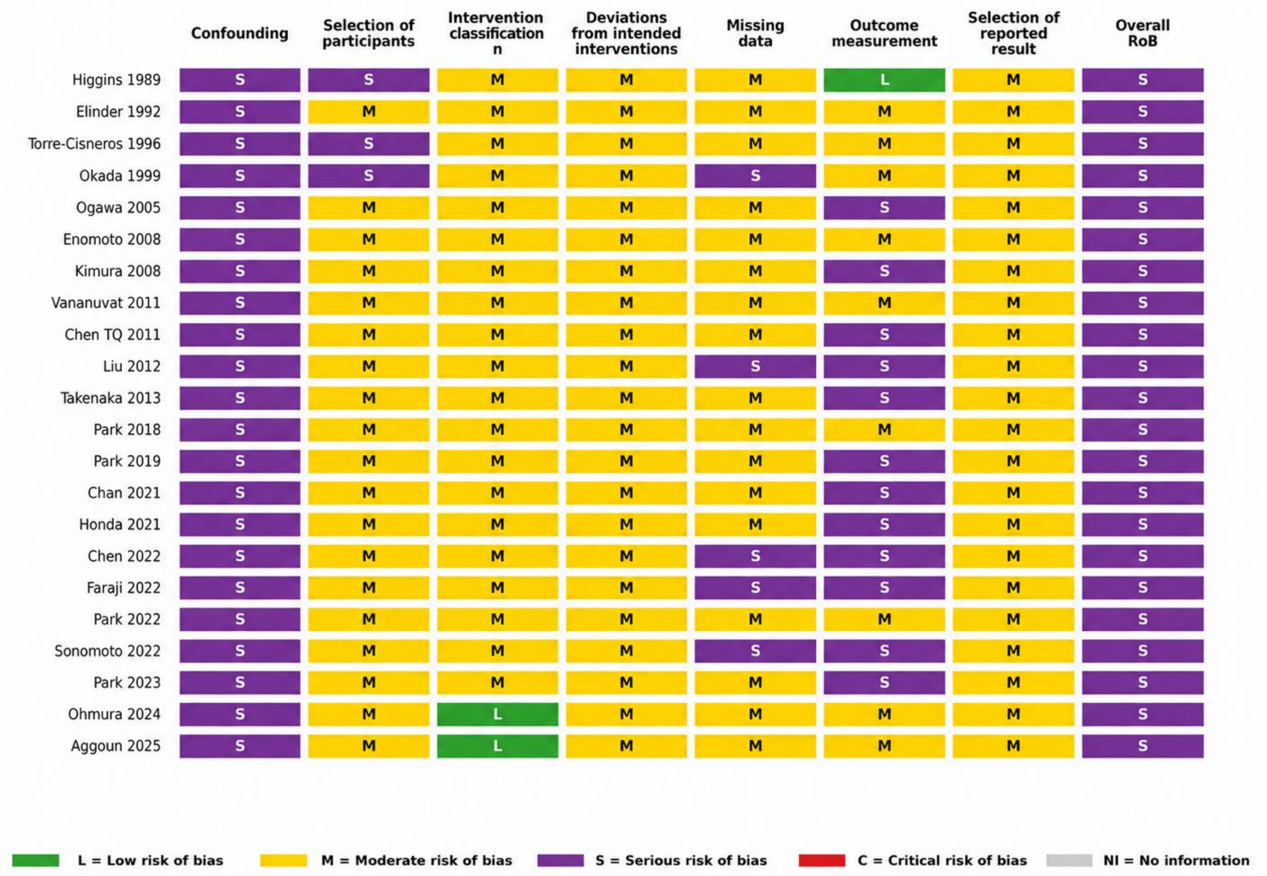

Supplementary Figure S3. ROBINS-I traffic-light plot for non-randomized comparative studies. Domains follow the ROBINS-I framework.

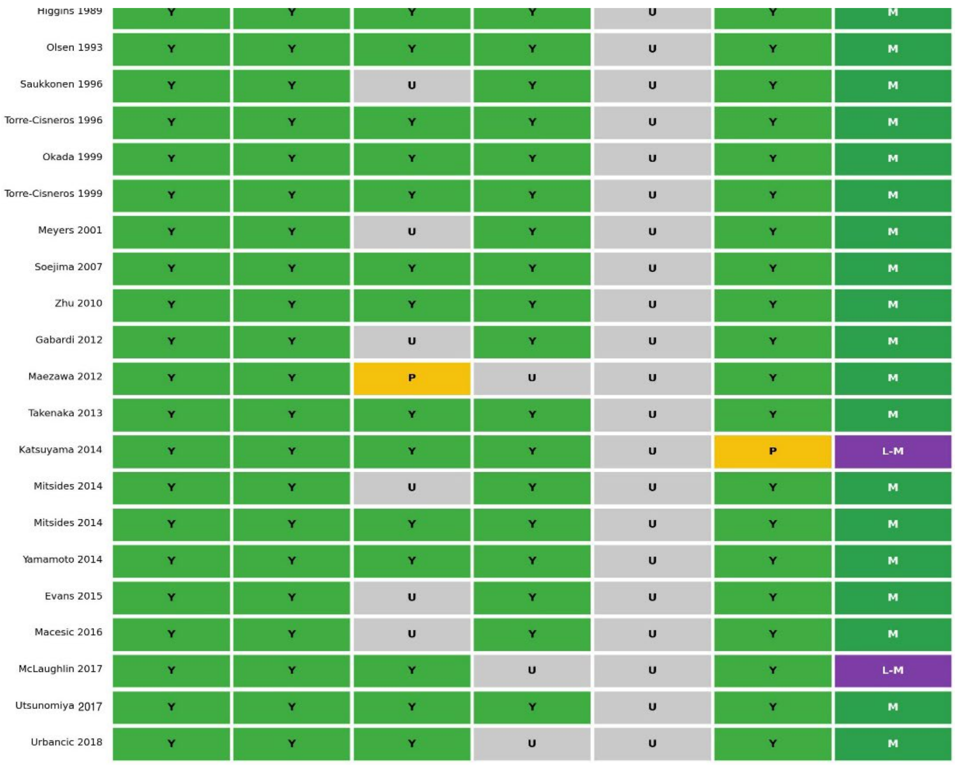

Supplementary Figure S4. Adapted JBI traffic-light plot for single-arm/descriptive studies, part 1. Conference abstracts are excluded from the main evidence set.

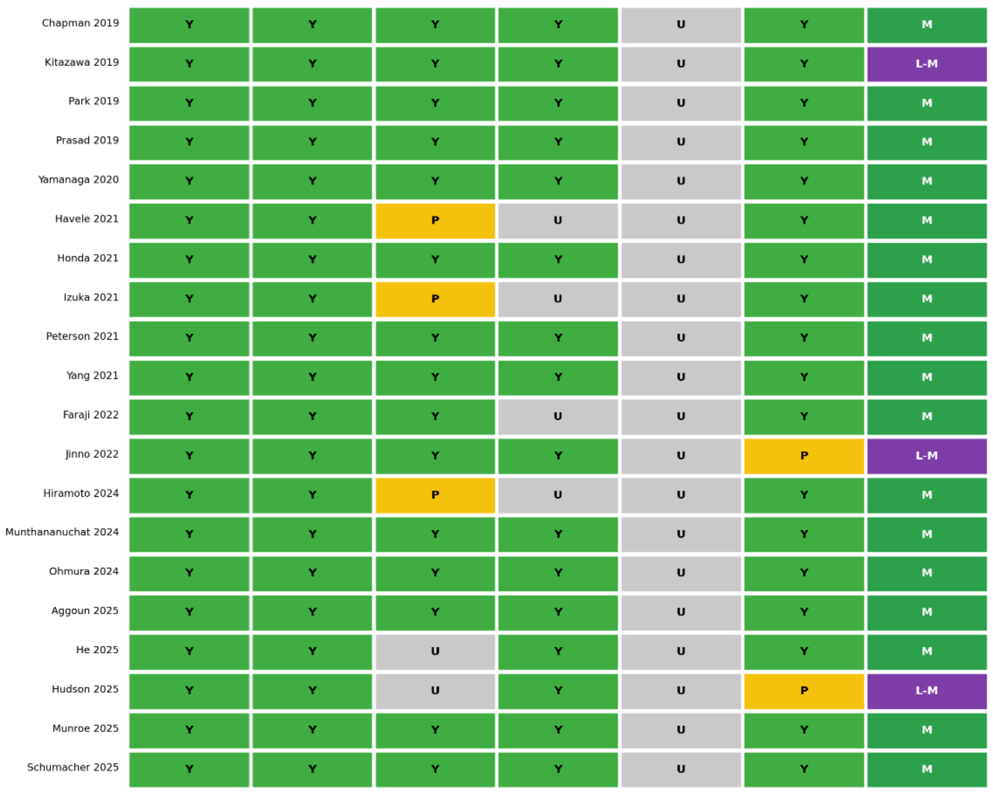

Supplementary Figure S5. Adapted JBI traffic-light plot for single-arm/descriptive studies, part 2. Schumacher 2025 is included as a full peer-reviewed Route C report.

Abbreviations used in domain profiles: RoB 2: D1 randomization, D2 deviations, D3 missing data, D4 outcome measurement, D5 selective reporting; L = low risk, SC = some concerns, H = high risk. ROBINS-I: D1 confounding, D2 selection, D3 intervention classification, D4 deviations, D5 missing data, D6 outcome measurement, D7 selective reporting; L = low risk of bias, M = moderate risk of bias, S = serious risk of bias, C = critical risk of bias, NI = no information. No studies were judged as critical risk of bias or no information in this assessment. Adapted JBI: Q1 clear inclusion, Q2 exposure/intervention measured reliably, Q3 outcome measured reliably, Q4 follow-up/missing data, Q5 consecutive/representative participants, Q6 denominator/events; Y = yes, P = partly, U = unclear/not reported, N = no.

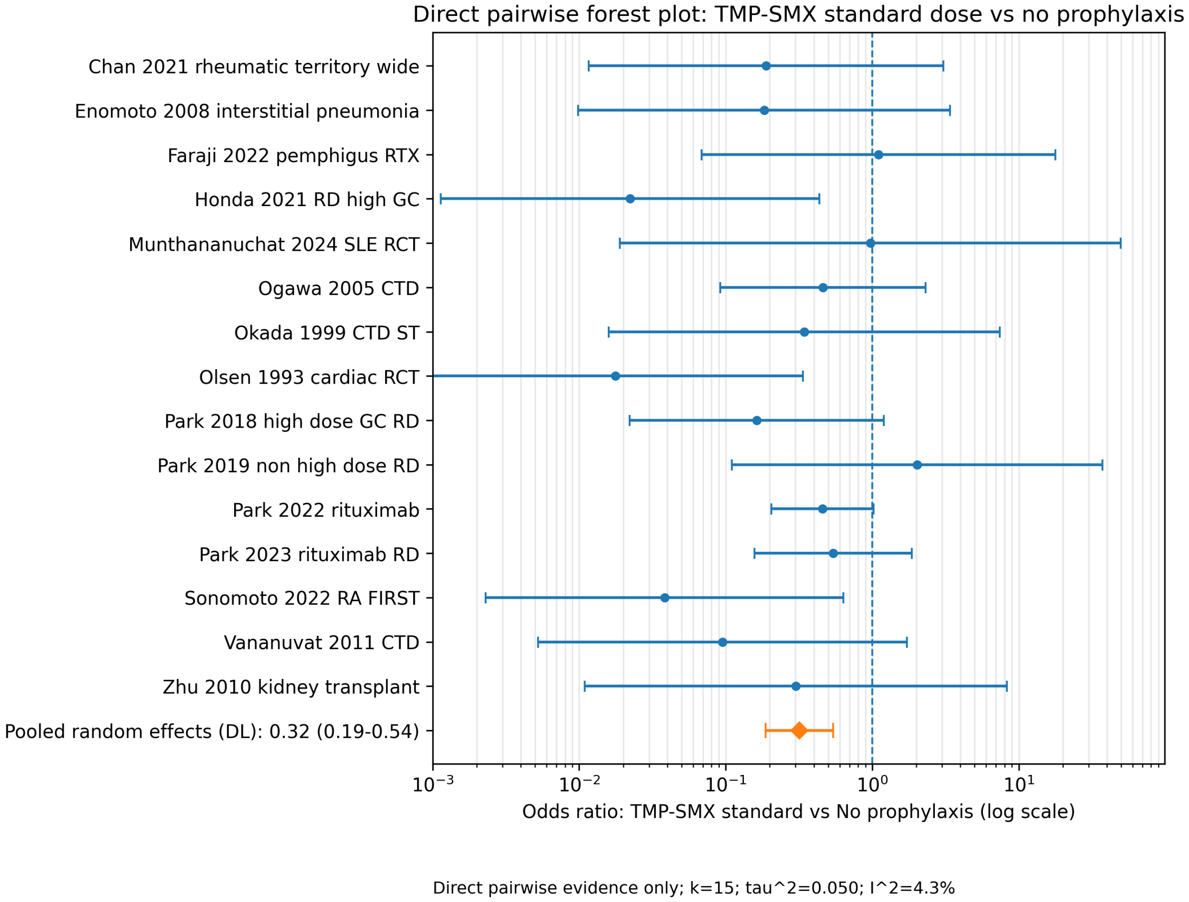

Supplementary Figure S6. Direct pairwise forest plot of TMP-SMX standard dose versus no prophylaxis/observation/placebo for PCP incidence. Odds ratios below 1 favor TMP-SMX standard-dose prophylaxis.

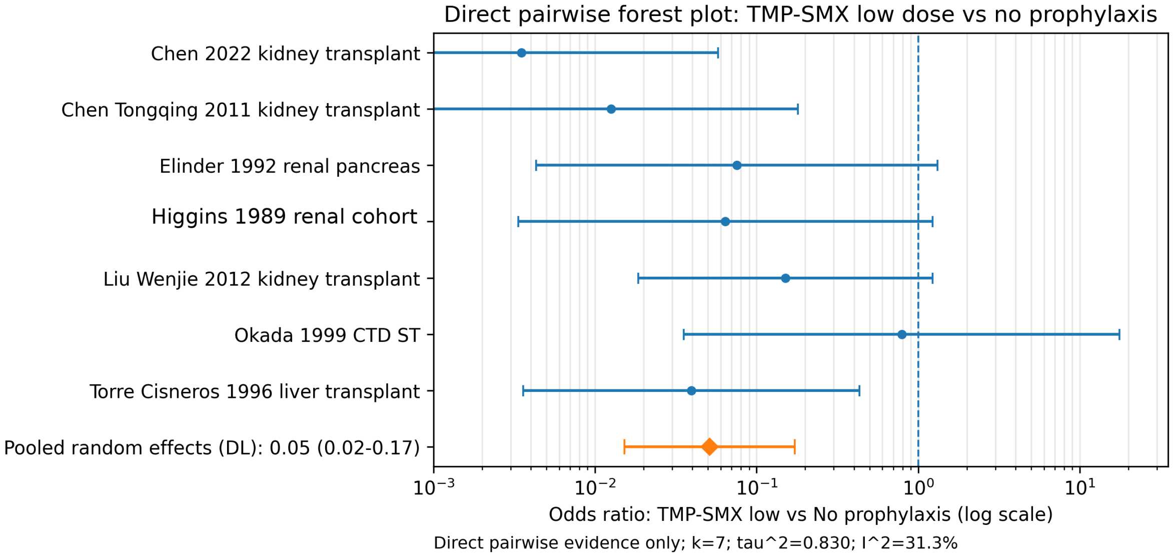

Supplementary Figure S7. Direct pairwise forest plot of TMP-SMX low dose versus no prophylaxis/observation/placebo for PCP incidence. Odds ratios below 1 favor low-dose TMP-SMX prophylaxis. The estimate should be interpreted in the context of sparse events and transitivity concerns.

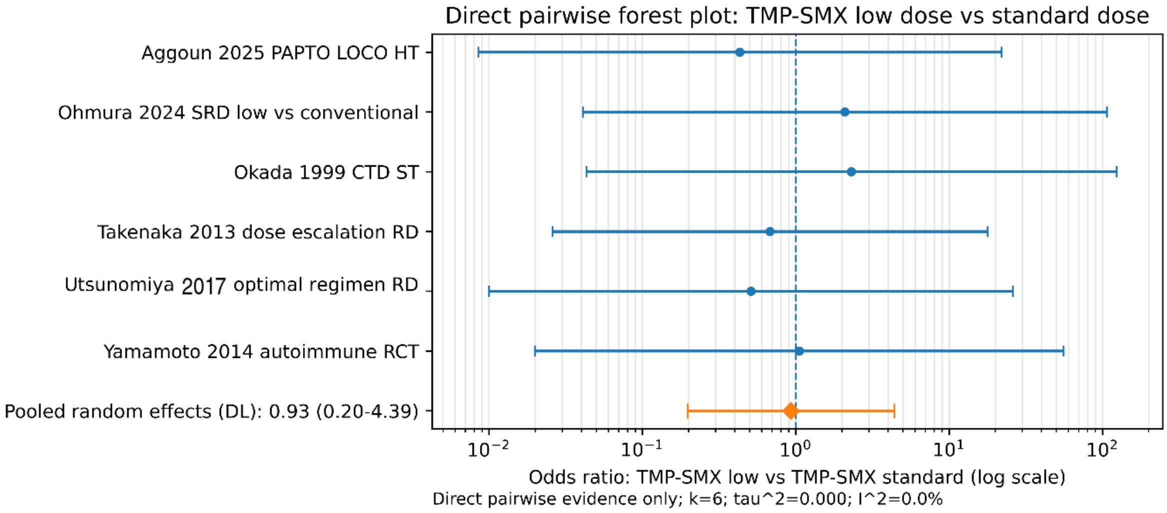

Supplementary Figure S8. Direct pairwise forest plot of TMP-SMX low dose versus TMP-SMX standard dose for PCP incidence. Direct evidence did not demonstrate superiority of low-dose over standard-dose TMP-SMX; the network estimate favoring low-dose should therefore be interpreted cautiously.

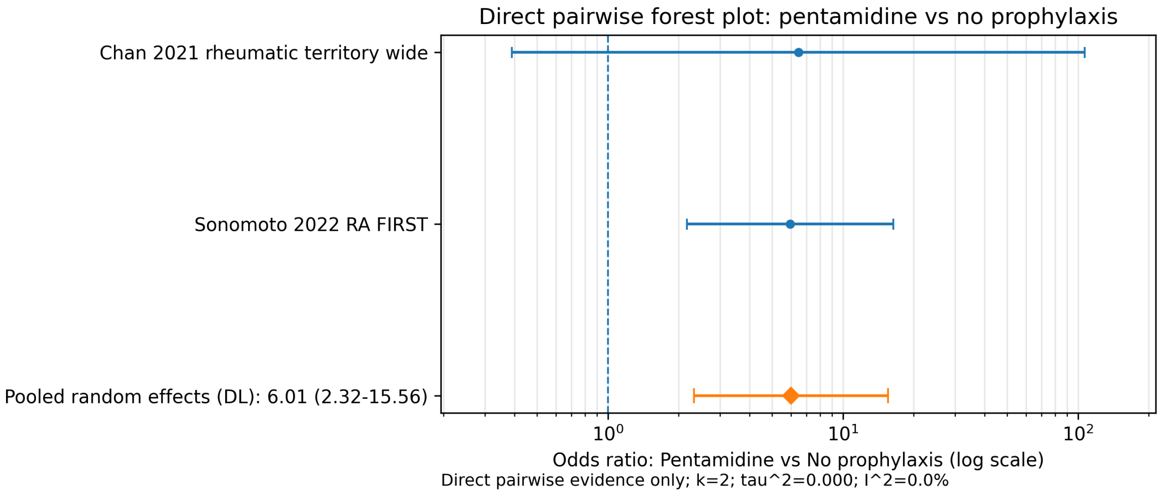

Supplementary Figure S9. Direct pairwise forest plot of pentamidine versus no prophylaxis/observation/placebo for PCP incidence. Evidence was sparse and potentially affected by channeling or indication bias; the estimate should not be interpreted as causal evidence of harm or inferiority.

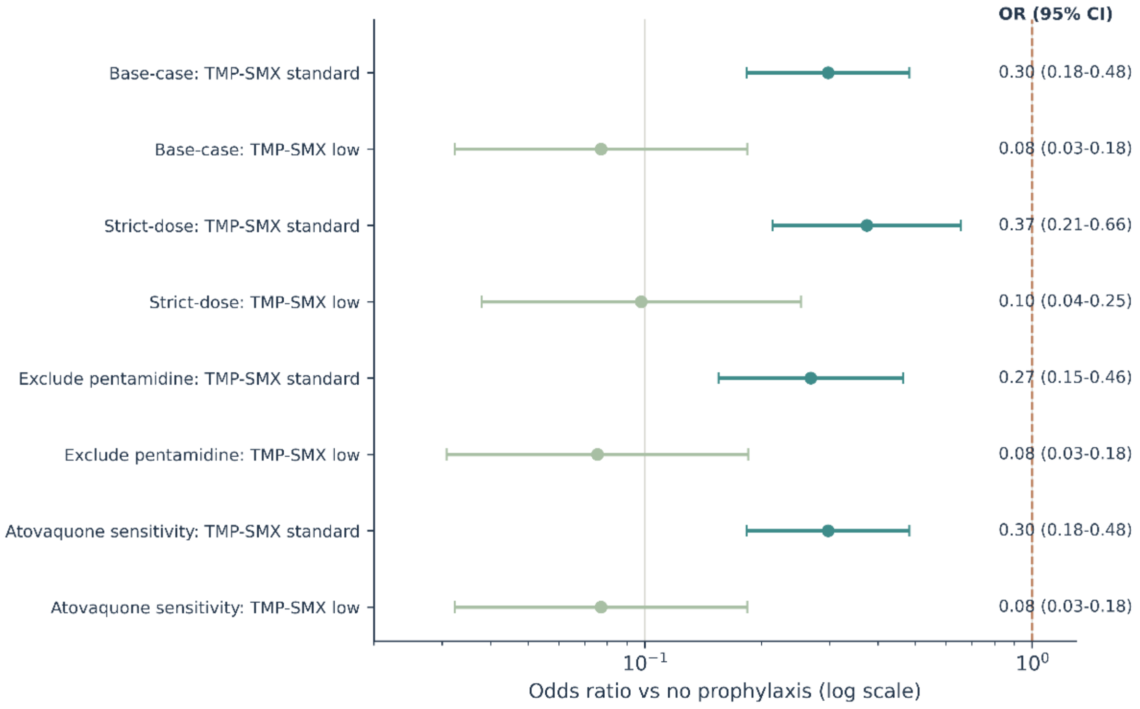

Supplementary Figure S10. Sensitivity analysis of the primary comparative effectiveness network meta-analysis. Forest plot of sensitivity network meta-analysis estimates for PCP incidence under alternative node or study-inclusion assumptions.

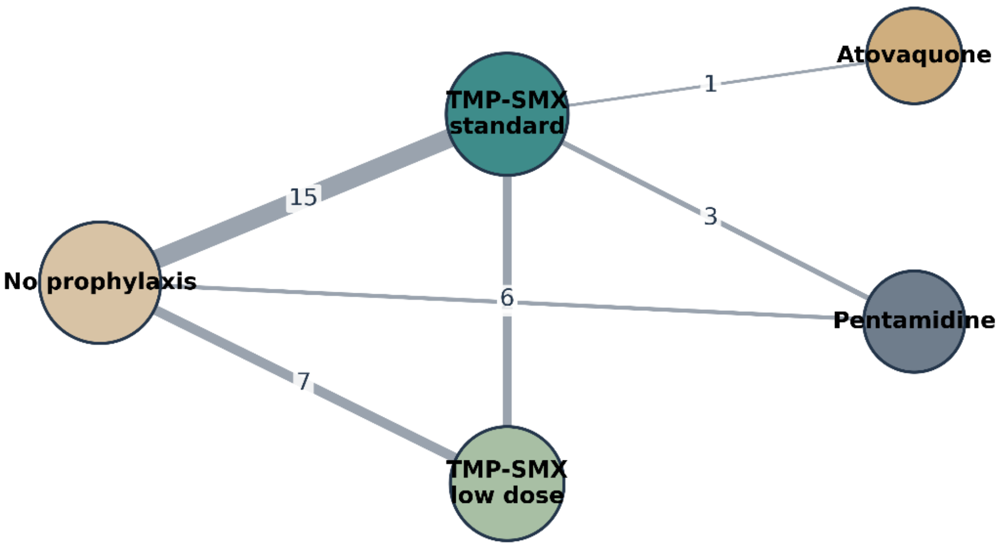

Supplementary Figure S11. Atovaquone sensitivity network for the primary comparative effectiveness analysis. Network geometry after including atovaquone as a sensitivity node. Atovaquone was not included in the base-case effectiveness network because available data largely reflected second-line use after TMP-SMX intolerance or discontinuation.

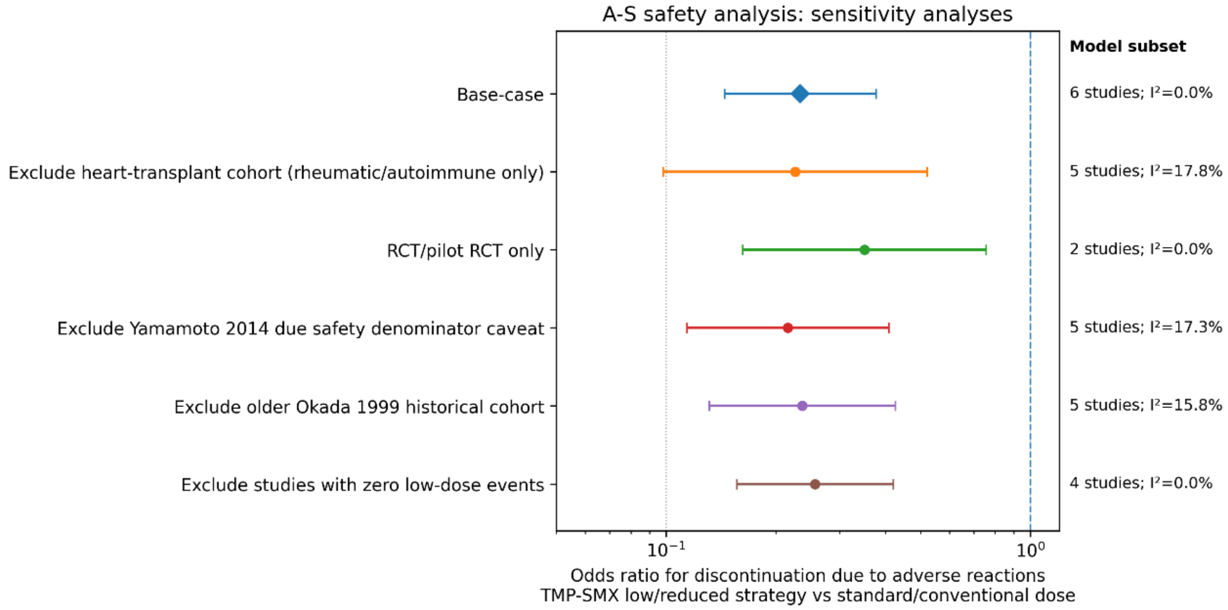

Supplementary Figure S12. Sensitivity analyses for the active-regimen safety analysis. Forest plot of sensitivity analyses comparing discontinuation due to adverse events between low-dose and standard-dose TMP-SMX.

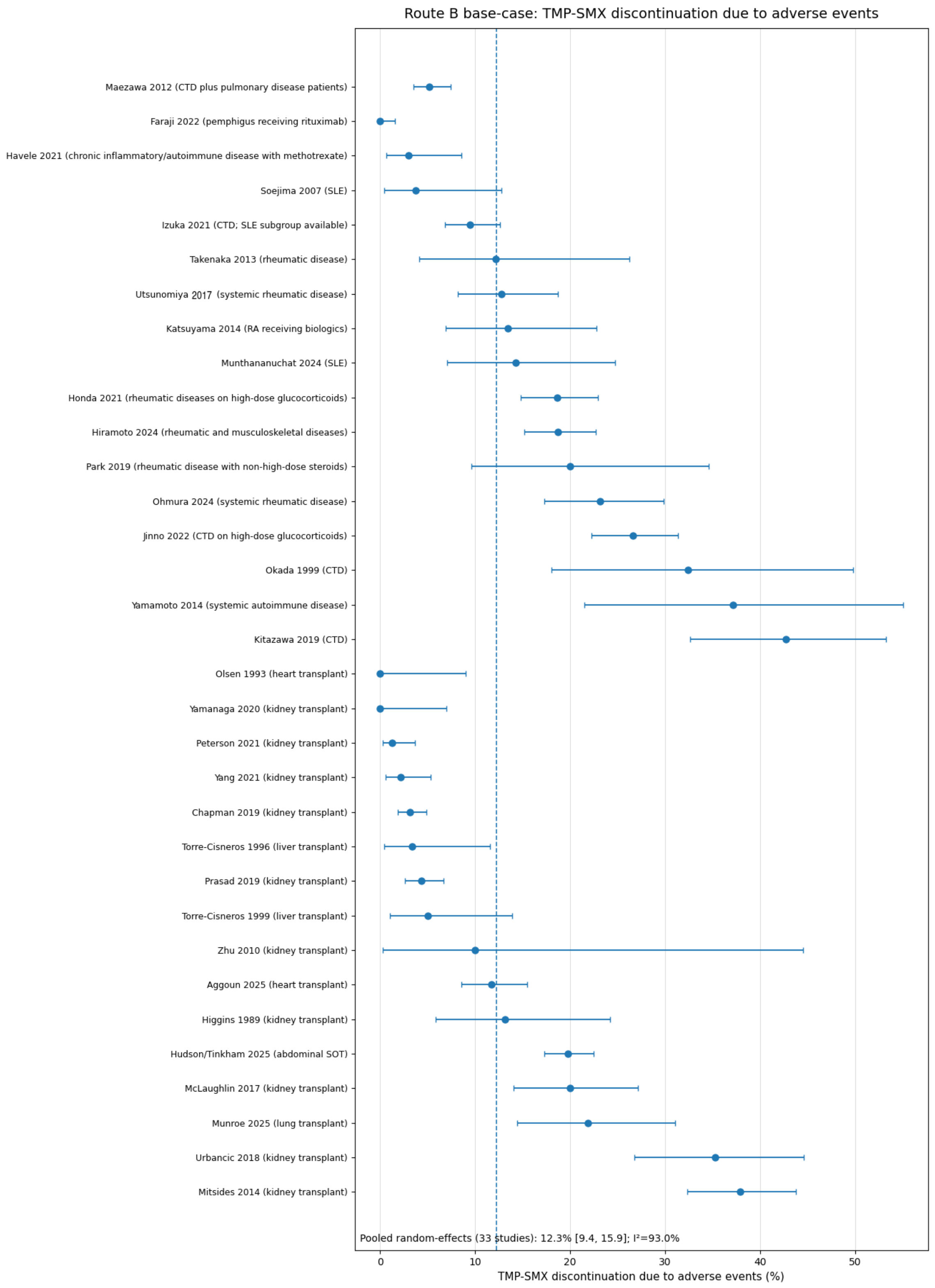
Route B base-case: TMP-SMX discontinuation due to adverse events
Maezawa 2012 (CTD plus pulmonary disease patients)
Faraji 2022 (pemphigus receiving rituximab)
Havele 2021 (chronic inflammatory/autoimmune disease with methotrexate)
Soejima 2007 (SLE)
Izuka 2021 (CTD; SLE subgroup available)
Takenaka 2013 (rheumatic disease)
Utsunomiya 2017 (systemic rheumatic disease)
Katsuyama 2014 (RA receiving biologics)
Munthananuchat 2024 (SLE)
Honda 2021 (rheumatic diseases on high-dose glucocorticoids)
Hiramoto 2024 (rheumatic and musculoskeletal diseases)
Park 2019 (rheumatic disease with non-high-dose steroids)
Ohmura 2024 (systemic rheumatic disease)
Jinno 2022 (CTD on high-dose glucocorticoids)
Okada 1999 (CTD)
Yamamoto 2014 (systemic autoimmune disease)
Kitazawa 2019 (CTD)
Olsen 1993 (heart transplant)
Yamanaga 2020 (kidney transplant)
Peterson 2021 (kidney transplant)
Yang 2021 (kidney transplant)
Chapman 2019 (kidney transplant)
Torre-Cisneros 1996 (liver transplant)
Prasad 2019 (kidney transplant)
Torre-Cisneros 1999 (liver transplant)
Zhu 2010 (kidney transplant)
Aggoun 2025 (heart transplant)
Higgins 1989 (kidney transplant)
Hudson/Tinkham 2025 (abdominal SOT)
McLaughlin 2017 (kidney transplant)
Munroe 2025 (lung transplant)
Urbancic 2018 (kidney transplant)
Mitsides 2014 (kidney transplant)
Pooled random-effects (33 studies): 12.3% [9.4, 15.9]; I²=93.0%
0
10
20
30
40
50
TMP-SMX discontinuation due to adverse events (%)

Supplementary Figure S13. Study-level forest plot of TMP-SMX discontinuation due to adverse events across 33 eligible independent studies/cohorts. The primary analysis excludes conference abstracts.

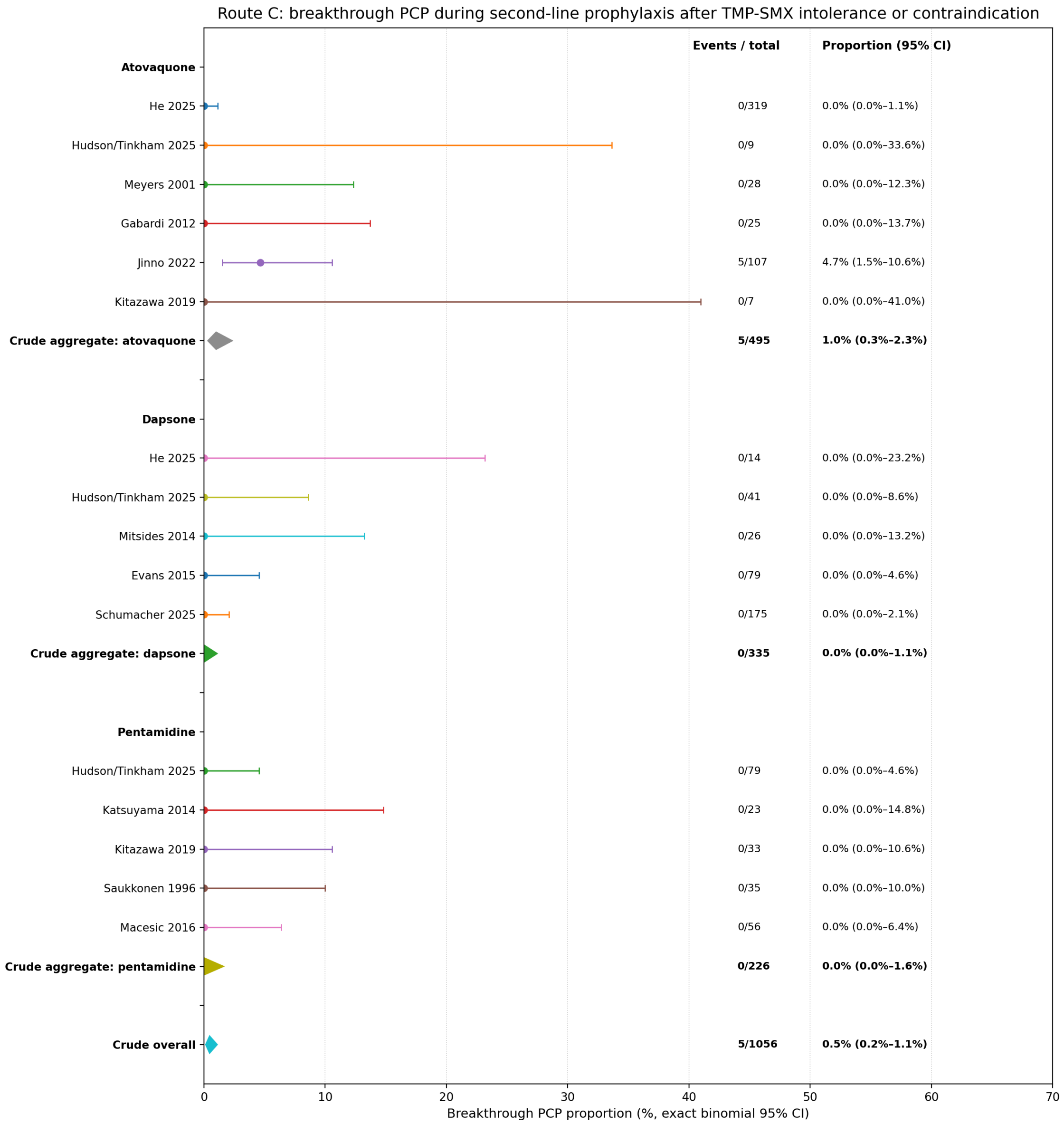

Supplementary Figure S14. Crude breakthrough PCP proportions during second-line prophylaxis after TMP-SMX intolerance, contraindication, or discontinuation. The main analysis includes 12 reports and 16 arms. 'Crude aggregate' and 'Crude overall' are simple event/denominator summaries with exact binomial 95% CIs, not meta-analytic pooled effects. Conference abstracts are excluded.

Supplementary Table

Section S1. Search strategy and study selection

Supplementary Table S1. Full search strategies by database and registry.

Searches were conducted on 18 May 2026. For reproducibility, search strings are reported in the syntax of the original advanced-search interface into which they were copied, rather than being converted into another platform-specific syntax. Record counts correspond to the original search date and may differ if the searches are rerun after database indexing updates.

S1.1. PubMed/MEDLINE

**Interface / search mode**	PubMed Advanced Search
**Date searched**	18 May 2026
**Records retrieved**	714
**Fields / limits**	MeSH, Subheading, and Title/Abstract fields. The animal-only exclusion was applied using NOT (animals[mh] NOT humans[mh]). No date or language restriction was entered in the search string.

Search string as entered:

```
(
  "Pneumonia, Pneumocystis"[Mesh] OR
  "Pneumocystis jirovecii"[Mesh] OR
  "Pneumocystis carinii"[Mesh] OR
  pneumocystis[tiab] OR
  "Pneumocystis jirovecii"[tiab] OR
  "Pneumocystis jiroveci"[tiab] OR
  "Pneumocystis carinii"[tiab] OR
  "pneumocystis pneumonia"[tiab] OR
  "pneumocystis pneumonitis"[tiab] OR
  pneumocystosis[tiab] OR
  PCP[tiab] OR
  PJP[tiab]
)
AND
(
  "Chemoprevention"[Mesh] OR
  "prevention and control"[Subheading] OR
  prophylaxis[tiab] OR
  prophylactic[tiab] OR
  prevention[tiab] OR
  chemoprophylaxis[tiab] OR
  "primary prophylaxis"[tiab] OR
  "secondary prophylaxis"[tiab]
)
AND
(
  "Trimethoprim, Sulfamethoxazole Drug Combination"[Mesh] OR
  "trimethoprim-sulfamethoxazole"[tiab] OR
  "trimethoprim sulfamethoxazole"[tiab] OR
  "TMP-SMX"[tiab] OR
  "TMP SMX"[tiab] OR
  "TMP-SMZ"[tiab] OR
  "TMP SMZ"[tiab] OR
```

```
  co-trimoxazole[tiab] OR
  cotrimoxazole[tiab] OR
  sulfamethoxazole[tiab] OR
  Bactrim[tiab] OR
  "Atovaquone"[Mesh] OR
  atovaquone[tiab] OR
  "Dapsone"[Mesh] OR
  dapsone[tiab] OR
  "Pentamidine"[Mesh] OR
  pentamidine[tiab]
)
AND
(
  "Rheumatic Diseases"[Mesh] OR
  "Autoimmune Diseases"[Mesh] OR
  "Connective Tissue Diseases"[Mesh] OR
  "Vasculitis"[Mesh] OR
  "Lupus Erythematosus, Systemic"[Mesh] OR
  "Arthritis, Rheumatoid"[Mesh] OR
  "Myositis"[Mesh] OR
  "Scleroderma, Systemic"[Mesh] OR
  "Organ Transplantation"[Mesh] OR
  "Transplant Recipients"[Mesh] OR
  "Immunocompromised Host"[Mesh] OR
  "Immunosuppression Therapy"[Mesh] OR
  rheumatic[tiab] OR
  autoimmune[tiab] OR
  "connective tissue"[tiab] OR
  vasculitis[tiab] OR
  lupus[tiab] OR
  SLE[tiab] OR
  "rheumatoid arthritis"[tiab] OR
  myositis[tiab] OR
  dermatomyositis[tiab] OR
```

```
  polymyositis[tiab] OR
  scleroderma[tiab] OR
  "systemic sclerosis"[tiab] OR
  Sjogren[tiab] OR
  "Sjögren"[tiab] OR
  ANCA[tiab] OR
  granulomatosis[tiab] OR
  transplant[tiab] OR
  transplantation[tiab] OR
  immunosuppressed[tiab] OR
  immunocompromised[tiab] OR
  immunosuppression[tiab] OR
  glucocorticoid[tiab] OR
  corticosteroid[tiab] OR
  steroid[tiab]
)
NOT
(
  animals[mh] NOT humans[mh]
)
```

S1.2. Embase

**Interface / search mode**	Embase advanced search interface (Embase.com syntax as entered; not Ovid syntax)
**Date searched**	18 May 2026
**Records retrieved**	2,124
**Fields / limits**	Emtree terms were exploded using /exp; free-text terms were searched in title, abstract, and keyword fields (:ti,ab,kw). The human limit was applied using AND [humans]/lim.

Search string as entered:

```
(
  'pneumocystis pneumonia'/exp OR
  'pneumocystis jirovecii'/exp OR
  pneumocystis:ti,ab,kw OR
  'pneumocystis jirovecii':ti,ab,kw OR
  'pneumocystis jiroveci':ti,ab,kw OR
  'pneumocystis carinii':ti,ab,kw OR
  'pneumocystis pneumonia':ti,ab,kw OR
  pneumocystosis:ti,ab,kw OR
  PCP:ti,ab,kw OR
  PJP:ti,ab,kw
)
AND
(
  'chemoprophylaxis'/exp OR
  prophylaxis:ti,ab,kw OR
  prophylactic:ti,ab,kw OR
  prevention:ti,ab,kw OR
  chemoprophylaxis:ti,ab,kw OR
  'primary prophylaxis':ti,ab,kw OR
  'secondary prophylaxis':ti,ab,kw
)
AND
(
  'trimethoprim sulfamethoxazole'/exp OR
  'trimethoprim-sulfamethoxazole':ti,ab,kw OR
  'trimethoprim sulfamethoxazole':ti,ab,kw OR
  'TMP-SMX':ti,ab,kw OR
  'TMP SMX':ti,ab,kw OR
  'TMP-SMZ':ti,ab,kw OR
  'TMP SMZ':ti,ab,kw OR
  co-trimoxazole:ti,ab,kw OR
  cotrimoxazole:ti,ab,kw OR
  sulfamethoxazole:ti,ab,kw OR
```

```
  bactrim:ti,ab,kw OR
  'atovaquone'/exp OR
  atovaquone:ti,ab,kw OR
  'dapsone'/exp OR
  dapsone:ti,ab,kw OR
  'pentamidine'/exp OR
  pentamidine:ti,ab,kw
)
AND
(
  'rheumatic disease'/exp OR
  'autoimmune disease'/exp OR
  'organ transplantation'/exp OR
  'transplant recipient'/exp OR
  'immunocompromised patient'/exp OR
  'immunosuppressive treatment'/exp OR
  rheumatic:ti,ab,kw OR
  autoimmune:ti,ab,kw OR
  vasculitis:ti,ab,kw OR
  lupus:ti,ab,kw OR
  'rheumatoid arthritis':ti,ab,kw OR
  myositis:ti,ab,kw OR
  dermatomyositis:ti,ab,kw OR
  scleroderma:ti,ab,kw OR
  transplant:ti,ab,kw OR
  transplantation:ti,ab,kw OR
  immunosuppressed:ti,ab,kw OR
  immunocompromised:ti,ab,kw OR
  immunosuppression:ti,ab,kw OR
  glucocorticoid:ti,ab,kw OR
  corticosteroid:ti,ab,kw OR
  steroid:ti,ab,kw
)
AND [humans]/lim
```

S1.3. Web of Science Core Collection

**Interface / search mode**	Web of Science Advanced Search
**Date searched**	18 May 2026
**Records retrieved**	1,908
**Fields / limits**	Topic search (TS). No additional date or language restriction was entered in the search string.

Search string as entered:

```
TS=(
  pneumocystis OR
  "Pneumocystis jirovecii" OR
  "Pneumocystis jiroveci" OR
  "Pneumocystis carinii" OR
  "pneumocystis pneumonia" OR
  pneumocystosis OR
  PCP OR
  PJP
)
AND
TS=(
  prophylaxis OR
  prophylactic OR
  prevention OR
  chemoprophylaxis OR
  "primary prophylaxis" OR
  "secondary prophylaxis" OR
  "trimethoprim-sulfamethoxazole" OR
  "trimethoprim sulfamethoxazole" OR
  "TMP-SMX" OR
  "TMP-SMZ" OR
  cotrimoxazole OR
  "co-trimoxazole" OR
  atovaquone OR
  dapsone OR
  pentamidine
)
AND
TS=(
  rheumatic OR
  autoimmune OR
  "connective tissue" OR
  vasculitis OR
```

```
  lupus OR
  SLE OR
  "rheumatoid arthritis" OR
  myositis OR
  dermatomyositis OR
  polymyositis OR
  scleroderma OR
  "systemic sclerosis" OR
  Sjogren OR
  ANCA OR
  granulomatosis OR
  transplant* OR
  immunosuppressed OR
  immunocompromised OR
  immunosuppression OR
  glucocorticoid* OR
  corticosteroid* OR
  steroid*
)
```

S1.4. Cochrane Central Register of Controlled Trials / CENTRAL

**Interface / search mode**	Cochrane Library advanced search
**Date searched**	18 May 2026
**Records retrieved**	271 trials
**Fields / limits**	Title, Abstract, and Keyword fields.

Search string as entered:

```
Title Abstract Keyword
(
  pneumocystis OR
  "Pneumocystis jirovecii" OR
  "Pneumocystis jiroveci" OR
  "Pneumocystis carinii" OR
  "pneumocystis pneumonia" OR
  "pneumocystis pneumonitis" OR
  pneumocystosis OR
  PCP OR
  PJP
)
AND
(
  prophylaxis OR
  prophylactic OR
  prevention OR
  chemoprophylaxis OR
  "primary prophylaxis" OR
  "secondary prophylaxis"
)
AND
(
  "trimethoprim-sulfamethoxazole" OR
  "trimethoprim sulfamethoxazole" OR
  "TMP-SMX" OR
  "TMP SMX" OR
  "TMP-SMZ" OR
  "TMP SMZ" OR
  co-trimoxazole OR
  cotrimoxazole OR
  sulfamethoxazole OR
  bactrim OR
  atovaquone OR
```

dapsone OR<br>pentamidine<br>)

S1.5. CNKI / China National Knowledge Infrastructure

**Interface / search mode**	CNKI Advanced Search
**Date searched**	18 May 2026
**Records retrieved**	132
**Fields / limits**	The search was executed in Chinese in the CNKI advanced-search box; an English translation is reported below. Multiple Chinese synonyms may translate to the same English term. When entering the operators * (AND), + (OR), and - (NOT), one byte should be left blank before and after each operator.

English translation of the Chinese-language search string used:

((Pneumocystis + Pneumocystis + Pneumocystis jirovecii + Pneumocystis carinii + Pneumocystis pneumonia + Pneumocystis pneumonia) AND<br>(prevention + prophylactic + primary prophylaxis + secondary prophylaxis + chemoprophylaxis)<br>Equivalent Boolean logic for reporting: (Pneumocystis OR Pneumocystis OR Pneumocystis jirovecii OR Pneumocystis carinii OR Pneumocystis pneumonia OR Pneumocystis pneumonia) AND (prevention OR prophylactic OR primary prophylaxis OR secondary prophylaxis OR chemoprophylaxis)

S1.6. Wanfang Data

**Interface / search mode**	Wanfang enhanced retrieval
**Date searched**	18 May 2026
**Records retrieved**	152
**Fields / limits**	Enhanced retrieval. Chinese and English disease terms were combined with prevention terms. An English translation is reported below; multiple Chinese synonyms may translate to the same English term.

English translation of the Chinese-language search string used:

(Pneumocystis OR Pneumocystis OR Pneumocystis jirovecii OR Pneumocystis carinii OR Pneumocystis pneumonia OR Pneumocystis pneumonia OR Pneumocystis)<br>AND<br>(prevention OR prophylactic OR primary prophylaxis OR secondary prophylaxis OR chemoprophylaxis)

S1.7. ClinicalTrials.gov

**Interface / search mode**	ClinicalTrials.gov advanced search
**Date searched**	18 May 2026
**Records retrieved**	60
**Fields / limits**	Condition/disease and Intervention/treatment fields. Other fields were not restricted as recorded.

Search string as entered:

Condition/disease:Pneumocystis pneumonia OR Pneumocystis jirovecii pneumonia OR PCP OR PJP
Intervention/treatment:trimethoprim sulfamethoxazole OR TMP-SMX OR cotrimoxazole OR atovaquone OR dapsone OR pentamidine

S1.8. WHO International Clinical Trials Registry Platform / WHO ICTRP

**Interface / search mode**	WHO ICTRP Advanced Search
**Date searched**	18 May 2026
**Records retrieved**	29 trials
**Fields / limits**	Title / Condition field. The intervention field was not restricted in the recorded search.

Search string as entered:

Title / Condition:pneumocystis OR "Pneumocystis pneumonia" OR PCP OR PJP

Abbreviations: CENTRAL, Cochrane Central Register of Controlled Trials; CNKI, China National Knowledge Infrastructure; ICTRP, International Clinical Trials Registry Platform; MeSH, Medical Subject Headings; PCP, *Pneumocystis jirovecii* pneumonia; PJP, *Pneumocystis jirovecii* pneumonia; TMP-SMX, trimethoprim-sulfamethoxazole; TS, Topic Search.

Supplementary Table S2. Reports excluded after eligibility assessment, with reasons (n=134).

Primary exclusion reason category	Reports, n
Ineligible study design / non-original report / no final study results	43
Wrong intervention/comparison or wrong outcome focus	28
Duplicate / overlapping cohort or preliminary report	22
Insufficient extractable data for prespecified analyses	23
Ineligible or non-separable population	2
Conference abstract only; no corresponding full-length peer-reviewed report identified	16
Total	134

Panel A. Detailed record-level exclusions after assessment

First author or registry, year	Title or short citation	Source/database	Primary exclusion reason category	Detailed reason	Potential route considered
Spieker, C. 1991	Pentamidine inhalation - An efficient prophylaxis against Pneumocystis pneumonia in renal transplant patients treated with monoclonal antibodies	Embase	Duplicate / overlapping cohort or preliminary report	This record was conservatively excluded as a likely overlapping or preliminary report of the Barenbrock 1992 cohort to avoid double counting.	None
Kramer, M.R. 1992	Trimethoprim-sulfamethoxazole prophylaxis for Pneumocystis carinii infections in heart-lung and lung transplantation - How effective and for how long?	PubMed/MEDLINE; Embase; Web of Science	Ineligible or non-separable population	The abstract reports PCP events, but the total number of participants in each arm is unclear.	A-E
Barenbrock, M. 1992	[Pentamidine inhalation in prevention of pneumocystis carinii pneumonia in treatment of rejection with monoclonal antibody Orthoclone (OKT-3)].	PubMed/MEDLINE; Web of Science	Duplicate / overlapping cohort or preliminary report	Pentamidine was compared with historical no prophylaxis, and PCP event counts were reported; however, this report should be excluded to avoid duplicate counting.	A-E; C
Olsen, S.L. 1993	PREVENTION OFPneumocystis cariniiPNEUMONIA IN CARDIAC TRANSPLANT RECIPIENTS BY TRIMETHOPRIM SULFAMETHOXAZOLE	Wanfang	Duplicate / overlapping cohort or preliminary report	Duplicate report of the full Olsen 1993 study; not counted separately.	None
Pedagogos, E 1994	Comparison of oral trimethoprim-sulphamethoxazole (TM-SMX) and inhaled pentamidine (P) for prevention of pneumocystis pneumonia (PCP) after renal transplantation	CENTRAL	Conference abstract only; no corresponding full-length peer-reviewed report identified	Conference abstract only; no corresponding full-length peer-reviewed report was identified, and the available information was insufficient for reliable data verification and risk-of-bias assessment.	A-E; A-S; C
Pedagogos, E. 1994	ORAL TRIMETHOPRIM-SULFAMETHOXAZOLE (TM-SMX) VS INHALED PENTAMIDINE (P) FOR PREVENTION OF PNEUMOCYSTIS PNEUMONIA (PCP) AFTER RENAL-TRANSPLANTATION	Web of Science	Duplicate / overlapping cohort or preliminary report	Possibly the same conference abstract/study as the Pedagogos 1994 TSANZ abstract.	None
Vernovsky, I. 2000	Pneumocystis carinii pneumonia prophylaxis in patients with rheumatic diseases undergoing immunosuppressive therapy - Prevalence and associated features	PubMed/MEDLINE; Embase; Web of Science	Ineligible study design / non-original report / no final study results	The abstract appears to be a review or recommendation-oriented article discussing PCP risk and prophylaxis in rheumatic diseases, without original extractable clinical data.	None
Clinical trial registry, 2009	A prospective study of Trimethoprime-sulfamethoxazole(TMP/SMX) for prevention of Pneumocystis pneumonia(PCP) in patients under immunosuppressive therapy	CENTRAL	Duplicate / overlapping cohort or preliminary report	This was a trial registry record and did not itself contain outcome data. It likely corresponds to the full Yamamoto 2014 trial report and should be merged with that publication; if not corresponding, it would still be excluded because no events/total data were available.	None
Gabardi, S. 2010	Evaluation of atovaquone versus sulfamethoxazoletrimethorpim as Pneumocystis jiroveci pneumonia prophylaxis following renal transplantation	Embase	Duplicate / overlapping cohort or preliminary report	The conference abstract was highly consistent with the full Gabardi 2012 article, including a sample size of 160 TMP-SMX recipients and 25 atovaquone recipients; it was not counted separately.	C
Anand, S. 2011	*Pneumocystis jirovecii* pneumonia is rare in renal transplant recipients receiving only one month of prophylaxis.	PubMed/MEDLINE; Embase; Web of Science	Wrong intervention/comparison or wrong outcome focus	Kidney/kidney-pancreas transplant population; PCP was reported after one month of TMP-SMX prophylaxis (4/1352), but there was no comparator group and no TMP-SMX discontinuation data. It was therefore not eligible for the main NMA or route B.	A-E; B
Mizushina, K. 2014	Possible preventive effect of salazosulfapyridine on the development of pneumocystis pneumonia in patients with rheumatoid arthritis	Embase; CENTRAL	Conference abstract only; no corresponding full-length peer-reviewed report identified	Conference abstract only; no corresponding full-length peer-reviewed report was identified, and the available information was insufficient for reliable data verification and risk-of-bias assessment.	A-S

First author or registry, year	Title or short citation	Source/database	Primary exclusion reason category	Detailed reason	Potential route considered
Fan, Z. 2015	Use of compound sulfamethoxazole tablets for prevention of Pneumocystis carinii pneumonia after kidney transplantation	Wanfang	Conference abstract only; no corresponding full-length peer-reviewed report identified	Conference abstract only; no corresponding full-length peer-reviewed report was identified, and the available information was insufficient for reliable data verification and risk-of-bias assessment.	A-E; B
Park, J.W. 2017	Primary prophylaxis for pneumocystis pneumonia in patients with rheumatic disease and treated with prolonged, high-dose steroid: A retrospective cohort study with 12-year observation	Embase; CENTRAL; Web of Science	Duplicate / overlapping cohort or preliminary report	Same study/cohort as the full Park 2018 report; the full report was retained and this abstract was not counted separately.	None
Clinical trial registry, 2018	Optimal regimens of sulfamethoxazole-trimethoprim for prophylaxis of pneumocystis pneumonia in patients with systemic rheumatic diseases: multi-center randomized open label trial	CENTRAL	Duplicate / overlapping cohort or preliminary report	Trial registry record corresponding to the Abe 2019 protocol article. No outcome data were available, so it was not included separately.	None
Abe, Y. 2019	Conventional-dose Versus Half-dose Sulfamethoxazole-trimethoprim for the Prophylaxis of Pneumocystis Pneumonia in Patients with Systemic Rheumatic Disease: A Non-blind, Randomized Controlled Trial.	PubMed/MEDLINE; Embase; CENTRAL; Web of Science	Ineligible study design / non-original report / no final study results	This was a non-blinded randomized trial protocol/registry article reporting planned primary and secondary outcomes only; no events/total data were available.	None
Pan, J. 2020	Single-Center Retrospective Analysis of Prophylaxis and Treatment of Pneumocystis carinii Pneumonia in Patients with Renal Dysfunction After Renal Transplantation.	PubMed/MEDLINE; Embase; Web of Science	Ineligible study design / non-original report / no final study results	This study included 68 kidney transplant recipients with established PCP and compared clinical features/outcomes according to renal dysfunction status. It was not a prophylaxis study and did not provide prophylaxis-arm denominators.	None
Lipman, K.B.W.G. 2021	Pneumocystis jiroveci Pneumonia prophylaxis in patients treated with mycophenolate mofetil for interstitial lung disease	Embase; Web of Science	Conference abstract only; no corresponding full-length peer-reviewed report identified	Conference abstract only; no corresponding full-length peer-reviewed report was identified, and the available information was insufficient for reliable data verification and risk-of-bias assessment.	B
Clinical trial registry, 2021	Prophylaxis of pneumocystis jiroveci infection after renal transplantation with very low dose of compound sulfamethoxazole dispersible tablets: a prospective randomized controlled study	CENTRAL	Ineligible study design / non-original report / no final study results	Trial registry record with only intervention and outcome plans. No results were available; if a full results article is later identified, it should be reassessed.	None
Clinical trial registry, 2022	Daily single-strength versus thrice-weekly double-strength trimethoprim/sulfamethoxazole for *Pneumocystis jirovecii* prophylaxis in solid organ transplant recipients: a randomized control trial	CENTRAL	Duplicate / overlapping cohort or preliminary report	This registry record was treated as a duplicate/preliminary record because a full 2025 report was available and was assessed preferentially. The registry was not counted separately, and no independent outcome data were extracted from it.	A-E; A-S; B
Nakaya, I. 2023	Chemoprophylaxis against *Pneumocystis jirovecii* pneumonia in Japanese patients with ANCA-associated vasculitis: An observational study	Embase; Web of Science	Insufficient extractable data for prespecified analyses	The AAV population and topic were relevant, but chemoprophylaxis exposure varied dynamically during follow-up and drug doses were not collected. The full text reported only on-drug incidence rates (1.13/100 person-years with prophylaxis and 2.74/100 person-years without prophylaxis), which could not be converted into stable node-specific per-arm events/total. No TMP-SMX adverse-event discontinuation data were available, and this was not a second-line intolerance study.	A-E; B; C
Aqeel, F. 2023	*Pneumocystis jirovecii* Pneumonia Prophylaxis in Patients with ANCA Vasculitis on Rituximab Maintenance Therapy	Embase; Web of Science	Insufficient extractable data for prespecified analyses	A corresponding full-length peer-reviewed report (Aqeel et al., 2024) was identified and evaluated. Prophylaxis exposure remained mixed across TMP-SMX, dapsone, and atovaquone; drug-specific events/denominators and adverse-event discontinuations were not extractable for the prespecified routes.	B; C
Shan, W. 2024	Clinical Characteristics and Epidemiological Analysis of *Pneumocystis Jirovecii* Pneumonia Infection in Kidney Transplant Patients with Trimethoprim-Sulfamethoxazole Dose Reduction Prophylaxis Strategy.	PubMed/MEDLINE; Embase; Web of Science	Wrong intervention/comparison or wrong outcome focus	Kidney transplant population, but the study mainly analysed clinical/epidemiological features of PJP cases under a low-dose TMP-SMX strategy. No usable comparator group or TMP-SMX adverse-event discontinuation data were available.	A-E; B

First author or registry, year	Title or short citation	Source/database	Primary exclusion reason category	Detailed reason	Potential route considered
2024	Efficacy and Safety of Prophylactic Treatment for *Pneumocystis Jirovecii* Pneumonia (PJP) in Patients With Autoimmune Inflammatory Rheumatic Disease (AIIRD)	Embase	Ineligible study design / non-original report / no final study results	ClinicalTrials.gov registered study that was still recruiting and had no published results. Only design and outcome definitions were available; no events/total data were available.	None
Cao, D. 2025	Efficacy and Safety of Low-Dose Trimethoprim-Sulfamethoxazole (TMP-SMZ) for the Prevention of *Pneumocystis jirovecii* pneumonia (PJP) in Non-HIV, Non-Transplant Immunosuppressed Patients: A Real-World Cohort Study.	PubMed/MEDLINE; Embase; Web of Science	Wrong intervention/comparison or wrong outcome focus	Although this compared low-dose versus conventional-dose TMP-SMX, the full text included neoplastic diseases such as solid and haematologic malignancies, and the target non-malignant subgroup could not be extracted separately.	A-S
Simpson, J. 2025	IS DAPSONE SAFE FOR PNEUMOCYSTIS JIROVECII PNEUMONIA PROPHYLAXIS IN KIDNEY AND PANCREAS TRANSPLANT RECIPIENTS?:	Embase; Web of Science	Conference abstract only; no corresponding full-length peer-reviewed report identified	Conference abstract only; no corresponding full-length peer-reviewed report was identified, and the available information was insufficient for reliable data verification and risk-of-bias assessment.	A-E; B; C
Wang, R. 2025	Low-Dose Sulfamethoxazole-Trimethoprim Could Prevent Pneumocystis jiroveci Pneumonia in Kidney Transplant Recipients: A Retrospective, Observational Study.	PubMed/MEDLINE; Embase; Web of Science	Insufficient extractable data for prespecified analyses	This was a retrospective observational/case-control study of PJP cases and matched controls, reporting 31 PJP cases and 124 controls. Standard per-arm prophylaxis events/total and discontinuation data were not extractable.	B
Chantapoh, W. 2025; 2024	Daily Single-Strength vs. Thrice-weekly Double-Strength Trimethoprim/ sulfamethoxazole for *Pneumocystis jirovecii* Prophylaxis in Kidney Transplant Recipients: A Non-inferiority Randomized Controlled trial	PubMed/MEDLINE; Embase; CENTRAL; Web of Science	Ineligible or non-separable population	This kidney transplant RCT described an adult population but allowed participants aged ≥15 years, and adult-only data were not separately extractable. It was therefore excluded from the primary adult-only synthesis and retained only as a sensitivity/contextual candidate where applicable.	A-E; A-S; B
Ruehman, A.J. 2026	Infrequent PJP Prophylaxis in Non-HIV, Non-Transplant Adults Receiving Immunosuppressants Diagnosed with PJP	Embase	Conference abstract only; no corresponding full-length peer-reviewed report identified	Conference abstract only; no corresponding full-length peer-reviewed report was identified, and the available information was insufficient for reliable data verification and risk-of-bias assessment.	B
Hardy AM 1984	Pneumocystis carinii pneumonia in renal-transplant recipients treated with cyclosporine and steroids.	PubMed/MEDLINE; Embase	Wrong intervention/comparison or wrong outcome focus	Kidney transplant population was eligible, and the article reported 14 PCP cases among 156 recipients receiving cyclosporine plus steroids, followed by introduction of TMP-SMX prophylaxis with no further PCP. However, it was not a formal prophylaxis-comparison study, did not report a clear TMP-SMX prophylaxis denominator, and had no extractable discontinuation data.	A-E; A-S; B
McGonigle, R.J.S. 1988	Does cyclosporin A adversely affect Pneumocystis carinii infection?	PubMed/MEDLINE; Embase	Wrong intervention/comparison or wrong outcome focus	This was primarily an analysis of PCP cases/outcomes under different immunosuppression backgrounds and raised the question of whether prophylaxis was needed. It was not a PCP prophylaxis study.	None
Mallolas, J 1991	Low-dose co-trimoxazole, aerosolised pentamidine, or dapsone plus pyrimethamine for prevention of Pneumocystis carinii pneumonia	CENTRAL	Wrong intervention/comparison or wrong outcome focus	This was a PCP prophylaxis study in HIV/AIDS. PubMed MeSH terms indicated AIDS complications, and subsequent full reports clearly involved HIV-infected patients.	None
Coker, RJ 1992	Co-trimoxazole versus dapsone-pyrimethamine for prevention of Pneumocystis carinii pneumonia	CENTRAL	Ineligible study design / non-original report / no final study results	Short Lancet correspondence/letter in the context of PCP prophylaxis trials. PubMed MeSH terms included CD4-positive T lymphocytes, and the related literature chain indicated HIV/AIDS PCP prophylaxis; it was not a non-HIV population.	None
Spieker, C. 1992	Pentamidine inhalation as a prophylaxis against Pneumocystis carinii pneumonia after therapy of acute renal allograft rejection with orthoclone (OKT3).	PubMed/MEDLINE; Embase	Duplicate / overlapping cohort or preliminary report	Highly consistent with the earlier Spieker/Barenbrock 1992 renal-transplant OKT3 pentamidine study and should be merged with that report to avoid duplicate counting. If data from this study are used, they should be handled as the same cohort.	A-E; C

First author or registry, year	Title or short citation	Source/database	Primary exclusion reason category	Detailed reason	Potential route considered
Eisen, H.J. 1994	PREVENTION OF PNEUMOCYSTIS-CARINII PNEUMONIA WITH TRIMETHOPRIM/SULFAMETHOXAZOLE OR PENTAMIDINE PROPHYLAXIS IN CARDIAC TRANSPLANT RECIPIENTS	Web of Science	Duplicate / overlapping cohort or preliminary report	The available file did not correspond to the target Rajendran record and was unrelated to PCP prophylaxis. The target report remained unavailable, and no extractable PCP events, denominators, or discontinuation data could be confirmed.	A-E; A-S; B
Nathan, S.D. 1994	Utility of inhaled pentamidine prophylaxis in lung transplant recipients.	PubMed/MEDLINE; Embase; Web of Science	Wrong intervention/comparison or wrong outcome focus	Small single-arm study of inhaled pentamidine after lung transplantation (n=9), reporting no PCP and two cases of bronchospasm. There was no comparator group and no clear TMP-SMX-intolerance second-line cohort, so it was excluded under the simplified current rules.	A-S; B; C
Branten, A.J.W. 1995	Pneumocystis carinii pneumonia in renal transplant recipients.	PubMed/MEDLINE; Embase; CENTRAL; Web of Science	Insufficient extractable data for prespecified analyses	A report abstract was retrieved, but the prophylaxis denominator was given only as >140 and comparator events/denominators were incomplete; no stable arm-level estimate could be extracted.	A-E
Okada, J 1997	Efficacy of trimethoprim-sulfamethoxazole for prophylaxis of Pneumocystis carinii pneumonia in patients with connective tissue disease.	Web of Science	Duplicate / overlapping cohort or preliminary report	Early conference/record version of the Okada study. The full article was available and reported CTD patients with ST prophylaxis 0/37 and no-ST approximately 2/47 PCP; it should be merged with the full publication and not counted separately. The abstract reported adverse events/laboratory abnormalities, but not clear discontinuation due to adverse events, so it was not used for A-S/B.	A-E; A-S; B
Chung, JB 1998	Cost effectiveness analysis of Pneumocystis carinii pneumonia (PCP) prophylaxis with trimethoprim-sulfamethoxazole (TMP-SXT) in patients with systemic lupus erythematosus (SLE) or Wegener's granulomatosis (WG) on immunosuppressive therapy.	Web of Science	Ineligible study design / non-original report / no final study results	This was a cost-effectiveness/model-based study of PCP prophylaxis, not an original clinical comparative study; per-arm PCP events/total or discontinuation events/total could not be extracted.	B
Halme, M. 1999	Breakthrough Pneumocystis carinii infections in lung and heart-lung transplant patients with chemoprophylaxis.	PubMed/MEDLINE; Embase; Web of Science; Wanfang	Wrong intervention/comparison or wrong outcome focus	The title indicated breakthrough PCP during chemoprophylaxis, but no extractable grouped data were found. Later literature cited it only as a breakthrough infection/lung-transplant PCP reference. Even if case counts were available, it did not appear to be a parallel comparative prophylaxis study and was not eligible for A/B/C.	B
Gordon, SM 1999	Should prophylaxis for Pneumocystis carinii pneumonia in solid organ transplant recipients ever be discontinued?	Web of Science	Insufficient extractable data for prespecified analyses	Among 1299 solid-organ transplant recipients, 25 PCP cases were identified. The study mainly addressed whether prophylaxis could be stopped/long-term risk; the abstract stated that no patients developed PCP while receiving prophylaxis, but clear prophylaxis versus no-prophylaxis per-arm events/total and discontinuation data were not extractable.	B
Rubík, I. 2001	Clinical, prophylactic and epidemiological study of pneumocystis carinii infection in immunocompromised patients after kidney transplantation	Embase	Ineligible study design / non-original report / no final study results	This study mainly detected Pneumocystis DNA in kidney transplant recipients and the ward environment to guide prevention/environmental decontamination. It was not an efficacy or safety study of prophylaxis regimens.	B; C
Nahimana A 2003	*Pneumocystis jirovecii* dihydrofolate reductase polymorphisms associated with failure of prophylaxis	Wanfang	Ineligible study design / non-original report / no final study results	Molecular resistance/polymorphism study using specimens from patients with PCP and analysing DHFR/DHPS mutations according to prophylaxis failure. It had no at-risk denominator and was not a prophylaxis regimen comparison.	None
Enomoto, T. 2005	[Pneumocystis jiroveci pneumonia as a complication of glucocorticoid therapy for interstitial pneumonia].	PubMed/MEDLINE; Embase	Duplicate / overlapping cohort or preliminary report	In 74 patients with interstitial pneumonia receiving corticosteroids, seven PCP cases occurred and none occurred among those receiving TMP-SMX. This appeared highly similar to the later English full report by Enomoto 2008 and was treated as a likely overlapping/early Japanese report rather than counted separately.	A-E

First author or registry, year	Title or short citation	Source/database	Primary exclusion reason category	Detailed reason	Potential route considered
Liu, Q. 2005	Diagnosis and treatment of Pneumocystis carinii pneumonia after liver transplantation	Wanfang	Insufficient extractable data for prespecified analyses	Clinical diagnosis and treatment experience for one PCP case among 63 liver transplant recipients. The conclusion mentioned low-dose compound TMP-SMX prophylaxis, but prophylaxis/control-group events/total were not reported, and it was not a discontinuation or second-line prophylaxis study.	B; C
Jia, W. 2006	Diagnosis, treatment, and prevention of Pneumocystis carinii pneumonia after kidney transplantation	Wanfang	Ineligible study design / non-original report / no final study results	The abstract described diagnosis, treatment, and prevention recommendations in 13 kidney transplant recipients with PCP. It was a case series/treatment-experience report and did not provide comparative prophylaxis data.	None
Liu, Y. 2007	Clinical analysis of 29 cases of Pneumocystis carinii pneumonia after kidney transplantation	Wanfang	Ineligible study design / non-original report / no final study results	Clinical analysis of 29 PCP cases after kidney transplantation. This was a case series/treatment-experience report without an at-risk prophylaxis denominator or discontinuation data.	B
Sun, W. 2008	Infectious complications after renal transplantation in 154 cases	Embase	Ineligible study design / non-original report / no final study results	Mainly a review/retrospective analysis of infectious complications after kidney transplantation. It mentioned that routine TMP-SMX could reduce PCP but did not provide PCP prophylaxis groups, events/total, or discontinuation data.	B
Garvey, J.P. 2009	Trimethoprim-sulfamethoxazole induced acute interstitial nephritis in renal allografts; clinical course and outcome.	PubMed/MEDLINE; Embase; Web of Science	Wrong intervention/comparison or wrong outcome focus	Kidney transplant population; case series of TMP-SMX-associated acute interstitial nephritis. All 11 cases discontinued TMP-SMX after allograft dysfunction/biopsy-proven AIN, but the total TMP-SMX prophylaxis denominator was not available, so a discontinuation rate could not be calculated; it was also not a PCP incidence study.	B
Nikeghbalian, S. 2010	Comparison of two antibiotic prophylaxis regimens in liver transplant recipients: A randomized clinical trial	Embase; CENTRAL	Ineligible study design / non-original report / no final study results	This was a comparison of perioperative antibacterial prophylaxis regimens in liver transplantation. Both groups received fluconazole and TMP-SMX as background prophylaxis, so it was not a comparison of PCP prophylaxis regimens and did not report PCP events/total or TMP-SMX discontinuation data.	B
Asashima, H. 2011	Adverse events to trimethoprimsulfamethoxazole in connective tissue disease patients	Embase	Ineligible study design / non-original report / no final study results	CTD population was eligible. Ninety-two patients received prophylactic-dose TMP-SMX and three received PCP-treatment-dose TMP-SMX. The abstract reported 8 adverse events among 95 prophylaxis/treatment recipients combined, but did not clearly state the number of prophylaxis-dose patients who discontinued due to adverse events; the three treatment-dose PCP patients with discontinuation did not belong to the prophylaxis outcome. It was not eligible for B and had no comparator for A.	A-E; B
Katchamart, W 2011	Methotrexate and Trimethoprim-Sulfamethoxazole for Pneumocystis pneumonia Prophylaxis replies	Web of Science	Wrong intervention/comparison or wrong outcome focus	Comment/reply in Journal of Rheumatology with no original extractable data.	None
Phipps, L.M. 2011	Nosocomial *Pneumocystis jirovecii* pneumonia: lessons from a cluster in kidney transplant recipients.	PubMed/MEDLINE; Embase; Web of Science	Ineligible study design / non-original report / no final study results	Kidney transplant PJP outbreak/transmission investigation including 14 cases and 324 clinic controls. The focus was transmission and risk factors; although TMP-SMX prophylaxis was reintroduced after the outbreak, the study did not provide an extractable prophylaxis-regimen comparison.	None
Struijk, G.H. 2011	Risk of Pneumocystis jiroveci pneumonia in patients long after renal transplantation.	PubMed/MEDLINE; Embase; Web of Science	Insufficient extractable data for prespecified analyses	Kidney transplant case-control study of 9 PCP cases and 18 controls, designed to identify long-term PJP risk factors and the need for prolonged prophylaxis. Per-arm prophylaxis versus no-prophylaxis events/total and discontinuation data were not extractable.	B

First author or registry, year	Title or short citation	Source/database	Primary exclusion reason category	Detailed reason	Potential route considered
Chen, T. 2011	Clinical study of compound sulfamethoxazole for prevention of Pneumocystis carinii pneumonia after kidney transplantation	Wanfang	Duplicate / overlapping cohort or preliminary report	Conference version of the same study/data as the formal Chen 2011 journal article, with 73 recipients receiving 6-month prophylaxis, 47 receiving 1-year prophylaxis, and 5 not receiving prophylaxis, with consistent event counts. The formal journal article was retained and this record was not counted separately.	None
Nickel, P 2012	Clindamycin-Primaquine Is Safe in Treating Pneumocystis Pneumonia in Renal Transplant Patiens but Appears to Be Less Effective than Trimethoprim/Sulfamethoxazole	Web of Science	Wrong intervention/comparison or wrong outcome focus	Treatment study in renal transplant recipients with PCP, comparing clindamycin-primaquine with TMP-SMX treatment. It was not a prophylaxis study.	None
Kim, T. 2012	No recurrence of *Pneumocystis jirovecii* Pneumonia after solid organ transplantation regardless of secondary prophylaxis.	PubMed/MEDLINE; Embase; Web of Science	Wrong intervention/comparison or wrong outcome focus	This study involved solid-organ transplant recipients who had already developed and recovered from PCP, and compared recurrence according to whether secondary prophylaxis was given. It did not address first/breakthrough PCP prevention in the target prophylaxis framework and was not a TMP-SMX-intolerance second-line analysis. Among 41 recovered patients, 22 received secondary prophylaxis and none had recurrence.	A-S; C
Fillatre, P. 2014	Incidence of Pneumocystis jiroveci pneumonia among groups at risk in HIV-negative patients.	PubMed/MEDLINE; Embase; Web of Science	Insufficient extractable data for prespecified analyses	Epidemiologic/incidence study of PCP in HIV-negative risk groups, estimating incidence across underlying diseases. It did not provide prophylactic-drug node-specific prophylaxis versus no-prophylaxis events/total or discontinuation data.	B
Chandola, P. 2014	Outbreak of *Pneumocystis jirovecii* pneumonia in renal transplant recipients on prophylaxis: Our observation and experience	Embase; Web of Science	Ineligible study design / non-original report / no final study results	Kidney transplant PJP outbreak case report/small case series, with seven PJP cases, including two breakthrough cases during prophylaxis. No at-risk denominator or usable comparator group was available.	B
Lu, J.J. 2014	Two cases of P neumocystis carinii pneumonia (PCP) as a complication of azathioprine therapy in Crohn's disease-a need for prophylaxis	Embase	Ineligible study design / non-original report / no final study results	Case reports/treatment experience of two Crohn's disease patients who developed PCP. The report only raised the question of prophylaxis and did not provide prophylaxis-group data.	None
Tan, W. 2014	“Two-hit” hypothesis: Pneumocystis pneumonia and digeorge syndrome	Embase	Insufficient extractable data for prespecified analyses	Single case, involving DiGeorge syndrome/primary immunodeficiency background. It was not a prophylaxis study and provided no events/total.	None
Sekhon, G. 2015	Late onset cluster of *pneumocystis jirovecii* pneumonia (PJP) in renal transplant patients: Re-examining prophylaxis strategies	Embase; Web of Science	Ineligible study design / non-original report / no final study results	Kidney transplant late-onset PJP cluster with seven confirmed cases, all not receiving PJP prophylaxis at diagnosis. This was an outbreak/case series and did not provide prophylaxis-comparison denominators or discontinuation data.	B
Iriart, X. 2015	Risk factors of Pneumocystis pneumonia in solid organ recipients in the era of the common use of posttransplantation prophylaxis.	PubMed/MEDLINE; Embase; Web of Science	Insufficient extractable data for prespecified analyses	Solid-organ transplant population was eligible, but this was a PCP case-control risk-factor study. Thirty-three PCP cases were matched to controls, and all patients had received six months of postoperative TMP-SMX prophylaxis. It did not provide NMA-usable prophylaxis versus no-prophylaxis events/total or discontinuation rates.	A-S; B
Bates, D.M. 2015	Suspected sulfa-resistant *pneumocystis jirovecii* leading to prophylaxis failure and pneumonia in an HIV-negative, immunocompromised adult	Embase; Web of Science	Conference abstract only; no corresponding full-length peer-reviewed report identified	Conference abstract only; no corresponding full-length peer-reviewed report was identified, and the available information was insufficient for reliable data verification and risk-of-bias assessment.	None
Rege, A. 2015	“Unusual rare infections” following intestinal transplantation	Embase	Conference abstract only; no corresponding full-length peer-reviewed report identified	Conference abstract only; no corresponding full-length peer-reviewed report was identified, and the available information was insufficient for reliable data verification and risk-of-bias assessment.	B

First author or registry, year	Title or short citation	Source/database	Primary exclusion reason category	Detailed reason	Potential route considered
Nicolas, A. 2016	Compared efficacies of lung delivery using two nebulizers in the prophylaxis against pneumocystis carinii pneumonia	Embase; Web of Science	Wrong intervention/comparison or wrong outcome focus	Study of pulmonary delivery efficiency of nebuliser devices in healthy volunteers using amikacin as a tracer. It did not involve immunosuppressed patients or PCP clinical outcomes.	None
Salmela, A. 2017	Chronic nasal Staphylococcus aureus carriage identifies a subset of newly diagnosed granulomatosis with polyangiitis patients with high relapse rate.	PubMed/MEDLINE; Embase; CENTRAL; Web of Science	Insufficient extractable data for prespecified analyses	AAV population was eligible, but the study focused on Staphylococcus aureus nasal carriage and relapse. TMP-SMX prophylaxis in 55/200 patients was background information only, with no PCP events/total or discontinuation rates.	B
Fares, S. 2017	Early and late infectious complications after living kidney transplantation	Embase	Wrong intervention/comparison or wrong outcome focus	Abstract on infectious complications after kidney transplantation. All patients received SMX-TMP as background PJP prophylaxis; the study focused on early and late infection complications and had no comparative PCP prophylaxis groups.	None
Takahashi, K. 2017	INCIDENCE OF PNEUMOCYSTIS JIROVECCI PNEUMONIA AND PROPHYLACTIC ADMINISTRATION OF TRIMETHOPRIM-SULFAMETHOXAZOLE IN ANTI-NEUTROPHIL CYTOPLASMIC ANTIBODY-ASSOCIATED VASCULITIS	Embase; Web of Science	Conference abstract only; no corresponding full-length peer-reviewed report identified	Conference abstract only; no corresponding full-length peer-reviewed report was identified, and the available information was insufficient for reliable data verification and risk-of-bias assessment.	A-E; B
Nakaya, I. 2017	Prophylactic treatment and incidence of pneumocystis jirovecci pneumonia in Japanese patients with antineutrophil cytoplasmic antibody-associated vasculitis	Embase; Web of Science	Duplicate / overlapping cohort or preliminary report	Same RemIT-JAV-RPGN AAV cohort as the later Nakaya 2023 full publication (319 patients). The full text was judged to involve dynamic chemoprophylaxis exposure that could not be converted into stable drug-node per-arm events/total, so it was not included in A/B/C; this conference abstract was not counted separately.	B; C
Nunokawa, T 2017	THE PROPHYLACTIC EFFECT OF SULFASALAZINE AGAINST PNEUMOCYSTIS PNEUMONIA: AN ANALYSIS OF INCIDENCE FOR PNEUMOCYSTIS PNEUMONIA IN PATIENTS WITH RHEUMATOID ARTHRITIS IN JAPAN	Web of Science	Wrong intervention/comparison or wrong outcome focus	RA population was eligible, but the study drug was sulfasalazine rather than a target node in this NMA (TMP-SMX, atovaquone, dapsone, pentamidine, or no prophylaxis). It was not eligible for the intervention network.	A-S; C
Salim SA 2017	Upward trend of dapsone-induced methemoglobinemia in renal transplant community	PubMed/MEDLINE; Embase; Web of Science	Insufficient extractable data for prespecified analyses	Small case series of four kidney transplant recipients who developed methemoglobinemia after switching from TMP-SMX to dapsone. No total second-line dapsone denominator was available, so an event rate could not be extracted.	C
Kono M 2018	A Case of a Pneumocystis Pneumonia Twenty-four Years After Living Kidney Transplantation Due to Withdrawal of Sulfamethoxazole/Trimethoprim Prophylaxis.	PubMed/MEDLINE	Ineligible study design / non-original report / no final study results	Single letter/case report: PJP occurred 24 years after kidney transplantation following TMP-SMX discontinuation. No prophylaxis events/total were extractable, and it was not eligible for B/C.	A-S; B
LoPiccolo J 2018	Further Lessons in Pneumocystis Pneumonia Prophylaxis-Reply.	PubMed/MEDLINE	Ineligible study design / non-original report / no final study results	RA biologics population; 82 patients received TMP-SMX prophylaxis and 11 switched to aerosolized pentamidine because of adverse events. The record was considered for B (TMP-SMX adverse-event discontinuation 11/82) and C (11 switches to pentamidine with no PCP in the second phase), but it lacked a clear randomized or contemporaneous no-prophylaxis comparator for the main NMA.	A-E; B; C
Park, S.Y. 2018	*Pneumocystis jirovecii* pneumonia in renal transplant recipients afer a 6-month trimethoprim-sulfamethoxazole prophylaxis: A case-control study	Embase	Duplicate / overlapping cohort or preliminary report	Conference abstract version of the same study as the Park 2020 full report. Patients had completed and discontinued six months of TMP-SMX, and the study analysed risk factors for PCP after discontinuation rather than comparing prophylaxis regimens. The full report was treated as the main record, but it was also not eligible for A/B/C.	B
Yukawa, K. 2018	Risk Factors for *Pneumocystis jirovecii* Pneumonia in Patients With Rheumatoid Arthritis and a Prophylactic Indication of Trimethoprim/Sulfamethoxazole.	PubMed/MEDLINE; Embase; Web of Science	Wrong intervention/comparison or wrong outcome focus	RA population was eligible, but the analysis included 2640 RA patients who did not receive TMP-SMX prophylaxis and was used to identify PCP risk factors/prophylaxis indications. There was no actual prophylaxis group and no extractable prophylaxis versus no-prophylaxis comparison.	None

First author or registry, year	Title or short citation	Source/database	Primary exclusion reason category	Detailed reason	Potential route considered
Sharma, N. 2018	Spectrum of infections in kidney transplant recipients with rATG induction	Embase	Ineligible study design / non-original report / no final study results	Kidney transplant population; TMP-SMX was background prophylaxis for all or most patients. The study focused on infection patterns after rATG rather than PCP prophylaxis regimens and had no discontinuation rate.	B
Nieto-Ríos, J.F. 2018	[Severe hypoxemic respiratory failure caused by *Pneumocystis jirovecii* in a late kidney transplant recipient].	PubMed/MEDLINE; Embase; Web of Science	Ineligible study design / non-original report / no final study results	Two late-onset PJP cases after kidney transplantation, related to TMP-SMX discontinuation. This was a case report/case series without an at-risk denominator and was not eligible for A/B/C.	B
Morilla, R 2019	Airborne acquisition of Pneumocystis in bronchoscopy units: a hidden danger to healthcare workers	Web of Science	Wrong intervention/comparison or wrong outcome focus	Study of oropharyngeal samples from healthcare workers and air samples from bronchoscopy suites, focusing on environmental transmission/molecular epidemiology. It was not a prophylaxis study in the target immunosuppressed population.	None
Melexopoulou, C. 2019	Late-onset pneumonocystis jiroveci in kidney transplant recipients. A single center experience	Embase	Ineligible study design / non-original report / no final study results	Late-onset PCP case series of 12 confirmed cases. Most patients had previously received early prophylaxis, but no at-risk denominator by prophylaxis regimen or discontinuation data were available.	B
Argy, N 2019	Pneumocystis cytochrome b mutants associated with atovaquone prophylaxis failure as the cause of Pneumocystis infection outbreak among heart transplant recipients (vol 67, pg 913, 2018)	Web of Science	Ineligible study design / non-original report / no final study results	Correction/erratum to the separately assessed Argy 2018 report; not an independent original report.	C
Akashi, K 2019	The Comparative Efficacy of Pneumocystis Pneumonia Prophylactic Regimens in Patients with Connective Tissue Diseases Receiving Prolonged High-dose Glucocorticoids	Web of Science	Duplicate / overlapping cohort or preliminary report	A more complete full-text article was available. The formal report provided TMP-SMX versus atovaquone PCP incidence (2/281 vs 5/107), early TMP-SMX adverse-event discontinuation (102/383), and atovaquone discontinuation 0/107; it should be handled under the formal full-text record rather than this preliminary/duplicate record.	A-E; A-S; B; C
Park, S.Y. 2020	Epidemiology and risk factors associated with *Pneumocystis jirovecii* pneumonia in kidney transplant recipients after 6-month trimethoprim-sulfamethoxazole prophylaxis: A case-control study.	PubMed/MEDLINE; Embase; Web of Science	Insufficient extractable data for prespecified analyses	Kidney/kidney-pancreas transplant population. In 3941 recipients, 67 PCP cases occurred after completion and discontinuation of 6 months of TMP-SMX prophylaxis. The formal study was a case-control risk-factor analysis and did not provide continuing-prophylaxis versus no-prophylaxis per-arm events/total or discontinuation rates.	B
Sahu, K.K. 2020	Methemoglobinemia in Transplant Recipient Patients: Future of Dapsone as Pneumocystis carinii Pneumonia Prophylaxis.	PubMed/MEDLINE; Embase; Web of Science	Ineligible study design / non-original report / no final study results	Two-page commentary/therapeutic note discussing dapsone-related methemoglobinemia, mainly citing existing cases and the Mitsides study. No new extractable cohort denominator or events/total were reported.	C
Delbove, A. 2020	Pneumocystis pneumonia after lung transplantation: A retrospective multicenter study.	PubMed/MEDLINE; Embase; Web of Science	Ineligible study design / non-original report / no final study results	Lung transplant population was eligible, but the study included 47 patients who had already developed PCP and described incidence, treatment, and outcomes. No at-risk denominator by prophylaxis regimen was available.	None
Young, N. 2020	Pneumocystis pneumonia in HIV-negative adults: missed opportunities for prevention	Embase	Wrong intervention/comparison or wrong outcome focus	HIV-negative adult PCP case study focused on 'potentially preventable PCP', but it included 108 patients who had already developed PCP. There was no total prophylaxis/non-prophylaxis denominator, and many patients had solid tumours, haematologic malignancy, or stem-cell transplantation.	None
Kim, Y.H. 2020	Pneumocystis pneumonia occurrence and prophylaxis duration in kidney transplant recipients according to perioperative treatment with rituximab	Embase; Web of Science	Ineligible study design / non-original report / no final study results	Kidney transplant population; all recipients received TMP-SMX prophylaxis according to protocol. Although some patients could not maintain TMP-SMX because of adverse effects, the study primarily examined rituximab and prophylaxis duration/PCP risk after discontinuation rather than clear drug-node comparisons; it was not eligible for A-E/A-S.	A-E; A-S; B

First author or registry, year	Title or short citation	Source/database	Primary exclusion reason category	Detailed reason	Potential route considered
Li, T. 2020	Clinical characteristics of PCP in living-donor kidney transplant recipients	Wanfang	Ineligible study design / non-original report / no final study results	Thesis including 24 living-donor kidney transplant recipients with confirmed PCP and analysing clinical characteristics and treatment. It was not a prophylaxis-comparison study and had no at-risk denominator or discontinuation rate.	B
Siscos, S.M. 2021	Advantages of dapsone over trimethoprim/sulfamethoxazole for Pneumocystis pneumonia prophylaxis in patients with immunobullous disease	Embase	Insufficient extractable data for prespecified analyses	Therapeutic Pearl/treatment recommendation article in JAAD, not an original study. It proposed dapsone as both disease treatment and PCP prophylaxis in BP/PV patients but did not provide sample size, PCP events, discontinuation events, or extractable events/total.	B; C
Pett, D. 2021	Practice patterns in *pneumocystis jirovecii* pneumonia prophylaxis in solid organ and bone marrow transplant recipients	PubMed/MEDLINE; Embase; Web of Science	Conference abstract only; no corresponding full-length peer-reviewed report identified	Conference abstract only; no corresponding full-length peer-reviewed report was identified, and the available information was insufficient for reliable data verification and risk-of-bias assessment.	A-E; B; C
Hosseini-Moghaddam, S.M. 2021	Six-month risk of Pneumocystis pneumonia following acute cellular rejection: A case-control study in solid organ transplant recipients	Embase; Web of Science	Insufficient extractable data for prespecified analyses	SOT population was eligible, but this was a case-control risk-factor study of 15 PCP cases and 60 controls, concluding that acute rejection/CMV were associated with PCP risk. It did not provide prophylactic-drug node-specific events/total or discontinuation rates.	B
Marinaki, S. 2021	The Changing Landscape of Pneumocystis Jiroveci Infection in Kidney Transplant Recipients: Single-Center Experience of Late-Onset Pneumocystis Pneumonia.	PubMed/MEDLINE; Embase; Web of Science	Ineligible study design / non-original report / no final study results	Kidney transplant population, but this was a case series of 12 late-onset PCP cases. Most patients had previously received prophylaxis, but no at-risk denominator by prophylaxis regimen or discontinuation data were available.	B
Yang, P. 2021	The risk factor analysis and treatment experience in *pneumocystis jirovecii* pneumonia after kidney transplantation.	PubMed/MEDLINE; Embase; Web of Science	Ineligible study design / non-original report / no final study results	Kidney transplant population; 47 PJP cases were compared with 120 non-PJP pneumonia controls to study PJP risk factors and treatment experience. It was not a prophylaxis-regimen comparison and did not provide prophylaxis events/total.	None
Claytor, B. 2022	Opinions on *Pneumocystis jirovecii* prophylaxis in autoimmune neuromuscular disorders	Embase; Web of Science	Ineligible study design / non-original report / no final study results	Opinion/commentary article discussing whether PJP prophylaxis is needed in autoimmune neuromuscular diseases. No original data or events/total were available.	None
Gu, ZY 2022	Preliminary Study on the Combination Effect of Clindamycin and Low Dose Trimethoprim-Sulfamethoxazole on Severe Pneumocystis Pneumonia After Renal Transplantation	Web of Science	Wrong intervention/comparison or wrong outcome focus	Kidney transplant population, but the study included 20 patients with severe established PCP and compared low-dose TMP-SMX plus clindamycin versus standard-dose TMP-SMX for treatment. It was not a prophylaxis study.	None
Neofytos, D. 2023	Bacteremia During the First Year After Solid Organ Transplantation: An Epidemiological Update.	PubMed/MEDLINE; Embase; Web of Science	Wrong intervention/comparison or wrong outcome focus	SOT population, but the topic was first-year post-transplant bacteraemia epidemiology. Cotrimoxazole/PJP prophylaxis was only a covariate in a bacteraemia risk model and did not provide PCP incidence or discontinuation rates.	B
Ardhe, A. 2023	Impact of thrice-weekly cotrimoxazole prophylaxis on creatinine and potassium plasma levels in kidney transplant recipients.	PubMed/MEDLINE; Embase; Web of Science	Insufficient extractable data for prespecified analyses	Kidney transplant population in which all patients received thrice-weekly cotrimoxazole. The focus was changes in creatinine and potassium after discontinuation; PCP events/total and discontinuation due to adverse events were not reported.	A-E; B
Moumen, M. 2023	Retrospective analysis of risks and benefits of trimethoprim/sulfamethoxazole as prophylaxis for Pneumocystis jiroveci pneumonia in patients with dermatologic disease	Embase	Insufficient extractable data for prespecified analyses	FAERS spontaneous adverse-event reporting database analysis, not a clinical cohort. It reported prednisone-associated PCP reports and serious cutaneous reaction reports related to TMP-SMX prophylaxis, but there was no true exposure denominator and no extractable PCP incidence or TMP-SMX adverse-event discontinuation rate.	A-E; B

First author or registry, year	Title or short citation	Source/database	Primary exclusion reason category	Detailed reason	Potential route considered
Pates, K. 2023	Rising incidence of Pneumocystis pneumonia: A population-level descriptive ecological study in England.	PubMed/MEDLINE; Embase; Web of Science	Insufficient extractable data for prespecified analyses	Population-level descriptive ecological study of PCP hospitalisation incidence and TMP-SMX consumption trends in England. No individual-level prophylaxis/control-group events/total or discontinuation rates were available.	B
Van Leerdam, T. 2023	Surveillance of Life-Long Antibiotics (SOLLA) 2: Patients on Life-Long Antibiotics in an Australian Healthcare Network	Embase	Ineligible study design / non-original report / no final study results	Long-term antibiotic prescribing surveillance study. The topic was not PCP prophylaxis effectiveness or safety, and no target outcomes/events/total were available.	A-E
Mendel, A. 2023	Use and Persistence of Trimethoprim Sulfamethoxazole Prophylaxis in Patients with Granulomatosis with Polyangiitis Treated with Rituximab	Embase	Insufficient extractable data for prespecified analyses	Adult GPA plus rituximab population was eligible, but the study objective was TMP-SMX use and persistence. It reported that 426/1877 used TMP-SMX but did not report PCP events/total or adverse-event discontinuation counts.	A-E; B
Lee, WI 2024	Antibiotic prophylaxis in immunosuppressed patients - Missed opportunities from trimethoprim-sulfamethoxazole allergy label.	PubMed/MEDLINE; Web of Science	Ineligible study design / non-original report / no final study results	Review/commentary article discussing TMP-SMX allergy labels, desensitisation, and alternative prophylaxis strategies. No original data were available.	C
Onishi, T. 2024	Clinical practice pattern of Pneumocystis pneumonia prophylaxis in systemic lupus erythematosus: a cross-sectional study from lupus registry of nationwide institutions (LUNA)	Embase; Web of Science	Wrong intervention/comparison or wrong outcome focus	SLE population was eligible, but this was a cross-sectional practice-pattern study describing that 21% of 1460 patients received PCP prophylaxis and its association with glucocorticoid dose. PCP incidence comparisons and discontinuation rates were not provided.	B
Sendo Sho 2025	A Study Using a Low-Dose Antibiotic to Prevent Pneumocystis Pneumonia in People Receiving Immunosuppressive Treatment	WHO ICTRP	Ineligible study design / non-original report / no final study results	Trial registry record of low-dose TMP-SMX prophylaxis for PJP in immunosuppressed patients. The design was relevant, but no results were available.	None
Hansen, D.L. 2025	AIHA and rituximab: time for Pneumocystis prophylaxis?	Embase	Ineligible study design / non-original report / no final study results	Editorial/note in Blood Advances discussing whether PCP prophylaxis is needed for AIHA treated with rituximab. It was not an original study and had no events/total.	None
Papanikolla, J. 2025	Retrospective Evaluation of Dapsone Induced Anemia in Kidney, Kidney/Pancreas, and Liver Solid Organ Transplant Recipients	Embase	Conference abstract only; no corresponding full-length peer-reviewed report identified	Conference abstract retrieved; results were incomplete and no full-length peer-reviewed report was identified.	B; C
Vetter, H. 1992	Incidence, prophylaxis and therapy of cytomegalovirus and pneumocystis carinii infection following heart transplantation	PubMed/MEDLINE; Embase; Web of Science	Ineligible study design / non-original report / no final study results	Heart transplantation-related article, likely a review/overview discussing CMV and PCP prophylaxis and treatment. No extractable grouped events/total were available.	None
Guillaume, S. 2000	Cost-effectiveness of prophylaxis against Pneumocystis carinii pneumonia in patients with Wegner's granulomatosis undergoing immunosuppressive therapy.	PubMed/MEDLINE; Embase; Web of Science	Ineligible study design / non-original report / no final study results	Cost-effectiveness Markov model, not an original clinical study; real patient events/total could not be extracted.	None
Rodriguez, M. 2004	Failure of low-dose atovaquone prophylaxis against Pneumocystis jiroveci infection in transplant recipients.	PubMed/MEDLINE; Embase; Web of Science	Ineligible study design / non-original report / no final study results	Short report/case report of two atovaquone prophylaxis failures. No total second-line atovaquone denominator was available, so a route C event rate could not be calculated.	C
Lee, I. 2005	Complications related to dapsone use for *Pneumocystis jirovecii* pneumonia prophylaxis in solid organ transplant recipients.	PubMed/MEDLINE; Embase; Web of Science	Insufficient extractable data for prespecified analyses	SOT population and dapsone safety topic, but the abstract indicated provider-identified dapsone-related haemolysis/methemoglobinemia cases and did not provide a complete dapsone exposure denominator, so an event rate could not be extracted.	C

First author or registry, year	Title or short citation	Source/database	Primary exclusion reason category	Detailed reason	Potential route considered
Trotter, J.F. 2008	Absence of Pneumocystis jiroveci pneumonia in liver transplantation recipients receiving short-term (3-month) prophylaxis.	PubMed/MEDLINE; Embase; Web of Science	Wrong intervention/comparison or wrong outcome focus	Single-arm centre experience with 3-month TMP-SMX after liver transplantation. No comparator group or TMP-SMX adverse-event discontinuation data were available.	A-E; B
Altintaş, G. 2011	Inhaled pentamidine for Pneumocystis jiroveci prophylaxis in a heart transplant recipient with allergy for trimethoprim sulfamethoxazole.	PubMed/MEDLINE; Embase; Web of Science	Ineligible study design / non-original report / no final study results	Single case report of inhaled pentamidine prophylaxis after TMP-SMX allergy in a heart transplant recipient. No events/total were available.	C
de Boer, M.G.J. 2011	Risk factors for *Pneumocystis jirovecii* pneumonia in kidney transplant recipients and appraisal of strategies for selective use of chemoprophylaxis.	PubMed/MEDLINE; Embase; Web of Science	Insufficient extractable data for prespecified analyses	Kidney transplant PCP case-control risk-factor study (50 PCP cases vs 100 controls). It was used for risk stratification rather than prophylaxis-drug node comparisons and did not provide extractable A/B/C data.	B; C
Shishido, T. 2012	[Case of trimethoprim-induced hyperkalemia complicating ANCA-associated vasculitis].	PubMed/MEDLINE; Embase	Ineligible study design / non-original report / no final study results	Single case report of hyperkalaemia/hyponatraemia after TMP-SMX prophylaxis, with a history of colon cancer. No total denominator was available, so it was not eligible for route B.	None
Evans, R. 2014	Efficacy of once weekly dapsone dosing for PCP prophylaxis post-transplantation.	Embase; Web of Science	Duplicate / overlapping cohort or preliminary report	Conference abstract of the same study as the full Evans 2015 article; it was not counted separately.	None
Kercsak, A. 2014	The effect of sulfamethoxazole/trimethoprim pcp prophylaxis on early post-kidney transplant urinary tract infections	Embase; Web of Science	Wrong intervention/comparison or wrong outcome focus	The study focused on TMP-SMX effects on post-transplant UTI/ASB and did not report PCP incidence or adverse-event discontinuation, so it was not eligible for A/B/C.	A-E; A-S; B
Karp, A. 2015	Methemoglobinemia in renal transplant recipients on dapsone for pneumocystis jiroveci pneumonia prophylaxis: A single center experience and quality improvement initiative	Embase; Web of Science	Conference abstract only; no corresponding full-length peer-reviewed report identified	Conference abstract only; no corresponding full-length peer-reviewed report was identified, and the available information was insufficient for reliable data verification and risk-of-bias assessment.	C
Singh, R. 2015	The impact of trimethoprim-sulfamethoxazole as pneumocystis pneumonia prophylaxis on the incidence of asymptomatic bacteriuria and urinary tract infections among renal allograft recipients	Embase	Wrong intervention/comparison or wrong outcome focus	Adult kidney transplant study comparing TMP-SMX versus no TMP-SMX for ASB/UTI. The target outcome was not PCP or adverse-event discontinuation, and the formal study also focused on ASB/UTI, so it was not suitable for A/B/C.	A-E; A-S; B
Singh, R. 2016	The impact of trimethoprim-sulfamethoxazole as Pneumocystis jiroveci pneumonia prophylaxis on the occurrence of asymptomatic bacteriuria and urinary tract infections among renal allograft recipients: a retrospective before-after study.	PubMed/MEDLINE; Embase; Web of Science	Wrong intervention/comparison or wrong outcome focus	Adult kidney transplant study in which TMP-SMX was background PJP prophylaxis, but the research objective was asymptomatic bacteriuria/urinary tract infection. PCP incidence or adverse-event discontinuation were not reported.	A-E; B
Goto N 2017	Lifelong Prophylaxis With Trimethoprim-Sulfamethoxazole for Prevention of Outbreak of *Pneumocystis jirovecii* Pneumonia in Kidney Transplant Recipients.	PubMed/MEDLINE; Web of Science	Insufficient extractable data for prespecified analyses	Kidney transplant outbreak-control study. Forty-eight PCP cases occurred when patients were not receiving prophylaxis, and no further outbreak occurred after lifelong TMP-SMX was introduced. However, stable per-arm prophylaxis versus no-prophylaxis events/total and TMP-SMX adverse-event discontinuation rates were not available; it was used only as discussion background.	A-E; B
Argy, N. 2018	Pneumocystis Cytochrome b Mutants Associated With Atovaquone Prophylaxis Failure as the Cause of Pneumocystis Infection Outbreak Among Heart Transplant Recipients.	PubMed/MEDLINE; Embase; Web of Science	Insufficient extractable data for prespecified analyses	Heart transplant atovaquone prophylaxis failure/outbreak/resistance-mechanism study. A complete atovaquone second-line prophylaxis exposure denominator was not available, so a route C event rate could not be calculated.	C
Havele, S. 2018	Safety of trimethoprim-sulfamethoxazole for PJP prophylaxis in patients taking methotrexate for chronic inflammatory or autoimmune diseases	Embase; Web of Science	Duplicate / overlapping cohort or preliminary report	Early conference abstract of the same study as the Havele 2021 JAAD research letter. The research letter was retained, and this abstract was not counted separately.	None

First author or registry, year	Title or short citation	Source/database	Primary exclusion reason category	Detailed reason	Potential route considered
Prasad, G.V.R. 2019	MON-189 SAFETY AND EFFICACY OF PROPHYLAXIS FOR PNEUMOCYSTIS JIROVECII PNEUMONIA INVOLVING TRIMETHOPRIM-SULFAMETHOXAZOLE DOSE REDUCTION IN KIDNEY TRANSPLANTATION	Embase	Duplicate / overlapping cohort or preliminary report	Conference abstract/full publication pair with the Prasad 2019 BMC Infectious Diseases article. The full article was retained, and this abstract was not counted separately.	A-S
Goto, M. 2020	Incidences of adverse effects and disease flare due to pneumocystis pneumonia prophylaxis with trimethoprim/ sulfamethoxazole in systemic lupus erythematosus patients	Embase; Web of Science	Conference abstract only; no corresponding full-length peer-reviewed report identified	Conference abstract only; no corresponding full-length peer-reviewed report was identified, and the available information was insufficient for reliable data verification and risk-of-bias assessment.	A-E; B
Lor, K.W. 2022	Dapsone-Associated Anemia in Heart Transplant Recipients with Normal Glucose-6-Phosphate Dehydrogenase Activity.	PubMed/MEDLINE; Embase; Web of Science	Wrong intervention/comparison or wrong outcome focus	Heart transplant population; dapsone was used as an alternative PJP prophylactic agent after sulfa allergy/intolerance, with significant anaemia and dapsone discontinuation reported in 8/36 and haemoglobin improvement after stopping. This was considered second-line dapsone safety information rather than an eligible comparative effectiveness or TMP-SMX discontinuation analysis.	B; C
Selznick, L. 2022	Incidence of Urinary Tract Infection or Asymptomatic Bacteriuria in Kidney Transplant Recipients with Sulfamethoxazole-Trimethoprim vs. Pentamidine for Pneumocystis Jiroveci Pneumonia Prophylaxis.	Embase; Web of Science	Wrong intervention/comparison or wrong outcome focus	Retrieved title/record concerns urinary tract infection or asymptomatic bacteriuria rather than a prespecified PCP effectiveness or treatment-limiting discontinuation endpoint.	A-S; C
Rahman, A.R.I.Q. 2024	PNEUMOCYSTIS PNEUMONIA DESPITE ATOVAQUONE PROPHYLAXIS IN A PATIENT ON LONG-TERM CORTICOSTEROIDS	Embase; Web of Science	Ineligible study design / non-original report / no final study results	Single case report of atovaquone prophylaxis failure in a long-term glucocorticoid recipient. No total second-line atovaquone denominator was available, so it was not eligible for route C.	C
Tongji Hospital 2026; 2024	Efficacy and Safety of Prophylactic Treatment for *Pneumocystis Jirovecii* Pneumonia in Patients With Autoimmune Inflammatory Rheumatic Disease	ClinicalTrials.gov; WHO ICTRP	Ineligible study design / non-original report / no final study results	Clinical trial registry record with relevant target population and intervention, but no results were available; events/total could not be extracted.	None
Zmarlicka, M. 2015	Tolerability of low-dose sulfamethoxazole/trimethoprim for *Pneumocystis jirovecii* pneumonia prophylaxis in kidney transplant recipients.	PubMed/MEDLINE; Embase; Web of Science	Wrong intervention/comparison or wrong outcome focus	Adult kidney transplant study in which 67/88 completed 1 year of low-dose SMX/TMP, 10 discontinued early, and 11 switched to atovaquone. Discontinuation reasons included hyperkalaemia, leukopenia, diarrhoea, and medication simplification. This information was considered for TMP-SMX discontinuation, but the record was not retained in the final dataset.	B; C
Que, Y. 2023	Analysis on Prophylaxis of Pneumocystis Carini Pneumonia by Trimethoprim-Sulfamethoxazole in Patients with Autoimmune Diseases	Embase; Wanfang	Ineligible study design / non-original report / no final study results	Chinese narrative review/analysis discussing PCP risk, TMP-SMX prophylactic effects, and adverse reactions in autoimmune disease; no original clinical events/total were available.	None
Kobayashi, T. 2014	THU0295 Prophylactic use of trimethoprim-sulfamethoxazole against Pneumocystis pneumonia may reduce the risk of bloodstream infection in patients with connective tissue diseases undergoing high-dose corticosteroid therapy	Previously included record	Duplicate / overlapping cohort or preliminary report	A later full-length peer-reviewed report (Honda et al., 2021) from the same center/authors covered an expanded overlapping cohort and was already included. Kobayashi 2014 was removed from Route B to avoid double counting.	B
Bonnet, M. 2018	5PSQ-031 Pneumocystis carinii pneumonia prevention in lung transplantation: is atovaquone effective?	Previously included record	Conference abstract only; no corresponding full-length peer-reviewed report identified	Conference abstract only; no corresponding full-length peer-reviewed report was identified, and the available information was insufficient for reliable data verification and risk-of-bias assessment.	C
Schumacher, L. 2023	Safety of Once-Weekly Dapsone for PJP Prophylaxis in Kidney Transplant Recipients	ACCP 2023 abstract supplement	Duplicate / overlapping cohort or preliminary report	Preliminary report of the included Schumacher 2025 full publication; excluded to avoid double counting.	C

First author or registry, year	Title or short citation	Source/database	Primary exclusion reason category	Detailed reason	Potential route considered
Vu, A. 2023	Is everything ok? Incidence of hyperkalemia after converting to daily TMP-SMX	ACCP 2023 abstract supplement	Conference abstract only; no corresponding full-length peer-reviewed report identified	Distinct conference abstract; no full-length peer-reviewed report was identified.	A-S; B
Rahman, S.; Wynd, M. 2023	Characterizing tolerability and outcomes of second-line agents used for PJP prophylaxis	ACCP 2023 abstract supplement	Conference abstract only; no corresponding full-length peer-reviewed report identified	Distinct conference abstract; no full-length peer-reviewed report was identified.	C

Panel B. Reports sought but not retrieved (n=5)

Report	Citation/topic	Retrieval outcome	Final PRISMA handling
Talseth 1988	Increasing incidence of PCP in renal transplant patients	No abstract or full report retrieved	Reports not retrieved
Rodriguez P 1998	Long-term corticosteroids and TMP-SMX prophylaxis	No readable abstract or full report retrieved	Reports not retrieved
Osama 2012	Dapsone as second-line PJP prophylaxis	Only title/citation retrieved	Reports not retrieved
Rajendran 2014	Late PJP after renal transplantation	Target report not retrieved; uploaded file was unrelated	Reports not retrieved
Keogh 1992	Mini-dose TMP-SMX after heart transplantation	Original report not retrieved; secondary descriptions not substituted	Reports not retrieved

Abbreviations: A-E, primary comparative effectiveness analysis; A-S, active-regimen safety analysis; TMP-SMX, trimethoprim-sulfamethoxazole.

Supplementary Table S3. Eligibility criteria and analysis-route decision framework.

Decision component	Eligibility or handling rule	Required extractable data	Final analytical handling
Common inclusion criteria	Adult, non-HIV, non-malignant, non-HSCT, non-primary-immunodeficiency immunocompromised populations, including rheumatic/autoimmune diseases, solid-organ transplantation, or long-term corticosteroid/immunosuppressant exposure. Studies had to evaluate PCP prophylaxis or an eligible no-prophylaxis comparator.	Population, prophylaxis context, prophylactic regimen or comparator, and at least one route-relevant denominator and event count.	Eligible records were assigned to one or more routes according to available outcome data.

Decision component	Eligibility or handling rule	Required extractable data	Final analytical handling
Common exclusion criteria	HIV infection; malignancy or chemotherapy-dominated populations; HSCT; primary immunodeficiency; paediatric-only data without separable adults; non-separable mixed populations; PCP treatment, diagnosis-only, mechanism-only, or risk-factor-only studies; reviews, protocols without results, and non-original reports.	None sufficient for the prespecified prophylaxis analyses.	Excluded at full-text/report assessment with harmonized exclusion category.
Route A-E: primary comparative effectiveness	Comparative evidence for PCP prophylaxis effectiveness across eligible nodes: TMP-SMX standard dose, TMP-SMX low/reduced dose, pentamidine, atovaquone where appropriate for sensitivity, dapsone if estimable, and no prophylaxis/observation/placebo.	Per-arm participants and PCP incidence events for at least two eligible arms or a usable comparator contrast.	Included in comparative effectiveness NMA if connected and clinically admissible; single-arm studies were not eligible for A-E.
Route A-S: active-regimen safety	Active-comparator evidence comparing low/reduced-dose or reduced-strategy TMP-SMX with standard/conventional TMP-SMX for discontinuation due to adverse events/reactions/toxicity.	Per-arm TMP-SMX participants and discontinuation events due to adverse events, adverse reactions, or toxicity.	Included in active-regimen pairwise safety analysis; no-prophylaxis, observation, or placebo arms were excluded because there was no prophylactic drug to discontinue.
Route B: TMP-SMX discontinuation burden	TMP-SMX prophylaxis arm or cohort with identifiable disease, population, dose, or clinical subgroup. A comparator was not required.	TMP-SMX denominator and number of discontinuations due to adverse events/reactions/toxicity.	Included in single-arm proportion meta-analysis and subgroup summaries; used to describe discontinuation burden, not causal comparative safety.
Route C: second-line prophylaxis after TMP-SMX intolerance/discontinuation	Atovaquone, dapsone, or aerosolized pentamidine used explicitly because of TMP-SMX intolerance, contraindication, allergy, toxicity, or discontinuation.	Second-line denominator plus breakthrough PCP events and/or discontinuation or toxicity events for the second-line agent.	Included as descriptive second-line evidence only; not used to rank second-line agents or infer equivalence to TMP-SMX.
Single-arm study handling	Single-arm or descriptive studies were not eligible for the main comparative NMA unless they provided a valid comparator contrast.	Route-specific single-arm denominators and events.	Eligible for Route B or C where prespecified data were available; otherwise excluded.
No-prophylaxis arm handling	No prophylaxis, observation, or placebo was an eligible comparator for effectiveness but not an active regimen.	For A-E, per-arm participants and PCP events. For safety/discontinuation, not applicable.	Eligible for A-E; excluded from A-S and B discontinuation analyses.
Duplicate or overlapping cohorts	Multiple reports from the same cohort, registry, trial, or conference/full-text sequence were not counted independently.	Most complete or most clinically informative data source; overlap notes where needed.	Merged under a single study-level record or excluded as duplicate/preliminary to avoid double counting.
Conference abstracts and registry records	All conference abstracts were assessed for a corresponding full-length peer-reviewed report. Abstract-only reports were not eligible for the quantitative evidence set.	For a corresponding full report: route-specific population, participants, events, and sufficient methodological detail. Abstract-only data were not accepted for any quantitative analysis.	A corresponding eligible full report was assessed preferentially and used as the sole quantitative source. If no eligible full report was identified, the abstract was listed among reports excluded after eligibility assessment in Supplementary Table S2 and was excluded from the base case and all quantitative sensitivity analyses.
Dapsone and other prespecified but non-estimable nodes	Dapsone was prespecified as a candidate node but lacked sufficient connected comparative evidence for the base-case effectiveness network.	Comparative per-arm PCP events for NMA, or second-line denominators/events for Route C.	Not included as an A-E base-case node; retained where appropriate in Route C descriptive second-line analyses.

Decision component	Eligibility or handling rule	Required extractable data	Final analytical handling
Atovaquone second-line and sensitivity-node handling	Atovaquone evidence was often dominated by second-line use after TMP-SMX intolerance or discontinuation, creating channeling/indication concerns for primary effectiveness comparisons.	Connected comparative data for sensitivity analyses and/or second-line denominator and breakthrough PCP events.	Excluded from the A-E base-case network; used only as a sensitivity node when connected and as descriptive Route C evidence when used after TMP-SMX intolerance/discontinuation.
Dose classification	Standard/conventional TMP-SMX included daily single-strength, daily double-strength, double-strength three-times-weekly, or renal-adjusted conventional equivalents. Low/reduced dose required explicit lower-dose, half-dose, very-low-dose, or dose-escalation strategy designation.	Dose category at arm level, plus route-specific denominator and event count.	Used for node assignment in A-E/A-S and for dose-stratified summaries in Route B. Unspecified dose was not automatically classified as low dose.

Note. A single study record could contribute to more than one route if route-specific denominators and events were extractable.

Abbreviations: A-E, primary comparative effectiveness analysis; A-S, active-regimen safety analysis; AE, adverse event; HSCT, haematopoietic stem-cell transplantation; NMA, network meta-analysis; TMP-SMX, trimethoprim-sulfamethoxazole.

Section S2. Study characteristics

Supplementary Table S4. Detailed characteristics of included study records.

Supplementary Table S4 is presented in two linked panels. Panel A retains the core study and analytical characteristics. Panel B reports, for all 54 included records, prophylaxis duration, follow-up, baseline immunosuppressive therapy or clinical context, and PCP diagnostic or ascertainment criteria wherever available. NR indicates that the item was not reported in the source; no missing characteristic was inferred or imputed.

Panel A. Core study and analytical characteristics.

Study record	Country/ region	Study design	Population domain and clinical population	Prophylaxis regimen arms	Comparator/control	Contributed route(s)	Route-specific sample size/events	Key notes
Higgins 1989[1]	NR	Retrospective/historical non-randomized cohort	Solid organ transplant: kidney transplant; kidney/renal transplant	TMP-SMX low dose; daily low-dose cotrimoxazole; No prophylaxis/observation/placebo	No prophylaxis/observation/placebo	A-E; B	A-E: 4/100 PCP events; B: 8/61 TMP-SMX discontinuations due to adverse events	Post-transplant prophylaxis
Elinder 1992[2]	Sweden	Retrospective cohort or registry	Solid organ transplant: kidney/renal transplant	TMP-SMX low dose; No prophylaxis/observation/placebo	No prophylaxis/observation/placebo	A-E	A-E: 8/375 PCP events	1st year after transplantation
Olsen 1993[3]	USA	Randomized/pilot randomized trial	Solid organ transplant: heart transplant; kidney/renal transplant	TMP-SMX standard dose; daily TMP-SMX; intermittent TMP-SMX; No prophylaxis/observation/placebo	No prophylaxis/observation/placebo	A-E; B	A-E: 7/56 PCP events; B: 0/39 TMP-SMX discontinuations due to adverse events	TMP-SMX started 14 days after transplantation and continued for 4 months
Saukkonen 1996[4]	USA	Retrospective cohort or registry	Solid organ transplant: solid organ transplant	pentamidine	NA	C	C: 0/35 breakthrough PCP events	Liver transplant recipients AP average 4.28±1.6 months; renal transplant recipients AP average 5.71±4.3 months
Torre-Cisneros 1996[5]	Spain	Retrospective cohort or registry; open clinical trial with historical control	Solid organ transplant: liver transplant	TMP-SMX low dose; daily low-dose cotrimoxazole 480 mg once daily; No prophylaxis/observation/placebo	No prophylaxis/observation/placebo	A-E; B	A-E: 4/70 PCP events; B: 2/60 TMP-SMX discontinuations due to adverse events (Demographic details were not available in the extracted full-text source.)	Cotrimoxazole 480 mg once daily for 6 months; 1-year mortality mentioned

Study record	Country/ region	Study design	Population domain and clinical population	Prophylaxis regimen arms	Comparator/control	Contributed route(s)	Route-specific sample size/events	Key notes
Okada 1999[6]	Japan	Retrospective cohort or registry	Rheumatic/autoimmune disease: CTD; other eligible immunocompromised	ST one tablet/day; ST two tablets/day; TMP-SMX low dose; TMP-SMX low/reduced dose; TMP-SMX standard dose; TMP-SMX standard/conventional dose; No prophylaxis/observation/placebo	No prophylaxis/observation/placebo	A-E; A-S; B	A-E: 2/84 PCP events; A-S: 12/37 discontinuations due to adverse events; B: 12/37 TMP-SMX discontinuations due to adverse events	ST duration: 1-tablet group 46.6±30.2 days; 2-tablet group 34.8±31.4 days
Torre-Cisneros 1999[7]	Spain	Randomized/pilot randomized trial	Solid organ transplant: liver transplant	daily low-dose TMP-SMX	Active comparator only	B	B: 3/60 TMP-SMX discontinuations due to adverse events	Study drugs were given during 6 months after transplantation
Meyers 2001[8]	USA	Prospective/QI cohort	Solid organ transplant: solid organ transplant	atovaquone	NA	C	C: 0/28 breakthrough PCP events	Atovaquone suspension once daily for 1 year; no PCP during study period or subsequent year
Ogawa 2005[9]	Japan	Retrospective cohort or registry	Rheumatic/autoimmune disease: rheumatic/autoimmune disease	TMP-SMX standard dose; No prophylaxis/observation/placebo	No prophylaxis/observation/placebo	A-E	A-E: 9/124 PCP events	At least 6 months after institution of prednisolone
Soejima 2007[10]	Japan	Retrospective cohort or registry	Rheumatic/autoimmune disease: SLE	prophylactic co-trimoxazole	NA	B	B: 2/54 TMP-SMX discontinuations due to adverse events	Hospitalization/prophylaxis period
Enomoto 2008[11]	Japan	Retrospective cohort or registry	Rheumatic/autoimmune disease: rheumatic/autoimmune disease	TMP-SMX standard dose; No prophylaxis/observation/placebo	No prophylaxis/observation/placebo	A-E	A-E: 7/64 PCP events	During glucocorticoid therapy; PCP developed after mean 71 days of glucocorticoid therapy among PCP cases
Kimura 2008[12]	Japan	Retrospective cohort or registry	Rheumatic/autoimmune disease: other eligible immunocompromised	TMP-SMX standard dose; pentamidine	Active comparator only	A-E	A-E: 3/45 PCP events	During prophylaxis/hospitalization period
Zhu 2010[13]	China	Randomized/pilot randomized trial	Solid organ transplant: kidney transplant; kidney/renal transplant	SMZ-TMP prophylaxis two tablets daily for 1 month; TMP-SMX standard dose; No prophylaxis/observation/placebo	No prophylaxis/observation/placebo	A-E; B	A-E: 1/20 PCP events; B: 1/10 TMP-SMX discontinuations due to adverse events	1 year after operation
Vananuvat 2011[14]	Thailand	Retrospective cohort or registry	Rheumatic/autoimmune disease: other eligible immunocompromised	TMP-SMX standard dose; No prophylaxis/observation/placebo	No prophylaxis/observation/placebo	A-E	A-E: 6/138 PCP events	At least 12 weeks after receiving qualifying corticosteroid dose
Chen Tongqing 2011[15]	China	Retrospective cohort or registry	Solid organ transplant: kidney/renal transplant	TMP-SMX low dose; No prophylaxis/observation/placebo	No prophylaxis/observation/placebo	A-E	A-E: 3/125 PCP events	SMZco for half a year to one year

Study record	Country/ region	Study design	Population domain and clinical population	Prophylaxis regimen arms	Comparator/control	Contributed route(s)	Route-specific sample size/events	Key notes
Gabardi 2012[16]	USA	Retrospective cohort or registry	Solid organ transplant: solid organ transplant	atovaquone	NA	C	C: 0/25 breakthrough PCP events	12 months
Liu 2012[17]	China	Retrospective cohort or registry	Solid organ transplant: kidney/renal transplant	TMP-SMX low dose; No prophylaxis/observation/placebo	No prophylaxis/observation/placebo	A-E	A-E: 9/190 PCP events	NR
Maezawa 2012[18]	Japan	Retrospective cohort or registry	Rheumatic/autoimmune disease: CTD plus pulmonary disease patients	prophylactic TMP-SMX	NA	B	B: 28/539 TMP-SMX discontinuations due to adverse events	NR
Takenaka 2013[19]	Japan	Retrospective cohort or registry	Rheumatic/autoimmune disease: rheumatic disease; rheumatic/autoimmune disease	TMP-SMX low dose; TMP-SMX low/reduced dose; TMP-SMX standard dose; TMP-SMX standard/conventional dose; dose-escalation TMP-SMX; routine TMP-SMX	Active comparator only	A-E; A-S; B	A-E: 1/41 PCP events; A-S: 5/41 discontinuations due to adverse events; B: 5/41 TMP-SMX discontinuations due to adverse events	Prophylaxis period
Katsuyama 2014[20]	Japan	Prospective/QI cohort; Retrospective cohort or registry	Rheumatic/autoimmune disease: RA receiving biologics; rheumatic/autoimmune	TMP-SMX prophylaxis; pentamidine	NA	B; C	B: 11/82 TMP-SMX discontinuations due to adverse events; C: 0/23 breakthrough PCP events (C denominator includes 12 initial aerosolized pentamidine recipients and 11 patients switched after TMP-SMX adverse events; strict switch-only sensitivity was 0/11.)	Mean observation period 5.7 months in second cohort
Mitsides 2014[21]	UK	Retrospective cohort or registry	Solid organ transplant: solid organ transplant	dapsone	NA	C	C: 0/26 breakthrough PCP events	During outbreak-response prophylaxis period
Mitsides 2014[22]	UK	Retrospective cohort or registry	Solid organ transplant: kidney transplant	TMP-SMX prophylaxis	NA	B	B: 110/290 TMP-SMX discontinuations due to adverse events	Post-transplant prophylaxis
Yamamoto 2014[23]	Japan	Randomized/pilot randomized trial	Rheumatic/autoimmune disease: rheumatic/autoimmune disease; systemic autoimmune disease	TMP-SMX low dose; TMP-SMX low/reduced dose; TMP-SMX standard dose; TMP-SMX standard/conventional dose; daily TMP-SMX; twice-weekly TMP-SMX	Active comparator only	A-E; A-S; B	A-E: 0/37 PCP events; A-S: 13/35 discontinuations due to adverse events; B: 13/35 TMP-SMX discontinuations due to adverse events	Up to 1 year
Evans 2015[24]	USA	Retrospective cohort or registry	Solid organ transplant: solid organ transplant	dapsone	NA	C	C: 0/79 breakthrough PCP events	6 and 12 months noted in route C extraction

Study record	Country/ region	Study design	Population domain and clinical population	Prophylaxis regimen arms	Comparator/control	Contributed route(s)	Route-specific sample size/events	Key notes
Macesic 2016[25]	Australia	Retrospective cohort or registry	Solid organ transplant: solid organ transplant	pentamidine	NA	C	C: 0/56 breakthrough PCP events	during prophylaxis
McLaughlin 2017[26]	USA	Retrospective cohort or registry	Solid organ transplant: kidney transplant	initial TMP-SMX prophylaxis	NA	B	B: 31/155 TMP-SMX discontinuations due to adverse events	NR
Utsunomiya 2017[27]	Japan	Randomized/pilot randomized trial	Rheumatic/autoimmune disease: rheumatic/autoimmune disease; systemic rheumatic disease	ES escalation TMP-SMX; HS half-strength TMP-SMX; SS standard TMP-SMX; TMP-SMX low dose; TMP-SMX low/reduced dose; TMP-SMX standard dose; TMP-SMX standard/conventional dose	Active comparator only	A-E; A-S; B	A-E: 0/172 PCP events; A-S: 22/172 discontinuations due to adverse events; B: 22/172 TMP-SMX discontinuations due to adverse events	24 weeks
Park 2018[28]	South Korea	Retrospective cohort or registry	Rheumatic/autoimmune disease: rheumatic/autoimmune disease	TMP-SMX standard dose; No prophylaxis/observation/placebo	No prophylaxis/observation/placebo	A-E	A-E: 30/1522 PCP events	1 year from baseline date for each treatment episode
Urbancic 2018[29]	Australia	Prospective/QI cohort	Solid organ transplant: kidney transplant	TMP-SMX prophylaxis with ADR/rechallenge protocol	NA	B	B: 42/119 TMP-SMX discontinuations due to adverse events	NR
Chapman 2019[30]	UK	Retrospective cohort or registry	Solid organ transplant: kidney transplant	TMP-SMX 3 vs 6 month protocol	NA	B	B: 18/580 TMP-SMX discontinuations due to adverse events	3 vs 6 months prophylaxis protocol
Kitazawa 2019[31]	Japan	Retrospective cohort or registry	Rheumatic/autoimmune disease: CTD; rheumatic/autoimmune	atovaquone; initial TMP-SMX then switch if AE; pentamidine	NA	B; C	B: 41/96 TMP-SMX discontinuations due to adverse events; C: 0/40 breakthrough PCP events	during prophylaxis; During corticosteroid therapy/hospitalized CTD cohort
Park 2019[32]	South Korea	Retrospective cohort or registry	Rheumatic/autoimmune disease: rheumatic disease with non-high-dose steroids; rheumatic/autoimmune disease	TMP-SMX prophylaxis episodes; TMP-SMX standard dose; No prophylaxis/observation/placebo	No prophylaxis/observation/placebo	A-E; B	A-E: 5/1065 PCP events; B: 9/45 TMP-SMX discontinuations due to adverse events	1-year risk period/treatment episode follow-up
Prasad 2019[33]	Canada	Retrospective cohort or registry	Solid organ transplant: kidney transplant	daily single-strength TMP-SMX with dose reductions	NA	B	B: 19/438 TMP-SMX discontinuations due to adverse events	12 months post-transplantation
Yamanaga 2020[34]	Japan	Retrospective cohort or registry	Solid organ transplant: kidney transplant	very-low-dose TMP-SMX re-administration	Active comparator only	B	B: 0/51 TMP-SMX discontinuations due to adverse events	re-administration period

Study record	Country/ region	Study design	Population domain and clinical population	Prophylaxis regimen arms	Comparator/control	Contributed route(s)	Route-specific sample size/events	Key notes
Chan 2021[35]	Hong Kong, China	Retrospective cohort or registry	Rheumatic/autoimmune disease: rheumatic/autoimmune disease	TMP-SMX standard dose; No prophylaxis/observation/placebo; pentamidine	No prophylaxis/observation/placebo	A-E	A-E: 48/21539 PCP events	1-year PCP risk assessed in analysis
Havele 2021[36]	USA	Retrospective cohort or registry	Rheumatic/autoimmune disease: chronic inflammatory/autoimmune disease with methotrexate	TMP-SMX DS 800/160 mg three times weekly with methotrexate	NA	B	B: 3/100 TMP-SMX discontinuations due to adverse events	NR
Honda 2021[37]	Japan	Retrospective cohort or registry	Rheumatic/autoimmune disease: rheumatic diseases on high-dose glucocorticoids; rheumatic/autoimmune disease	TMP-SMX prophylaxis; TMP-SMX standard dose; No prophylaxis/observation/placebo	No prophylaxis/observation/placebo	A-E; B	A-E: 3/437 PCP events; B: 70/376 TMP-SMX discontinuations due to adverse events	4 months from high-dose glucocorticoid start
Izuka 2021[38]	Japan	Retrospective cohort or registry	Rheumatic/autoimmune disease: CTD; SLE subgroup available	prophylactic TMP-SMX	NA	B	B: 40/424 TMP-SMX discontinuations due to adverse events	NR
Peterson 2021[39]	Canada	Prospective/QI cohort	Solid organ transplant: kidney transplant	lifelong universal TMP-SMX prophylaxis	NA	B	B: 3/237 TMP-SMX discontinuations due to adverse events	18 months after implementation; lifelong prophylaxis policy
Yang 2021[40]	China	Prospective/QI cohort	Solid organ transplant: kidney transplant	TWDS or DSS TMP-SMX	NA	B	B: 4/189 TMP-SMX discontinuations due to adverse events	Two-year prospective cohort study
Chen 2022[41]	China	Retrospective cohort or registry	Solid organ transplant: kidney/renal transplant	TMP-SMX low dose; No prophylaxis/observation/placebo	No prophylaxis/observation/placebo	A-E	A-E: 29/1469 PCP events	NR
Faraji 2022[42]	Iran	Retrospective cohort or registry	Rheumatic/autoimmune disease: pemphigus receiving rituximab; rheumatic/autoimmune disease	TMP-SMX standard dose; cotrimoxazole prophylaxis; No prophylaxis/observation/placebo	No prophylaxis/observation/placebo	A-E; B	A-E: 2/494 PCP events; B: 0/235 TMP-SMX discontinuations due to adverse events	NR

Study record	Country/ region	Study design	Population domain and clinical population	Prophylaxis regimen arms	Comparator/control	Contributed route(s)	Route-specific sample size/events	Key notes
Jinno 2022[43]	Japan	Retrospective cohort or registry	Rheumatic/autoimmune disease: CTD on high-dose glucocorticoids; rheumatic/autoimmune	TMP-SMX prophylaxis; atovaquone	NA	B; C	B: 102/383 TMP-SMX discontinuations due to adverse events; C: 5/107 breakthrough PCP events (Atovaquone denominator includes 95 patients switched after TMP-SMX adverse events and 12 initial atovaquone recipients.)	1 year from baseline
Park 2022[44]	South Korea	Retrospective cohort or registry	Solid organ transplant: solid organ transplant mixed	TMP-SMX standard dose; No prophylaxis/observation/placebo	No prophylaxis/observation/placebo	A-E	A-E: 28/1024 PCP events	Time-varying exposure analysis; exact fixed follow-up NR
Sonomoto 2022[45]	Japan	Retrospective cohort or registry	Rheumatic/autoimmune disease: rheumatic/autoimmune disease	TMP-SMX standard dose; No prophylaxis/observation/placebo; pentamidine	No prophylaxis/observation/placebo	A-E	A-E: 23/3781 PCP events	NR
Park 2023[46]	South Korea	Retrospective cohort or registry	Rheumatic/autoimmune disease: rheumatic/autoimmune disease	TMP-SMX standard dose; No prophylaxis/observation/placebo	No prophylaxis/observation/placebo	A-E	A-E: 11/818 PCP events	1-year risk window as reported in analysis context
Hiramoto 2024[47]	Japan	Retrospective cohort or registry	Rheumatic/autoimmune disease: rheumatic and musculoskeletal diseases	SMX/TMP prophylaxis; mostly one tablet or half tablet per day	NA	B	B: 82/438 TMP-SMX discontinuations due to adverse events	NR
Munthananu-chat 2024[48]	Thailand	Randomized/pilot randomized trial	Rheumatic/autoimmune disease: SLE; rheumatic/autoimmune disease	TMP-SMX standard dose; eGFR-adjusted TMP/SMX regimen; No prophylaxis/observation/placebo	No prophylaxis/observation/placebo	A-E; B	A-E: 0/138 PCP events; B: 10/70 TMP-SMX discontinuations due to adverse events	12 months
Ohmura 2024[49]	Japan	Retrospective cohort or registry	Rheumatic/autoimmune disease: rheumatic/autoimmune disease; systemic rheumatic disease	TMP-SMX low dose; TMP-SMX low/reduced dose; TMP-SMX standard dose; TMP-SMX standard/conventional dose; conventional-dose SMX/TMP one tablet/day; low-dose SMX/TMP one tablet twice weekly	Active comparator only	A-E; A-S; B	A-E: 0/186 PCP events; A-S: 43/186 discontinuations due to adverse events; B: 43/186 TMP-SMX discontinuations due to adverse events	1 year

Study record	Country/ region	Study design	Population domain and clinical population	Prophylaxis regimen arms	Comparator/control	Contributed route(s)	Route-specific sample size/events	Key notes
Aggoun 2025[50]	France	Retrospective cohort or registry	Solid organ transplant: heart transplant	TMP-SMX low dose; TMP-SMX low/reduced dose; TMP-SMX standard dose; TMP-SMX standard/conventional dose; low-dose SXT 20/100 mg; standard-dose SXT 80/400 mg	Active comparator only	A-E; A-S; B	A-E: 0/359 PCP events; A-S: 42/359 discontinuations due to adverse events; B: 42/359 TMP-SMX discontinuations due to adverse events (Two low-dose SXT arm PCP cases occurred during prophylaxis interruption and were not counted as events during ongoing prophylaxis.)	2-year follow-up after heart transplantation
He 2025[51]	USA	Retrospective cohort or registry	Solid organ transplant: solid organ transplant	atovaquone; dapsone	NA	C	C: 0/333 breakthrough PCP events	>1 year
Hudson/Tinkham 2025[52]	USA	Retrospective cohort or registry	Solid organ transplant: abdominal SOT; solid organ transplant	TMP/SMX prophylaxis after abdominal SOT; atovaquone; dapsone; pentamidine	NA	B; C	B: 184/930 TMP-SMX discontinuations due to adverse events; C: 0/129 breakthrough PCP events (Per-agent second-line PCP events were inferred from the overall TMP-SMX discontinuation group.)	180-day / 6-month prophylaxis window
Munroe 2025[53]	USA	Retrospective cohort or registry	Solid organ transplant: lung transplant	SMX/TMP single-strength twice weekly	NA	B	B: 23/105 TMP-SMX discontinuations due to adverse events	Indefinite prophylaxis regimen; study follow-up from transplant cohort review
Schumacher 2025[54]	USA	Retrospective single-center cohort	Solid organ transplant: kidney transplant	Dapsone 100 mg once weekly; SMX-TMP comparator	SMX-TMP	C	C: 0/175 breakthrough PCP in dapsone recipients	Dapsone used for sulfa allergy/intolerance; 509 total recipients (334 SMX-TMP; 175 dapsone).

Abbreviations: A-E, primary comparative effectiveness analysis; A-S, active-regimen safety analysis; ADR, adverse drug reaction; ATG, antithymocyte globulin; BAL, bronchoalveolar lavage; CTD, connective tissue disease; DFA, direct fluorescent antibody; HRCT, high-resolution computed tomography; MMF, mycophenolate mofetil; NA, not applicable; NR, not reported; PJP, source-reported abbreviation for the same condition; PCR, polymerase chain reaction; RA, rheumatoid arthritis; SLE, systemic lupus erythematosus; SOT, solid-organ transplant; TMP-SMX, trimethoprim-sulfamethoxazole.

Panel B. Clinically relevant heterogeneity variables reported by each included study.

Study record	Prophylaxis duration	Follow-up period	Baseline immunosuppressive therapy or clinical context	PCP diagnostic or ascertainment criteria
Higgins 1989[1]	480 mg daily for the first 6 months after renal transplantation.	PCP and safety outcomes assessed during the first 6 post-transplant months.	Renal transplant recipients; cyclosporine and prednisolone, with azathioprine in the prophylaxis cohort; antithymocyte globulin exposure occurred in some patients.	PCP was BAL-confirmed in the report; a fully prespecified diagnostic algorithm was not provided.
Elinder 1992[2]	Started 1–2 months after transplantation and continued until at least month 6.	First post-transplant year.	Kidney or kidney–pancreas transplantation; cyclosporine/prednisolone with variable azathioprine and anti-rejection methylprednisolone/RATG.	Suspected cases were confirmed by bronchoscopy/BAL with Pneumocystis antigen detection and histologic examination.
Olsen 1993[3]	Began on day 14 and continued for 4 months.	Primary follow-up 4 months; late PCP was described at 6–8 months.	Heart transplantation; OKT3 induction; maintenance corticosteroids, cyclosporine and azathioprine (occasionally cyclophosphamide).	Clinical screening (fever, imaging or hypoxemia) was followed by bronchoscopy/BAL; a positive silver stain was required.
Saukkonen 1996[4]	Monthly aerosolized pentamidine; mean 4.28 months in liver and 5.71 months in renal recipients.	Mean 23.4 months after transplantation.	Adult liver/kidney transplant recipients intolerant of TMP-SMX; detailed baseline regimen NR.	Organism documented by immunofluorescence or methenamine silver staining in induced sputum, BAL, transbronchial biopsy or open lung biopsy.
Torre-Cisneros 1996[5]	480 mg once daily for 6 months.	One year after liver transplantation.	Maintenance cyclosporine, steroids and azathioprine; rejection treated with methylprednisolone/increased prednisone.	Organism detected in BAL fluid or induced sputum.
Okada 1999[6]	Mean 46.6 ± 30.2 days (one-tablet group) and 34.8 ± 31.4 days (two-tablet group).	During corticosteroid treatment; fixed follow-up NR.	Autoimmune/CTD patients receiving corticosteroids, with additional immunosuppressive treatment recorded.	PCR or Grocott staining of sputum or BAL fluid was used to confirm PCP.
Torre-Cisneros 1999[7]	Study drugs for 6 months after liver transplantation.	Six-month trial period.	Maintenance cyclosporine, steroids and azathioprine; rejection treated with methylprednisolone/increased prednisone.	Pneumocystis organisms were identified in BAL or induced sputum (a monoclonal-antibody method was reported).
Meyers 2001[8]	Atovaquone once daily for 1 year.	Study year plus subsequent-year surveillance.	Orthotopic liver transplant recipients intolerant of TMP-SMX; detailed baseline regimen NR.	No breakthrough PCP; formal microbiologic case criteria NR.
Ogawa 2005[9]	During medium/high-dose corticosteroid treatment; fixed drug duration NR.	At least 6 months after starting prednisolone.	CTD patients receiving ≥30 mg/day prednisone; additional agents included cyclophosphamide, methotrexate and azathioprine.	Clinical-record ascertainment; complete microbiologic diagnostic algorithm NR.
Soejima 2007[10]	During prophylaxis/hospitalization; fixed duration NR.	NR.	SLE patients receiving co-trimoxazole prophylaxis; detailed baseline regimen NR.	Not applicable to analyzed safety outcome; criteria NR.

Study record	Prophylaxis duration	Follow-up period	Baseline immunosuppressive therapy or clinical context	PCP diagnostic or ascertainment criteria
Enomoto 2008[11]	During glucocorticoid therapy; fixed prophylaxis duration NR.	PCP cases occurred 38–175 days after starting glucocorticoids (mean 71 days).	Interstitial pneumonia treated with >0.5 mg/kg prednisolone for >3 weeks, with/without immunosuppressants including cyclosporine or cyclophosphamide.	Compatible HRCT plus Pneumocystis detected by microscopy or PCR in sputum/BAL; Papanicolaou, May–Giemsa and Grocott stains were reported.
Kimura 2008[12]	During indicated PCP prophylaxis; fixed duration NR.	Hospital/prophylaxis observation period; fixed follow-up NR.	CTD patients receiving >30 mg/day prednisolone and/or immunosuppressants with CD4 <300/μL.	Positive Pneumocystis PCR in sputum or BAL fluid.
Zhu 2010[13]	SMZ-TMP two tablets daily for 1 month.	One year after kidney transplantation.	Kidney transplant recipients; detailed induction/maintenance regimen NR in the report.	Clinical PCP outcome reported; detailed microbiologic criteria NR.
Vananuvat 2011[14]	During episodes of prednisone ≥20 mg/day; median high-dose exposure 3.8 months with prophylaxis.	At least 12 weeks after qualifying corticosteroid exposure.	CTD patients on prednisone ≥20 mg/day for >2 weeks; additional agents included cyclophosphamide, chlorambucil, cyclosporine, azathioprine, MMF and methotrexate.	Definite PCP: organism by immunofluorescence, modified Giemsa or methenamine silver in induced sputum/BAL; presumptive PCP allowed for compatible pneumonia responding to treatment.
Chen Tongqing 2011[15]	SMZco for 6–12 months.	Up to 1 year after kidney transplantation.	Early kidney transplant recipients receiving intensive immunosuppression; drug-level details NR.	Clinical PCP outcome reported; detailed microbiologic criteria NR.
Gabardi 2012[16]	Twelve months.	One-year PCP outcome.	Kidney transplant recipients; rATG or basiliximab induction, followed by tacrolimus, MMF and corticosteroids.	No breakthrough PCP; formal case criteria NR.
Liu 2012[17]	Started 1 month after transplantation and continued for 6 months.	One year after kidney transplantation.	Kidney transplant; methylprednisolone induction ± rabbit ATG; maintenance cyclosporine or tacrolimus plus MMF and prednisone.	Medical history, clinical manifestations, CT and laboratory assessment; organism-specific criteria NR.
Maezawa 2012[18]	During TMP-SMX prophylaxis; fixed duration NR.	NR.	CTD/pulmonary disease patients receiving >30 mg/day prednisolone for >4 weeks with/without immunosuppressants.	Not applicable to analyzed adverse-event outcome; criteria NR.
Takenaka 2013[19]	Six-month tolerability/retention assessment.	Six months.	Rheumatic disease patients receiving glucocorticoids with/without immunosuppressants, commonly tacrolimus or cyclophosphamide.	No prespecified organism-based criterion was reported for the prophylaxis outcome.
Katsuyama 2014[20]	During biologic treatment; changed to monthly pentamidine when TMP-SMX was stopped.	Mean 5.7 months in the prophylaxis cohort (first cohort mean 16.6 months).	RA patients receiving biologic agents, often with glucocorticoids and/or methotrexate.	Compatible symptoms/hypoxemia/interstitial imaging plus Pneumocystis PCR in sputum/BAL and/or elevated β-D-glucan.
Mitsides 2014[21]	Dapsone during the February–September 2011 outbreak response; individual duration varied.	Regular transplant follow-up; fixed duration NR.	Renal transplant recipients intolerant of TMP-SMX; heterogeneous maintenance immunosuppression including calcineurin inhibitors, MMF/azathioprine and steroids.	No breakthrough PCP during treatment; formal case criteria NR.

Study record	Prophylaxis duration	Follow-up period	Baseline immunosuppressive therapy or clinical context	PCP diagnostic or ascertainment criteria
Mitsides 2014[22]	Twelve months.	Approximately 12 months (mean total follow-up about 266–304 days by outcome group).	Established renal transplant recipients; mean two immunosuppressants, most on calcineurin-inhibitor therapy, with MMF/azathioprine/sirolimus variably used.	Clinical or radiographic picture consistent with PCP plus sputum PCR confirmation.
Yamamoto 2014[23]	Daily or twice-weekly regimen for up to 1 year.	One year.	Systemic autoimmune disease receiving medium/high-dose glucocorticoids.	Symptoms plus chest radiograph/CT; sputum PCR was a secondary endpoint.
Evans 2015[24]	Once-weekly dapsone during the recommended 6–12-month post-transplant window.	Outcomes assessed at 6 and 12 months.	Kidney/liver transplant recipients; induction and maintenance immunosuppression were reported, including ATG/basiliximab and standard transplant regimens.	No breakthrough PCP; formal case criteria NR.
Macesic 2016[25]	Universal prophylaxis intended for 1 year; aerosolized pentamidine median 5.8 months (overall prophylaxis median 15.8 months).	September 2012–May 2014.	Renal transplant recipients, some with rejection/CMV and ongoing corticosteroid exposure; full maintenance regimen NR.	No breakthrough PCP; formal case criteria NR.
McLaughlin 2017[26]	Per center protocol from transplantation; intended for at least 6 months; exact duration NR.	NR.	Kidney transplant recipients; detailed baseline regimen NR.	Not applicable to analyzed switching/tolerability outcome; criteria NR.
Utsunomiya 2017[27]	Twenty-four weeks in the current report (parent trial planned 52 weeks).	Twenty-four weeks.	Systemic rheumatic diseases starting prednisone ≥0.6 mg/kg/day, irrespective of concomitant immunosuppressants.	Suspected PJP was reviewed by a multidisciplinary clinical event committee; no cases occurred.
Park 2018[28]	Mean 237 days; generally initiated with high-dose steroids.	One year per treatment episode.	Rheumatic diseases receiving prolonged high-dose glucocorticoids; cyclophosphamide/steroid pulse and other immunosuppressants were captured.	Algorithm: positive PCR/DFA from induced sputum/BAL plus compatible clinical and radiographic findings; isolated PCR positivity was not counted.
Urbancic 2018[29]	During post-transplant protocol/rechallenge; fixed duration NR.	NR.	Renal transplant recipients under routine center immunosuppression; detailed regimen NR.	Not applicable to analyzed ADR/rechallenge outcome; criteria NR.
Chapman 2019[30]	Three- versus six-month protocol.	One year after kidney transplantation.	Kidney transplant recipients; mycophenolate exposure and post-transplant monitoring were recorded; full regimen NR.	No PCP events; formal case criteria NR.
Kitazawa 2019[31]	Until steroid/other immunosuppression was tapered at physician discretion.	Continuation assessed through 1 year.	Hospitalized CTD patients starting corticosteroids, with other immunosuppressants as clinically indicated.	Pneumocystis organisms were required in respiratory specimens.

Study record	Prophylaxis duration	Follow-up period	Baseline immunosuppressive therapy or clinical context	PCP diagnostic or ascertainment criteria
Park 2019[32]	During prolonged steroid treatment; prophylaxis duration varied.	One year per treatment episode.	Rheumatic diseases receiving prednisone <30 mg/day for ≥4 weeks; steroid pulse and cyclophosphamide were captured.	Algorithm: PCR/DFA in induced sputum/BAL plus compatible acute symptoms and radiographic findings.
Prasad 2019[33]	Daily single-strength prophylaxis prescribed for 12 months, with dose reductions as needed.	Twelve months after kidney transplantation.	Kidney transplant recipients; tacrolimus, mycophenolic acid and prednisone were the usual initial regimen.	No PCP events; formal case criteria NR.
Yamanaga 2020[34]	Very-low-dose re-administration period; fixed duration NR.	NR.	Kidney transplant outpatients on calcineurin-inhibitor/MMF-based maintenance therapy.	No PCP events; formal case criteria NR.
Chan 2021[35]	Prophylaxis exposure assessed within the cohort; fixed duration varied.	Five-year longitudinal data; 1-year risk was used in the analysis.	Rheumatic diseases; longitudinal glucocorticoid and other immunosuppressive treatment data were captured.	ICD-9 code and/or organism in deep respiratory specimens; microscopy/methenamine silver and quantitative PCR; asymptomatic PCR positivity was excluded.
Havele 2021[36]	Concurrent with methotrexate; fixed duration NR.	NR.	Chronic inflammatory/autoimmune disease on methotrexate, often with prednisone, biologics or other immunosuppressants.	No PCP events; formal case criteria NR.
Honda 2021[37]	During the 4-month high-dose glucocorticoid observation period.	Four months from high-dose glucocorticoid initiation.	Rheumatic diseases receiving high-dose glucocorticoids, sometimes pulse therapy and additional agents (cyclophosphamide, rituximab, calcineurin inhibitors, etc.).	PCR on BAL/sputum with clinical manifestations, compatible imaging and elevated β-D-glucan; organism microscopy was also recorded.
Izuka 2021[38]	During prophylaxis; fixed duration NR.	NR.	CTD, especially SLE, on high-dose prednisone (median 40 mg/day) with/without other immunosuppressants.	Not applicable to analyzed ADR outcome; criteria NR.
Peterson 2021[39]	Lifelong policy.	Eighteen months after implementation.	Kidney transplant recipients under routine maintenance immunosuppression; detailed regimen was not central to the report.	No new PJP; formal case criteria NR.
Yang 2021[40]	Six–12 months after kidney transplantation (study protocol).	Two years.	Kidney transplant recipients; contemporary immunosuppression, with the exact baseline regimen incompletely reported.	No PJP events; formal case criteria NR.
Chen 2022[41]	Daily or alternate-day low-dose prophylaxis during the first 180 days.	First 180 days after kidney transplantation.	Adult kidney transplant recipients; transplant immunosuppressive regimen was recorded but unevenly reported across study phases.	Compatible symptoms and chest imaging plus next-generation sequencing of BAL fluid or blood.
Faraji 2022[42]	During rituximab-associated prophylaxis; fixed duration NR.	NR.	Pemphigus treated with rituximab, with corticosteroid exposure captured.	PCP identified from medical records; complete organism-based criteria NR.
Jinno 2022[43]	Until glucocorticoids were tapered or the clinical risk period ended; duration varied.	One year from high-dose glucocorticoid initiation.	CTD receiving prednisone ≥20 mg/day for ≥4 weeks, with other immunosuppressants recorded.	Compatible clinical/radiographic syndrome; definitive if organisms were identified by sputum/BAL microscopy, and presumptive if β-D-glucan was elevated without microscopy.

Study record	Prophylaxis duration	Follow-up period	Baseline immunosuppressive therapy or clinical context	PCP diagnostic or ascertainment criteria
Park 2022[44]	TMP-SMX exposure modeled as time-varying; fixed duration varied.	One year after first rituximab exposure.	Rituximab-treated rheumatic or pre/post-SOT patients; concomitant high-dose steroids and other immunosuppressants were captured.	Two-step detection: positive DFA/PCR in induced sputum/BAL followed by blinded expert review of clinical and radiographic compatibility.
Sonomoto 2022[45]	During b/tsDMARD treatment; duration varied by prophylaxis strategy.	Long-term registry follow-up; fixed interval NR.	RA starting/switching biologic or targeted synthetic DMARDs, with concomitant glucocorticoid and lung-disease risk assessed.	Progressive hypoxemia/interstitial pneumonia plus elevated β-D-glucan and/or Pneumocystis PCR in sputum/BAL.
Park 2023[46]	TMP-SMX initiated around rituximab treatment; duration varied.	One year.	Rheumatic diseases receiving rituximab; prior/concomitant high-dose glucocorticoids, azathioprine and other immunosuppressants were captured.	Prespecified detection/adjudication was described in the source supplementary methods; the main report did not reproduce the complete criteria.
Hiramoto 2024[47]	During remission-induction therapy; fixed duration NR.	NR.	Rheumatic/musculoskeletal diseases receiving remission-induction regimens; glucocorticoids and other immunosuppressants were recorded.	Not applicable to analyzed ADR outcome; criteria NR.
Munthananuchat 2024[48]	Twelve months.	Twelve months.	SLE receiving low-level immunosuppression defined by prespecified dose/category thresholds.	Definite: histopathology or PCR plus compatible manifestations/imaging; probable: compatible manifestations/imaging and treatment response without organism evidence; colonization excluded.
Ohmura 2024[49]	Conventional or twice-weekly low dose during the study; assessed to 1 year.	One year.	Systemic rheumatic disease receiving moderate/high-dose glucocorticoids; prior immunosuppressants/biologics and pulse therapy were captured.	Clinical symptoms, hypoxemia and compatible imaging plus organism/PCR in a respiratory sample, or elevated β-D-glucan with treatment response.
Aggoun 2025[50]	Lifelong if tolerated.	Two years after heart transplantation.	ATG or basiliximab induction; corticosteroid, calcineurin-inhibitor and antimetabolite maintenance.	Clinical, radiologic and microbiologic workup; BAL microscopy with specified stains and P. jirovecii PCR; records/ICD screening was also used.
He 2025[51]	Kidney/kidney–pancreas: 180 days; liver: 1 year; lung: longer or indefinite; alternative prophylaxis was evaluated within the first year.	At least 1 year.	Adult multi-organ transplant recipients receiving alternative prophylaxis because of sulfa allergy or TMP-SMX toxicity; detailed regimens NR.	No PJP; infection was ascertained from electronic records; formal microbiologic criteria NR.
Hudson/Tinkham 2025[52]	180-day/6-month abdominal SOT window.	At least 180 days.	Abdominal SOT; basiliximab, alemtuzumab, ATG or steroid-only induction; maintenance-regimen details were not fully tabulated.	No PJP in the discontinuation group; electronic-record ascertainment, formal criteria NR.
Munroe 2025[53]	Indefinite twice-weekly regimen.	At least 6 months; median 4 years reported.	Lung transplant recipients under center maintenance immunosuppression; detailed regimen NR.	Positive DFA/PCR from sputum, bronchoscopic specimens, tracheal aspirate or lung tissue; treatment-initiated clinical cases were also counted.
Schumacher 2025[54]	Dapsone 100 mg once weekly; duration NR.	NR.	Kidney transplant recipients using dapsone for sulfa allergy/TMP-SMX intolerance; detailed baseline regimen NR.	Full report Table 2 reports PJP PCR positive in 0/175 patients; the broader case algorithm was NR.

Note. NR, not reported in the source report. When a study evaluated only treatment-limiting discontinuation or another safety outcome and did not ascertain PCP as an analyzed endpoint, the

diagnostic field is marked “Not applicable to analyzed outcome; criteria NR.” No unreported clinical characteristic was inferred or imputed.

Section S3. Risk of bias and evidence quality

Supplementary Table S5. Risk-of-bias and study-quality assessment of included studies.

Study	Route (s)	Assessment scope	Tool	Domain profile	Overall	Key concerns / rationale
Higgins 1989[1]	A-E; B	Comparative non-randomized outcome	ROBINS-I	D1:S; D2:S; D3:M; D4:M; D5:M; D6:L; D7:M	Serious	Full-text review corrected design: historical/consecutive records review after prophylaxis introduction, not randomized. Temporal confounding and selection bias are serious despite objective BAL-confirmed PCP diagnosis.
Higgins 1989	A-E; B	Single-arm/discontinuation or second-line outcome	Adapted JBI	Q1:Y; Q2:Y; Q3:Y; Q4:Y; Q5:U; Q6:Y	Moderate quality	Single-arm data estimate event burden only; no causal comparison.
Elinder 1992[2]	A-E	Comparative non-randomized outcome	ROBINS-I	D1:S; D2:M; D3:M; D4:M; D5:M; D6:M; D7:M	Serious	Non-randomized historical transplant cohort vulnerable to confounding by period/indication. Full text gives case table and BAL context, but no fully prespecified diagnostic algorithm.
Olsen 1993[3]	A-E; B	Comparative RCT/pilot randomized outcome	RoB 2	D1:SC; D2:SC; D3:L; D4:L; D5:SC	Some concerns	Bronchoscopic/silver-stain confirmation supports outcome measurement, but older open-label/pilot trial and lack of clearly available protocol create some concerns.
Olsen 1993	A-E; B	Single-arm/discontinuation or second-line outcome	Adapted JBI	Q1:Y; Q2:Y; Q3:Y; Q4:Y; Q5:U; Q6:Y	Moderate quality	Single-arm data estimate event burden only; no causal comparison.
Saukkonen 1996[4]	C	Single-arm/discontinuation or second-line outcome	Adapted JBI	Q1:Y; Q2:Y; Q3:U; Q4:Y; Q5:U; Q6:Y	Moderate quality	Single-arm data estimate event burden only; no causal comparison. Breakthrough definition partly unclear.
Torre-Cisneros 1996[5]	A-E; B	Comparative non-randomized outcome	ROBINS-I	D1:S; D2:S; D3:M; D4:M; D5:M; D6:M; D7:M	Serious	Non-randomized assignment; confounding by indication/risk selection. Historical controls further increase selection and temporal-confounding concerns.
Torre-Cisneros 1996	A-E; B	Single-arm/discontinuation or second-line outcome	Adapted JBI	Q1:Y; Q2:Y; Q3:Y; Q4:Y; Q5:U; Q6:Y	Moderate quality	Single-arm data estimate event burden only; no causal comparison.
Okada 1999[6]	A-E; A-S; B	Comparative non-randomized outcome	ROBINS-I	D1:S; D2:S; D3:M; D4:M; D5:S; D6:M; D7:M	Serious	Non-randomized assignment; confounding by indication/risk selection. Older retrospective safety/effectiveness record with short, unequal ST duration by dose group.
Okada 1999	A-E; A-S; B	Single-arm/discontinuation or second-line outcome	Adapted JBI	Q1:Y; Q2:Y; Q3:Y; Q4:Y; Q5:U; Q6:Y	Moderate quality	Single-arm data estimate event burden only; no causal comparison.

Study	Route (s)	Assessment scope	Tool	Domain profile	Overall	Key concerns / rationale
Torre-Cisneros 1999[7]	B	Comparative RCT/pilot randomized outcome	RoB 2	D1:SC; D2:SC; D3:SC; D4:SC; D5:SC	Some concerns	Full text verified: prospective randomized active-comparator trial; drugs given for 6 months; PCP diagnosis required organism in BAL or induced sputum. Randomization/concealment/blinding details remain insufficient.
Torre-Cisneros 1999	B	Single-arm/discontinuation or second-line outcome	Adapted JBI	Q1:Y; Q2:Y; Q3:Y; Q4:Y; Q5:U; Q6:Y	Moderate quality	Single-arm data estimate event burden only; no causal comparison.
Meyers 2001[8]	C	Single-arm/discontinuation or second-line outcome	Adapted JBI	Q1:Y; Q2:Y; Q3:U; Q4:Y; Q5:U; Q6:Y	Moderate quality	Single-arm data estimate event burden only; no causal comparison. Breakthrough definition partly unclear.
Ogawa 2005[9]	A-E	Comparative non-randomized outcome	ROBINS-I	D1:S; D2:M; D3:M; D4:M; D5:M; D6:S; D7:M	Serious	Non-randomized assignment; confounding by indication/risk selection. Outcome definition/adjudication partly unclear.
Soejima 2007[10]	B	Single-arm/discontinuation or second-line outcome	Adapted JBI	Q1:Y; Q2:Y; Q3:Y; Q4:Y; Q5:U; Q6:Y	Moderate quality	Single-arm data estimate event burden only; no causal comparison.
Enomoto 2008[11]	A-E	Comparative non-randomized outcome	ROBINS-I	D1:S; D2:M; D3:M; D4:M; D5:M; D6:M; D7:M	Serious	Non-randomized assignment; confounding by indication/risk selection.
Kimura 2008[12]	A-E	Comparative non-randomized outcome	ROBINS-I	D1:S; D2:M; D3:M; D4:M; D5:M; D6:S; D7:M	Serious	Non-randomized assignment; confounding by indication/risk selection. Active-dose or active-agent comparisons may reflect tolerability-driven dose selection or channeling. Outcome definition/adjudication partly unclear.…
Zhu 2010[13]	A-E; B	Comparative RCT/pilot randomized outcome	RoB 2	D1:H; D2:SC; D3:SC; D4:SC; D5:SC	High	Full text verified: Chinese study states random division into three groups, but random sequence generation, concealment, and blinding were not described; very small study.
Zhu 2010	A-E; B	Single-arm/discontinuation or second-line outcome	Adapted JBI	Q1:Y; Q2:Y; Q3:Y; Q4:Y; Q5:U; Q6:Y	Moderate quality	Single-arm data estimate event burden only; no causal comparison.
Vananuvat 2011[14]	A-E	Comparative non-randomized outcome	ROBINS-I	D1:S; D2:M; D3:M; D4:M; D5:M; D6:M; D7:M	Serious	Non-randomized assignment; confounding by indication/risk selection.
Chen Tongqing 2011[15]	A-E	Comparative non-randomized outcome	ROBINS-I	D1:S; D2:M; D3:M; D4:M; D5:M; D6:S; D7:M	Serious	Non-randomized assignment; confounding by indication/risk selection. Outcome definition/adjudication partly unclear. Older transplant cohort and incomplete diagnostic detail limit confidence.
Gabardi 2012[16]	C	Single-arm/discontinuation or second-line outcome	Adapted JBI	Q1:Y; Q2:Y; Q3:U; Q4:Y; Q5:U; Q6:Y	Moderate quality	Single-arm data estimate event burden only; no causal comparison. Breakthrough definition partly unclear.

Study	Route (s)	Assessment scope	Tool	Domain profile	Overall	Key concerns / rationale
Liu 2012[17]	A-E	Comparative non-randomized outcome	ROBINS-I	D1:S; D2:M; D3:M; D4:M; D5:S; D6:S; D7:M	Serious	Non-randomized assignment; confounding by indication/risk selection. Outcome definition/adjudication partly unclear. Follow-up/missing data partly NR. Older transplant cohort and incomplete diagnostic detail limit…
Maezawa 2012[18]	B	Single-arm/discontinuation or second-line outcome	Adapted JBI	Q1:Y; Q2:Y; Q3:P; Q4:U; Q5:U; Q6:Y	Moderate quality	Single-arm data estimate event burden only; no causal comparison. Follow-up duration or attrition handling was not fully reported. The outcome was adverse-event discontinuation rather than PCP diagnosis; adverse-event…
Takenaka 2013[19]	A-E; A-S; B	Comparative non-randomized outcome	ROBINS-I	D1:S; D2:M; D3:M; D4:M; D5:M; D6:S; D7:M	Serious	Non-randomized assignment; confounding by indication/risk selection. Active-dose or active-agent comparisons may reflect tolerability-driven dose selection or channeling. Outcome definition/adjudication partly unclear.…
Takenaka 2013	A-E; A-S; B	Single-arm/discontinuation or second-line outcome	Adapted JBI	Q1:Y; Q2:Y; Q3:Y; Q4:Y; Q5:U; Q6:Y	Moderate quality	Single-arm data estimate event burden only; no causal comparison.
Katsuyama 2014[20]	B; C	Single-arm/discontinuation or second-line outcome	Adapted JBI	Q1:Y; Q2:Y; Q3:Y; Q4:Y; Q5:U; Q6:P	Low-to-moderate quality	Single-arm data estimate event burden only; no causal comparison. Denominator mixed initial second-line use and switch after TMP-SMX intolerance/discontinuation.
Mitsides 2014[21]	C	Single-arm/discontinuation or second-line outcome	Adapted JBI	Q1:Y; Q2:Y; Q3:U; Q4:Y; Q5:U; Q6:Y	Moderate quality	Single-arm data estimate event burden only; no causal comparison. Breakthrough definition partly unclear.
Mitsides 2014[22]	B	Single-arm/discontinuation or second-line outcome	Adapted JBI	Q1:Y; Q2:Y; Q3:Y; Q4:Y; Q5:U; Q6:Y	Moderate quality	Single-arm data estimate event burden only; no causal comparison.
Yamamoto 2014[23]	A-E; A-S; B	Comparative RCT/pilot randomized outcome	RoB 2	D1:SC; D2:SC; D3:SC; D4:SC; D5:SC	Some concerns	Full text verified: randomized prospective feasibility study with random allocation; randomization/concealment details not described; safety discontinuation outcome may be influenced by open-label management.
Yamamoto 2014	A-E; A-S; B	Single-arm/discontinuation or second-line outcome	Adapted JBI	Q1:Y; Q2:Y; Q3:Y; Q4:Y; Q5:U; Q6:Y	Moderate quality	Single-arm data estimate event burden only; no causal comparison.
Evans 2015[24]	C	Single-arm/discontinuation or second-line outcome	Adapted JBI	Q1:Y; Q2:Y; Q3:U; Q4:Y; Q5:U; Q6:Y	Moderate quality	Single-arm data estimate event burden only; no causal comparison. Breakthrough definition partly unclear.
Macesic 2016[25]	C	Single-arm/discontinuation or second-line outcome	Adapted JBI	Q1:Y; Q2:Y; Q3:U; Q4:Y; Q5:U; Q6:Y	Moderate quality	Single-arm data estimate event burden only; no causal comparison. Breakthrough definition partly unclear.

Study	Route (s)	Assessment scope	Tool	Domain profile	Overall	Key concerns / rationale
McLaughlin 2017[26]	B	Single-arm/discontinuation or second-line outcome	Adapted JBI	Q1:Y; Q2:Y; Q3:Y; Q4:U; Q5:U; Q6:Y	Low-to-moderate quality	Single-arm data estimate event burden only; no causal comparison. Follow-up duration or attrition handling was not fully reported.
Utsunomiya 2017[27]	A-E; A-S; B	Comparative RCT/pilot randomized outcome	RoB 2	D1:SC; D2:SC; D3:L; D4:SC; D5:SC	Some concerns	Full text verified: multicenter open/non-blinded RCT with computer-based central dynamic allocation/block randomization; event review committee planned for PCP endpoint. Open-label design still creates some concerns…
Utsunomiya 2017	A-E; A-S; B	Single-arm/discontinuation or second-line outcome	Adapted JBI	Q1:Y; Q2:Y; Q3:Y; Q4:Y; Q5:U; Q6:Y	Moderate quality	Single-arm data estimate event burden only; no causal comparison.
Park 2018[28]	A-E	Comparative non-randomized outcome	ROBINS-I	D1:S; D2:M; D3:M; D4:M; D5:M; D6:M; D7:M	Serious	Non-randomized assignment; confounding by indication/risk selection. Algorithmic outcome capture/clinical review improves measurement, but residual confounding remains.
Urbancic 2018[29]	B	Single-arm/discontinuation or second-line outcome	Adapted JBI	Q1:Y; Q2:Y; Q3:Y; Q4:U; Q5:U; Q6:Y	Moderate quality	Single-arm data estimate event burden only; no causal comparison. Follow-up duration or attrition handling was not fully reported.
Chapman 2019[30]	B	Single-arm/discontinuation or second-line outcome	Adapted JBI	Q1:Y; Q2:Y; Q3:Y; Q4:Y; Q5:U; Q6:Y	Moderate quality	Single-arm data estimate event burden only; no causal comparison.
Kitazawa 2019[31]	B; C	Single-arm/discontinuation or second-line outcome	Adapted JBI	Q1:Y; Q2:Y; Q3:Y; Q4:Y; Q5:U; Q6:Y	Low-to-moderate quality	Single-arm data estimate event burden only; no causal comparison.
Park 2019[32]	A-E; B	Comparative non-randomized outcome	ROBINS-I	D1:S; D2:M; D3:M; D4:M; D5:M; D6:S; D7:M	Serious	Non-randomized assignment; confounding by indication/risk selection. Outcome definition/adjudication partly unclear.
Park 2019	A-E; B	Single-arm/discontinuation or second-line outcome	Adapted JBI	Q1:Y; Q2:Y; Q3:Y; Q4:Y; Q5:U; Q6:Y	Moderate quality	Single-arm data estimate event burden only; no causal comparison.
Prasad 2019[33]	B	Single-arm/discontinuation or second-line outcome	Adapted JBI	Q1:Y; Q2:Y; Q3:Y; Q4:Y; Q5:U; Q6:Y	Moderate quality	Single-arm data estimate event burden only; no causal comparison.
Yamanaga 2020[34]	B	Single-arm/discontinuation or second-line outcome	Adapted JBI	Q1:Y; Q2:Y; Q3:Y; Q4:Y; Q5:U; Q6:Y	Moderate quality	Single-arm data estimate event burden only; no causal comparison.
Chan 2021[35]	A-E	Comparative non-randomized outcome	ROBINS-I	D1:S; D2:M; D3:M; D4:M; D5:M; D6:S; D7:M	Serious	Non-randomized assignment; confounding by indication/risk selection. Outcome definition/adjudication partly unclear. Very large database study, but diagnostic/clinical adjudication details were not fully extractable in…

Study	Route (s)	Assessment scope	Tool	Domain profile	Overall	Key concerns / rationale
Havele 2021[36]	B	Single-arm/discontinuation or second-line outcome	Adapted JBI	Q1:Y; Q2:Y; Q3:P; Q4:U; Q5:U; Q6:Y	Moderate quality	Single-arm data estimate event burden only; no causal comparison. Follow-up duration or attrition handling was not fully reported. The outcome was adverse-event discontinuation rather than PCP diagnosis; adverse-event…
Honda 2021[37]	A-E; B	Comparative non-randomized outcome	ROBINS-I	D1:S; D2:M; D3:M; D4:M; D5:M; D6:S; D7:M	Serious	Non-randomized assignment; confounding by indication/risk selection. Outcome definition/adjudication partly unclear.
Honda 2021	A-E; B	Single-arm/discontinuation or second-line outcome	Adapted JBI	Q1:Y; Q2:Y; Q3:Y; Q4:Y; Q5:U; Q6:Y	Moderate quality	Single-arm data estimate event burden only; no causal comparison.
Izuka 2021[38]	B	Single-arm/discontinuation or second-line outcome	Adapted JBI	Q1:Y; Q2:Y; Q3:P; Q4:U; Q5:U; Q6:Y	Moderate quality	Single-arm data estimate event burden only; no causal comparison. Follow-up duration or attrition handling was not fully reported. The outcome was adverse-event discontinuation rather than PCP diagnosis; adverse-event…
Peterson 2021[39]	B	Single-arm/discontinuation or second-line outcome	Adapted JBI	Q1:Y; Q2:Y; Q3:Y; Q4:Y; Q5:U; Q6:Y	Moderate quality	Single-arm data estimate event burden only; no causal comparison.
Yang 2021[40]	B	Single-arm/discontinuation or second-line outcome	Adapted JBI	Q1:Y; Q2:Y; Q3:Y; Q4:Y; Q5:U; Q6:Y	Moderate quality	Single-arm data estimate event burden only; no causal comparison.
Chen 2022[41]	A-E	Comparative non-randomized outcome	ROBINS-I	D1:S; D2:M; D3:M; D4:M; D5:S; D6:S; D7:M	Serious	Non-randomized assignment; confounding by indication/risk selection. Outcome definition/adjudication partly unclear. Follow-up/missing data partly NR.
Faraji 2022[42]	A-E; B	Comparative non-randomized outcome	ROBINS-I	D1:S; D2:M; D3:M; D4:M; D5:S; D6:S; D7:M	Serious	Non-randomized assignment; confounding by indication/risk selection. Outcome definition/adjudication partly unclear. Follow-up/missing data partly NR. Sparse PCP events and incomplete follow-up/reporting details.
Faraji 2022	A-E; B	Single-arm/discontinuation or second-line outcome	Adapted JBI	Q1:Y; Q2:Y; Q3:Y; Q4:U; Q5:U; Q6:Y	Moderate quality	Single-arm data estimate event burden only; no causal comparison. Follow-up duration or attrition handling was not fully reported.
Jinno 2022[43]	B; C	Single-arm/discontinuation or second-line outcome	Adapted JBI	Q1:Y; Q2:Y; Q3:Y; Q4:Y; Q5:U; Q6:P	Low-to-moderate quality	Single-arm data estimate event burden only; no causal comparison. Denominator mixed initial second-line use and switch after TMP-SMX intolerance/discontinuation.
Park 2022[44]	A-E	Comparative non-randomized outcome	ROBINS-I	D1:S; D2:M; D3:M; D4:M; D5:M; D6:M; D7:M	Serious	Non-randomized assignment; confounding by indication/risk selection. Algorithmic outcome capture/clinical review improves measurement, but residual confounding remains.

Study	Route (s)	Assessment scope	Tool	Domain profile	Overall	Key concerns / rationale
Sonomoto 2022[45]	A-E	Comparative non-randomized outcome	ROBINS-I	D1:S; D2:M; D3:M; D4:M; D5:S; D6:S; D7:M	Serious	Non-randomized assignment; confounding by indication/risk selection. Outcome definition/adjudication partly unclear. Follow-up/missing data partly NR.
Park 2023[46]	A-E	Comparative non-randomized outcome	ROBINS-I	D1:S; D2:M; D3:M; D4:M; D5:M; D6:S; D7:M	Serious	Non-randomized assignment; confounding by indication/risk selection. Outcome definition/adjudication partly unclear.
Hiramoto 2024[47]	B	Single-arm/discontinuation or second-line outcome	Adapted JBI	Q1:Y; Q2:Y; Q3:P; Q4:U; Q5:U; Q6:Y	Moderate quality	Single-arm data estimate event burden only; no causal comparison. Follow-up duration or attrition handling was not fully reported. The outcome was adverse-event discontinuation rather than PCP diagnosis; adverse-event…
Munthananu-chat 2024[48]	A-E; B	Comparative RCT/pilot randomized outcome	RoB 2	D1:SC; D2:SC; D3:SC; D4:SC; D5:SC	Some concerns	Full text verified: open-label randomized controlled trial with 1:1 computer-generated block randomization; lack of blinding and sparse events lead to some concerns.
Munthananu-chat 2024	A-E; B	Single-arm/discontinuation or second-line outcome	Adapted JBI	Q1:Y; Q2:Y; Q3:Y; Q4:Y; Q5:U; Q6:Y	Moderate quality	Single-arm data estimate event burden only; no causal comparison.
Ohmura 2024[49]	A-E; A-S; B	Comparative non-randomized outcome	ROBINS-I	D1:S; D2:M; D3:L; D4:M; D5:M; D6:M; D7:M	Serious	Non-randomized assignment; confounding by indication/risk selection. Active-dose or active-agent comparisons may reflect tolerability-driven dose selection or channeling. Dose definitions are clear; nevertheless,…
Ohmura 2024	A-E; A-S; B	Single-arm/discontinuation or second-line outcome	Adapted JBI	Q1:Y; Q2:Y; Q3:Y; Q4:Y; Q5:U; Q6:Y	Moderate quality	Single-arm data estimate event burden only; no causal comparison.
Aggoun 2025[50]	A-E; A-S; B	Comparative non-randomized outcome	ROBINS-I	D1:S; D2:M; D3:L; D4:M; D5:M; D6:M; D7:M	Serious	Non-randomized assignment; confounding by indication/risk selection. Active-dose or active-agent comparisons may reflect tolerability-driven dose selection or channeling. Dose definitions are clear; nevertheless,…
Aggoun 2025	A-E; A-S; B	Single-arm/discontinuation or second-line outcome	Adapted JBI	Q1:Y; Q2:Y; Q3:Y; Q4:Y; Q5:U; Q6:Y	Moderate quality	Single-arm data estimate event burden only; no causal comparison.
He 2025[51]	C	Single-arm/discontinuation or second-line outcome	Adapted JBI	Q1:Y; Q2:Y; Q3:U; Q4:Y; Q5:U; Q6:Y	Moderate quality	Single-arm data estimate event burden only; no causal comparison. Breakthrough definition partly unclear.
Hudson 2025[52]	B; C	Single-arm/discontinuation or second-line outcome	Adapted JBI	Q1:Y; Q2:Y; Q3:U; Q4:Y; Q5:U; Q6:P	Low-to-moderate quality	Single-arm data estimate event burden only; no causal comparison. Breakthrough definition partly unclear. Per-agent second-line event counts were inferred from overall data and should be interpreted cautiously.
Munroe 2025[53]	B	Single-arm/discontinuation or second-line outcome	Adapted JBI	Q1:Y; Q2:Y; Q3:Y; Q4:Y; Q5:U; Q6:Y	Moderate quality	Single-arm data estimate event burden only; no causal comparison.

Study	Route (s)	Assessment scope	Tool	Domain profile	Overall	Key concerns / rationale
Schumacher 2025[54]	C	Single-arm/discontinuation or second-line outcome	Adapted JBI	Q1:Y; Q2:Y; Q3:Y; Q4:Y; Q5:U; Q6:Y	Moderate quality	Retrospective selected second-line cohort; appropriate for descriptive breakthrough and safety context, not comparative efficacy ranking.

Abbreviations used in domain profiles are defined in the notes to Supplementary Figures S1–5.

Supplementary Table S6. Risk-of-bias and study-quality assessment by analysis route and outcome.

Analysis route / outcome	Evidence base	RoB/quality tools	Dominant concerns	Effect on interpretation
A-E: primary comparative effectiveness / PCP incidence	27 comparative studies; base-case network linked no prophylaxis, TMP-SMX standard, TMP-SMX low dose, and pentamidine.	RoB 2 for randomized/pilot randomized studies; ROBINS-I for non-randomized comparative studies.	Most evidence was non-randomized. Key concerns were confounding by indication, clinician/patient risk selection, historical controls, and incomplete diagnostic/outcome reporting in older cohorts.	Supports a protective association for TMP-SMX versus no prophylaxis, but does not support definitive active-regimen ranking or claims of low-dose superiority.
A-S: active-regimen safety / discontinuation due to adverse events	6 active-regimen comparative studies comparing low/reduced-dose with standard/conventional-dose TMP-SMX.	RoB 2 for randomized/pilot randomized studies; ROBINS-I for non-randomized active-comparator studies.	Dose selection was often non-randomized and may track kidney function, frailty, expected intolerance, or clinician preference; open-label discontinuation decisions and adverse-event definitions varied.	Findings suggest lower discontinuation with low/reduced-dose TMP-SMX, but should be presented as a tolerability signal rather than conclusive causal superiority.
B: TMP-SMX discontinuation subgroup / single-arm discontinuation proportion	33 full-length peer-reviewed TMP-SMX studies/cohorts from randomized, retrospective, and prospective/QI designs.	Adapted JBI single-arm/cohort checklist.	Single-arm evidence cannot compare regimens. Representativeness was frequently unclear or not reported; follow-up and adverse-event discontinuation definitions varied.	Appropriate for quantifying discontinuation burden and clinical context, not for causal comparisons or treatment ranking.
C: second-line prophylaxis after TMP-SMX intolerance/discontinuation / breakthrough PCP and safety	12 full-length peer-reviewed reports describing atovaquone, dapsone, and/or pentamidine after TMP-SMX intolerance, contraindication, toxicity, or discontinuation.	Adapted JBI single-arm/descriptive checklist.	Mostly non-comparative and sparse event data; second-line populations are selected by TMP-SMX intolerance; some denominators mix initial second-line use with switch-after-intolerance patients; per-agent events were partly inferred in one report.	Use only as descriptive evidence on breakthrough events and tolerability. Do not include in primary active-regimen ranking or infer comparative efficacy.

Supplementary Table S7. Limited-information and unresolved reporting items.

Study/record	Issue	Why unresolved/limited	How handled	Impact on interpretation
Katsuyama 2014[20]	Mixed second-line denominator	Full text supports prophylaxis protocol but second-line pentamidine denominator mixes initial alternative prophylaxis and switch after TMP-SMX adverse events.	Footnote explicitly; retain strict switch-only sensitivity where possible.	Affects descriptive Route C.
Jinno 2022[43]	Mixed atovaquone denominator	Full text confirms patients switched after TMP-SMX discontinuation, but atovaquone denominator also includes initial atovaquone users.	Footnote and keep Route C descriptive; avoid comparative ranking.	Affects descriptive Route C.
Hudson 2025[52]	Per-agent second-line events inferred	Full text reports aggregate TMP/SMX discontinuation and alternative-agent switches; per-agent PCP outcomes are not fully separable.	Use only descriptively; sensitivity excluding inferred per-agent data.	Affects descriptive Route C.
Mitsides 2014 [21, 22]	Potential overlapping renal-transplant center/cohort context	Records address related prophylaxis safety experience but different extracted questions; exact patient overlap cannot be fully excluded from publication text.	Potential overlap was handled conservatively by ensuring that related records did not contribute duplicate data to the same analysis.	Handled as overlap-control note only.
Schumacher 2025[54]	PJP outcome directly reported in the full report	Schumacher 2025 Table 2 directly reports dapsone n=175 and positive PJP via polymerase chain reaction, 0 (0).	Use 0/175 directly from Schumacher 2025 Table 2. The 2023 ACCP abstract is a preliminary/overlapping report, excluded to avoid double counting, and is not the source of either value.	Affects descriptive Route C only; no comparative ranking.

Section S4. Outcome availability and analytical handling

Outcome availability and feasibility of quantitative synthesis were assessed after full-text extraction. This section documents how protocol-specified outcomes were mapped to final analyses, why some outcomes were not quantitatively synthesized, and how sparse-event modeling was handled transparently.

Supplementary Table S8. Outcome availability and feasibility of quantitative synthesis.

Protocol-specified outcome	Outcome category	Available extractable data	Feasible synthesis method	Final handling in this review	Reason if not quantitatively synthesized	Related route	Interpretation note
Confirmed or probable PCP incidence during prophylaxis or follow-up	Primary effectiveness outcome	Arm-level events and denominators were extractable for the primary comparative effectiveness dataset: 27 studies, 57 arms, 243/34,473 PCP events/participants.	Comparative network meta-analysis feasible for connected nodes with extractable arm-level data.	Synthesized in Route A-E. Base-case nodes were no prophylaxis/observation/placebo, TMP-SMX standard dose, TMP-SMX low dose, and pentamidine. Atovaquone was examined only as a sensitivity node because most available atovaquone data reflected second-line use after TMP-SMX intolerance/discontinuation. Dapsone was prespecified but did not provide a connected and sufficiently extractable comparative effectiveness dataset for the base-case NMA and was therefore not estimable in the primary network.	Not applicable for the base-case effectiveness outcome; however, active-regimen ranking should be conservative because much of the evidence was non-randomized and some active nodes were sparse or affected by channeling. The protocol specified risk ratios for binary outcomes; odds ratios were used in the final sparse-event NMA for model stability and compatibility with the implemented framework.	A-E	Primary efficacy conclusion should focus on the protective association of TMP-SMX versus no prophylaxis rather than definitive superiority among active regimens. The OR effect measure should be reported as a transparent analytical adaptation from the protocol-specified RR.
Discontinuation due to adverse events, adverse reactions, or toxicity	Primary safety/tolerability outcome	Comparative active-regimen data were extractable for 6 studies and 12 TMP-SMX arms in A-S (137/830 discontinuations/participants). Single-arm TMP-SMX discontinuation data were extractable for 33 independent studies/cohorts in Route B (983/6996 discontinuations/participants).	A connected active-regimen safety network across all agents was not feasible. A pairwise active-comparator synthesis was feasible for low/reduced-dose versus standard/conventional-dose TMP-SMX; single-arm proportion meta-analysis was feasible for TMP-SMX discontinuation burden.	Synthesized as Route A-S for the active TMP-SMX dose comparison and Route B for TMP-SMX discontinuation burden/subgroups.	Atovaquone, dapsone, and pentamidine safety data were mostly non-comparative or second-line-after-intolerance data, and discontinuation definitions varied across studies; single-arm evidence cannot rank regimens.	A-S; B; C descriptive safety	This is the main quantitative safety endpoint. The A-S result should be framed as a tolerability signal for low/reduced-dose TMP-SMX, not proof of causal superiority.

Protocol-specified outcome	Outcome category	Available extractable data	Feasible synthesis method	Final handling in this review	Reason if not quantitatively synthesized	Related route	Interpretation note
All-cause mortality during follow-up	Additional outcome	Not systematically extractable at arm level from the current route datasets. Some reports mentioned mortality, but usable all-cause death numerators and denominators by prophylaxis regimen were inconsistent or absent.	Narrative summary only, if individual studies reported relevant mortality information.	Not quantitatively synthesized.	Mortality was not consistently reported by prophylaxis arm, follow-up windows differed, and deaths were often not attributable to PCP or prophylaxis regimen. A connected network was not available.	Narrative only / not synthesize d	State transparently that mortality could not be used as a primary comparative endpoint in this evidence base.
PCP-related mortality	Additional outcome	Not reliably extractable across included studies. PCP-attributed deaths were rare, inconsistently defined, and often not separable from all-cause mortality or PCP incidence reporting.	Narrative summary only when explicitly reported.	Not quantitatively synthesized.	Attribution to PCP was inconsistent; events were too sparse for stable comparative or network estimates.	Narrative only / not synthesize d	Absence of a meta-analysis should be described as an evidence/reportin g limitation, not as evidence of no mortality difference.
Total adverse event rate (any adverse event)	Additional safety outcome	Reported heterogeneously in subsets of studies, but definitions, ascertainment, severity thresholds, and denominators were inconsistent. The most consistently extractable safety endpoint was discontinuation due to adverse events.	Not suitable for NMA or pooled pairwise synthesis across all regimens.	Not quantitatively synthesized; adverse-event discontinuation was used as the quantitative safety/tolerability endpoint.	Overall AE counts combined minor and clinically important events inconsistently, and open-label discontinuation/reporting practices differed by study.	Narrative only; A-S/B for discontinu ation	Using discontinuation rather than any-AE rate improves interpretability and avoids mixing non-comparable event definitions.

Protocol-specified outcome	Outcome category	Available extractable data	Feasible synthesis method	Final handling in this review	Reason if not quantitatively synthesized	Related route	Interpretation note
Haematological toxicity (e.g., leukopenia, thrombocytopenia, cytopenia, anemia, methemoglobinemia)	Additional specific safety outcome	For TMP-SMX, cytopenia or marrow suppression was often reported as a reason for intolerance/discontinuation rather than as a consistently adjudicated incident toxicity. Important dapsone-related toxicity was reported descriptively in three Route C studies, including methemoglobinemia, anemia, or author-defined adverse drug events. Because definitions were heterogeneous, these data were not pooled as a single haematological toxicity endpoint.	Descriptive synthesis only. Not appropriate for a single pooled NMA toxicity endpoint.	Reported descriptively in Route C safety tables and, where applicable, as reasons for TMP-SMX discontinuation in Route B; not meta-analysed as a comparative toxicity outcome.	Toxicity definitions differed by drug and study; dapsone methemoglobinemia/anemia is not directly equivalent to TMP-SMX leukopenia/thrombocytopenia; denominators and severity thresholds were inconsistent.	B reasons; C descriptive safety	Present as clinically important safety context, especially for second-line dapsone. Any descriptive dapsone toxicity counts should not be interpreted as a pooled haematological-toxicity incidence estimate or used for comparative ranking.
Hepatotoxicity or transaminitis	Additional specific safety outcome	Sporadically reported, often as a reason for TMP-SMX discontinuation or alternative prophylaxis rather than as arm-level incident hepatotoxicity.	Narrative summary only.	Not quantitatively synthesized.	Definitions and laboratory thresholds were inconsistent or not reported; per-arm denominators were generally unavailable.	Narrative only / B-C context	Mention as part of reporting limitations and reasons for switching, not as a pooled toxicity endpoint.
Nephrotoxicity, acute kidney injury, or renal dysfunction	Additional specific safety outcome	Reported inconsistently as baseline contraindication, renal dysfunction, or reason for TMP-SMX withdrawal in some second-line studies; not consistently captured as incident prophylaxis-related toxicity.	Narrative summary only.	Not quantitatively synthesized.	The outcome was variably defined and confounded by underlying transplant/rheumatic disease status, kidney function, and dose adjustment; arm-level incident data were insufficient.	Narrative only / B-C context	Avoid implying comparable renal-toxicity rates across drugs.

Protocol-specified outcome	Outcome category	Available extractable data	Feasible synthesis method	Final handling in this review	Reason if not quantitatively synthesized	Related route	Interpretation note
Hypersensitivity reactions, rash, or severe cutaneous reactions	Additional specific safety outcome	Reported in some studies as TMP-SMX intolerance or withdrawal reason (including rash or severe hypersensitivity terms), but not consistently adjudicated across regimens.	Narrative summary only.	Not quantitatively synthesized.	Case definitions ranged from rash/intolerance to severe reactions; denominators by regimen and severity were inconsistent.	Narrative only / B-C context	Useful for explaining second-line selection but not for comparative safety inference.
Hyperkalemia	Additional specific safety outcome	Reported in some studies as a reason for TMP-SMX discontinuation or second-line prophylaxis, especially in transplant cohorts; arm-level incident hyperkalemia was not consistently extractable across regimens.	Narrative summary only.	Not quantitatively synthesized.	Laboratory thresholds, attribution, follow-up windows, and denominators were inconsistently reported; no connected comparative network was available.	Narrative only / B-C context	State explicitly that hyperkalemia could not be pooled despite being clinically relevant.
Breakthrough PCP during second-line prophylaxis after TMP-SMX intolerance, contraindication, toxicity, or discontinuation	Additional / route-specific effectiveness outcome	Extractable in the primary Route C analysis: 12 reports, 16 arms, 5/1,056 breakthrough PCP events/participants. Drug-specific descriptive data were atovaquone 5/495, dapsone 0/335, and pentamidine 0/226.	Single-arm descriptive proportions feasible. Comparative NMA and treatment ranking not feasible.	Synthesized descriptively in Route C. No drug-to-drug comparisons, NMA, or ranking were performed.	Second-line populations were selected by intolerance/contraindication, events were sparse, and some denominators mixed initial alternative prophylaxis with switch-after-intolerance patients or required inference.	C	Interpret as low observed breakthrough frequency in selected second-line cohorts, not as comparative evidence that one second-line drug is superior.

Protocol-specified outcome	Outcome category	Available extractable data	Feasible synthesis method	Final handling in this review	Reason if not quantitatively synthesized	Related route	Interpretation note
Second-line prophylaxis tolerability or toxicity after TMP-SMX intolerance/discontinuation	Additional safety / descriptive route-specific outcome	Route C had descriptive safety data where extractable: atovaquone AE/tolerability discontinuation 4/167 across 4 studies; dapsone AE/tolerability discontinuation 80/223 across 2 studies; pentamidine AE/tolerability discontinuation 11/124 across 3 studies. Important dapsone-related toxicity or adverse drug events were reported in 92/249 patients across three studies, but these events were heterogeneously defined and should not be interpreted as a pooled incidence estimate.	Descriptive summary only.	Reported in Route C safety/toxicity descriptive tables; not pooled as a comparative safety endpoint.	Definitions, ascertainment, and denominators varied substantially; not all studies reported second-line safety; patients receiving second-line agents were selected by TMP-SMX intolerance and are not exchangeable with first-line prophylaxis cohorts.	C descriptive safety	Use to contextualize second-line choices and limitations, not to rank atovaquone, dapsone, and pentamidine. These crude descriptive proportions were not statistically pooled and should not be compared across agents because indications, definitions, and denominators differed.

Supplementary Table S9. Protocol-specified outcomes and final analytical handling.

This table distinguishes protocol-consistent analytical adaptation from true non-reporting. Outcomes were not excluded because they were unfavorable; they were not quantitatively synthesized when the published evidence lacked consistent arm-level data, comparable definitions, or a connected and clinically exchangeable network.

Protocol-specified item or analytical objective	Planned role in protocol/design	Final analytical handling	Protocol-consistency judgement	Rationale and reporting location
Effect measure for binary outcomes	Risk ratios were specified in the protocol for binary outcomes.	Odds ratios were used in the final sparse-event network meta-analysis and active-safety synthesis, with direction of effect interpreted consistently.	Analytical adaptation requiring transparent reporting.	The final evidence base contained rare events, sparse active nodes, and several zero-event arms. ORs were used for model stability and compatibility with the implemented NMA framework. This adaptation should be reported in Methods and Limitations. If feasible, an RR-based sensitivity analysis can be considered as a robustness check.
Primary effectiveness outcome: confirmed/probable PCP incidence	Planned as the principal comparative efficacy outcome for available prophylaxis regimens.	Handled in Route A-E as the primary comparative effectiveness network meta-analysis.	Protocol-consistent.	Per-arm event and denominator data were available for a connected comparative network. Report in main Methods/Results and the primary-effectiveness supplementary tables.
Primary safety/tolerability outcome: discontinuation due to adverse events	Planned as the principal safety outcome for comparing regimen tolerability.	Handled in Route A-S for active TMP-SMX dose comparison and Route B for TMP-SMX discontinuation burden/subgroups.	Protocol-consistent analytical adaptation.	Only low/reduced-dose versus standard/conventional-dose TMP-SMX formed an active-comparator dataset with extractable per-arm discontinuation data. Single-arm TMP-SMX discontinuation data were summarized separately. Report in the main manuscript and the active-safety and TMP-SMX discontinuation supplementary tables.
Comparative ranking of all prophylaxis regimens	Network meta-analysis was intended when data allowed connected comparisons among TMP-SMX standard, TMP-SMX low, atovaquone, dapsone, pentamidine, and no prophylaxis.	Performed only for PCP incidence in the A-E evidence base. No safety ranking and no second-line ranking were performed. Atovaquone was limited to sensitivity analysis, and dapsone was not estimable in the connected primary effectiveness network.	Protocol-consistent limitation due to data structure.	Atovaquone/dapsone/pentamidine data were mostly second-line, non-comparative, or sparse; including them as exchangeable first-line active nodes would violate transitivity or produce disconnected/unstable estimates.
All-cause mortality and PCP-related mortality	Planned additional outcomes.	Not meta-analysed; narrative mention only where relevant.	Protocol-consistent non-synthesis due to insufficient extractable data.	Mortality reporting was inconsistent, frequently not arm-specific, and often not attributable to PCP or prophylaxis.
Total adverse events	Planned additional safety outcome.	Not pooled; adverse-event discontinuation was used as the quantitative safety/tolerability endpoint.	Protocol-consistent analytical prioritization.	Any-AE definitions varied widely, whereas discontinuation due to AE was more consistently extractable and clinically interpretable.
Specific toxicities: haematological toxicity, hepatotoxicity, nephrotoxicity, hypersensitivity, and hyperkalemia	Planned additional safety outcomes.	Not meta-analysed. Report as reasons for discontinuation/switching and as descriptive Route C safety findings where extractable.	Protocol-consistent non-synthesis plus limitation.	Specific toxicity definitions, thresholds, severity, attribution, and denominators were too inconsistent for comparative synthesis.

Protocol-specified item or analytical objective	Planned role in protocol/design	Final analytical handling	Protocol-consistency judgement	Rationale and reporting location
Breakthrough PCP after TMP-SMX intolerance/discontinuation and second-line prophylaxis	Consistent with protocol interest in atovaquone, dapsone, and pentamidine and clinically necessary because much alternative-agent evidence was second-line.	Handled as Route C descriptive single-arm evidence. No NMA, no drug-to-drug comparison, and no ranking.	Protocol-consistent analytical adaptation.	Second-line cohorts are selected by TMP-SMX intolerance or contraindication; sparse events and mixed denominators preclude comparative inference. Report in the second-line prophylaxis supplementary tables.
Population/subgroup exploration	Protocol mentioned exploration by clinical subgroups, including rheumatic disease and solid-organ transplant populations.	Used in descriptive characterization and Route B subgroup analyses; not used for separate A-E subgroup NMA because networks would be too sparse.	Protocol-consistent analytical adaptation.	Preserves subgroup information where data are adequate while avoiding unstable disconnected subgroup networks.
Unreported or unreliably reported outcomes	Protocol included broad safety and mortality outcomes subject to data availability.	Classified as narrative only or not synthesized in Supplementary Table S8.	Transparent reporting rather than protocol deviation.	The feasibility table provides transparent justification for outcomes that were not quantitatively synthesized.

Supplementary Table S10. Mapping of protocol-specified outcomes to final analysis routes.

The table provides a concise route-level map for the final synthesis framework.

Protocol-specified outcome group	Final route or handling	One-line rationale
PCP incidence	A-E NMA	Comparative arm-level data and connected network were available.
Adverse-event discontinuation	A-S pairwise active-comparator synthesis; B single-arm burden analysis	Comparative safety data were available only for TMP-SMX dose strategies; broader TMP-SMX discontinuation data were single-arm.
Breakthrough PCP after second-line prophylaxis	C descriptive analysis	Second-line data were selected by TMP-SMX intolerance and not exchangeable for comparative ranking.
Mortality and total adverse events	Narrative only / not synthesized	Arm-level, comparable, regimen-specific data were insufficient.
Specific toxicities	Narrative/contextual reporting; C descriptive safety where available	Definitions, attribution, and denominators were inconsistent across drugs and studies.

Abbreviations: A-E, primary comparative effectiveness analysis; A-S, active-regimen safety analysis; AE, adverse event; ADE, adverse drug event; B, TMP-SMX discontinuation subgroup/single-arm discontinuation analysis; C, second-line prophylaxis after TMP-SMX intolerance or discontinuation; CI, confidence interval; NMA, network meta-analysis; OR, odds ratio; RR, risk ratio; TMP-SMX, trimethoprim-sulfamethoxazole.

Section S5. Route A-E: primary comparative effectiveness analysis

Node	Clinical definition	Dose/regimen mapping	Base-case input summary	Base-case handling	Sensitivity or narrative role	Key caveats / reporting rule
No prophylaxis / observation / placebo	Placebo, observation, or no pharmacological PCP prophylaxis.	No active prophylactic drug; used as the reference comparator.	21 studies; 21 arms; 214/27066 PCP events/participants	Included as reference node.	Reference node in all base-case and most sensitivity analyses.	Baseline PCP risk varied across rheumatic/autoimmune and solid-organ transplant settings; use as comparator, not as a recommended strategy.
TMP-SMX standard/conventional dose	Conventional TMP-SMX prophylaxis in eligible non-HIV, non-malignant immunocompromised adults.	SS daily, DS daily, DS three-times-weekly, or renal-adjusted equivalent; dose-unclear arms retained with flags unless excluded in strict-dose sensitivity.	21 studies; 21 arms; 18/5107 PCP events/participants	Included.	Retained in strict-dose, pentamidine-exclusion, and atovaquone sensitivity analyses.	Primary active comparator; emphasize protective association versus no prophylaxis while acknowledging non-randomized evidence and some dose uncertainty.
TMP-SMX low/reduced dose	Reduced-intensity TMP-SMX prophylaxis.	Clearly low/reduced dose, half-strength, dose-escalation, or exposure below standard/conventional prophylaxis.	12 studies; 12 arms; 3/2145 PCP events/participants	Included.	Retained in strict-dose, pentamidine-exclusion, and atovaquone sensitivity analyses.	Protective direction versus no prophylaxis; low-dose superiority over standard dose is not established because direct evidence was neutral and node-splitting was borderline inconsistent.
Pentamidine	Inhaled or intravenous pentamidine prophylaxis with extractable PCP incidence.	Pentamidine prophylaxis arm; route and clinical context varied.	3 studies; 3 arms; 8/155 PCP events/participants	Included with explicit caution.	Pentamidine-exclusion sensitivity performed.	Sparse active node; high likelihood of channeling/indication bias. Do not interpret unfavorable estimate as causal harm or inferiority.
Atovaquone	Prespecified alternative prophylaxis regimen.	Atovaquone prophylaxis arm; available comparative data largely reflected use after TMP-SMX intolerance/discontinuation.	Sensitivity dataset: 1 study; 1 atovaquone arm; 5/107 PCP events/participants.	Excluded from base-case.	Sensitivity/narrative node only.	Not exchangeable with first-line TMP-SMX/no-prophylaxis cohorts; do not rank as a base-case active regimen.
Dapsone	Prespecified alternative prophylaxis regimen.	Dapsone prophylaxis; available data were mainly descriptive/second-line or disconnected from the A-E comparative effectiveness network.	Not estimable in the connected base-case A-E network.	Not included in base-case estimates.	Discuss narratively only if needed.	Prespecified but non-estimable; absence of estimable evidence is not evidence of no effect.

Note. Base-case totals: 27 studies, 57 arms, and 243 PCP events among 34,473 participants. Node totals are arm-level summaries and studies can contribute to more than one node.

Supplementary Table S12. Base-case network meta-analysis estimates and league table for PCP incidence.

Comparison	Network OR	95% CI	Primary interpretation
TMP-SMX standard dose vs no prophylaxis / observation / placebo	0.30	0.18–0.48	Protective association; direct, network, and RR sensitivity estimates were directionally consistent.
TMP-SMX low dose vs no prophylaxis / observation / placebo	0.08	0.03–0.18	Protective association; magnitude should be interpreted cautiously because events were sparse and transitivity concerns remain.
TMP-SMX low dose vs TMP-SMX standard dose	0.26	0.10–0.66	Network estimate favored low dose, but direct evidence was neutral and node-splitting was borderline inconsistent; do not claim superiority.
Pentamidine vs no prophylaxis / observation / placebo	6.37	2.60–15.64	Unfavorable estimate, but evidence was sparse and likely channeling-biased; not causal harm or inferiority.

League table component. Cell values are odds ratios for the row regimen versus the column regimen.

Row regimen vs column regimen	No prophylaxis	TMP-SMX standard	TMP-SMX low	Pentamidine
No prophylaxis	No prophylaxis	3.36 (2.07-5.46)	12.97 (5.44-30.96)	0.16 (0.06-0.39)
TMP-SMX standard	0.30 (0.18-0.48)	TMP-SMX standard	3.86 (1.52-9.76)	0.05 (0.02-0.13)
TMP-SMX low	0.08 (0.03-0.18)	0.26 (0.10-0.66)	TMP-SMX low	0.01 (0.00-0.05)
Pentamidine	6.37 (2.60-15.64)	21.43 (7.93-57.91)	73.92 (20.81-262.53)	Pentamidine

Note. OR <1 favors the row regimen for PCP prevention. Comparisons involving pentamidine were based on sparse and potentially channeling-biased evidence and should not be interpreted as causal evidence of harm, inferiority, or active-regimen ranking. The pentamidine versus TMP-SMX low-dose contrast was reconstructed from the same arm-level data and should be interpreted qualitatively.

Supplementary Table S13. Direct pairwise evidence supporting the A-E base-case network.

Direct comparison	Studies	Events/participants in regimen 1	Events/participants in regimen 0	Direct OR (95% CI)	Network OR (95% CI)	Direct RR sensitivity (95% CI)	Interpretation
TMP-SMX standard vs No prophylaxis	15	17/4741	160/26415	0.32 (0.19-0.54)	0.30 (0.18-0.48)	0.35 (0.21-0.56)	Primary protective contrast; direct and network evidence are directionally consistent.

Direct comparison	Studies	Events/participants in regimen 1	Events/participants in regimen 0	Direct OR (95% CI)	Network OR (95% CI)	Direct RR sensitivity (95% CI)	Interpretation
TMP-SMX low vs No prophylaxis	7	3/1689	56/698	0.05 (0.02-0.17)	0.08 (0.03-0.18)	0.06 (0.02-0.18)	Protective direction vs no prophylaxis; sparse events and transitivity concerns remain.
TMP-SMX low vs TMP-SMX standard	6	0/467	1/365	0.93 (0.20-4.39)	0.26 (0.10-0.66)	0.93 (0.20-4.27)	Direct pairwise evidence was neutral and did not demonstrate superiority of low-dose over standard-dose TMP-SMX; the network estimate favoring low-dose TMP-SMX should therefore be interpreted cautiously.
Pentamidine vs No prophylaxis	2	5/137	66/22560	6.01 (2.32-15.56)	6.37 (2.60-15.64)	5.79 (2.31-14.47)	Sparse and likely affected by channeling/indication bias; avoid causal interpretation.
Pentamidine vs TMP-SMX standard	3	8/155	0/2650	44.47 (7.00-282.68)	21.43 (7.93-57.91)	38.98 (6.35-239.13)	Sparse and likely affected by channeling/indication bias; avoid causal interpretation.

Note. Direct estimates were calculated from the arm-level base-case dataset with a 0.5 continuity correction when needed and DerSimonian-Laird random-effects pooling. Direct estimates show where the network is supported by head-to-head evidence and where estimates are mostly indirect or sparse.

Supplementary Table S14. Heterogeneity diagnostics for the primary effectiveness NMA and key direct comparisons.

Analysis	Studies	Model	tau²	I²	Q	Interpretation
A-E base-case network NMA	27	Random-effects, REML (R netmeta)	0.013	Not directly reported in the primary R netmeta output	See node-splitting/global decomposition; sparse network	Low estimated between-study heterogeneity, but rare events and transitivity concerns dominate interpretation.
Direct pairwise: TMP-SMX standard vs no prophylaxis	15	Direct DL random effects	0.050	4.3%	14.63	Direct evidence supports a protective association; heterogeneity should be interpreted cautiously with sparse events.
Direct pairwise: TMP-SMX low vs no prophylaxis	7	Direct DL random effects	0.830	31.3%	8.73	Protective direct evidence; some studies have zero events and small arms.
Direct pairwise: TMP-SMX low vs standard	6	Direct DL random effects	0.000	0.0%	0.64	Direct evidence is neutral; use to temper the network estimate.
Direct pairwise: pentamidine vs no prophylaxis	2	Direct DL random effects	0.000	0.0%	0.00	Sparse/channeling-biased direct evidence; not causal.

Note. The primary network tau estimate is from the formal R netmeta random-effects REML model. Pairwise tau², I², and Q values were calculated from direct pairwise contrasts to aid interpretation. Low numerical heterogeneity does not remove concerns about confounding, channeling, sparse events, or clinical intransitivity; $I^2=0$ should not be interpreted as absence of clinical heterogeneity.

Supplementary Table S15. Inconsistency and node-splitting diagnostics for the A-E network.

Dataset	Comparison	Direct evidence	Network/indirect evidence	Node-splitting p	Global/design-by-treatment assessment	Interpretation
basecase_4node	TMP-SMX low vs TMP-SMX standard	OR 0.93 (95% CI 0.20-4.39) from the node-splitting output	Network OR 0.259 (95% CI 0.103-0.656)	0.045	Formal design-by-treatment model not stable due to sparse closed loops and rare events	Borderline inconsistency; do not claim low-dose superiority.
strict_dose	TMP-SMX low vs TMP-SMX standard	OR 1.16 (95% CI 0.20-6.80) from the node-splitting output	Network OR 0.262 (95% CI 0.093-0.734)	0.041	Limited; sensitivity retains sparse loop structure	Inconsistency persists after dose-adjudication sensitivity.
exclude_pentamidine	TMP-SMX low vs TMP-SMX standard	Direct evidence neutral	Network OR 0.282 (95% CI 0.107-0.741)	0.061	Limited	Borderline after removing pentamidine; core caution remains.
basecase_4node	TMP-SMX standard vs no prophylaxis	Direct evidence dominant	Network OR 0.297 (95% CI 0.183-0.483)	0.193	No reliable full global assessment; no major local signal	Primary contrast appears stable.

Dataset	Comparison	Direct evidence	Network/indirect evidence	Node-splitting p	Global/design-by-treatment assessment	Interpretation
basecase_4node	pentamidine vs no prophylaxis	Sparse direct evidence	Network OR 6.372 (95% CI 2.596-15.642)	0.986	No statistical local inconsistency, but transitivity is weak	Model estimate should not be read as causal harm because of channeling bias.

Note. Node splitting was performed wherever direct and indirect evidence were estimable. For low-dose versus standard-dose TMP-SMX, the direct-indirect comparison p value was 0.045. The global design-by-treatment interaction was not statistically significant (Q = 7.80, df = 5, p = 0.168). Two single-study designs had non-estimable decomposition p values. These findings, together with sparse events and clinical intransitivity, mean that low-dose superiority should not be claimed.

Supplementary Table S16. Transitivity and node-adjudication table.

Node / comparison	Main contributing populations	Prophylaxis context	Exchangeability concern	Direction of concern	Final interpretation constraint
No prophylaxis baseline risk variation	Rheumatic/autoimmune disease and solid-organ transplant cohorts.	Placebo/observation/no drug; clinical thresholds for prophylaxis varied by setting.	Baseline PCP risk and observation periods were heterogeneous.	May change absolute risk and indirectly influence relative comparisons.	Use no prophylaxis as the network reference, not as a clinically recommended strategy.
Rheumatic/autoimmune vs solid-organ transplant differences	Both populations contributed to the reference and TMP-SMX nodes; some active nodes were more population-specific.	Underlying immunosuppression, prophylaxis indication, and follow-up differed.	Partial exchangeability only; cross-population indirect comparisons require caution.	May influence apparent active-regimen effects and precision.	Frame conclusions around TMP-SMX versus no prophylaxis, not definitive active-drug hierarchy.
TMP-SMX low dose vs TMP-SMX standard dose	Mostly transplant and selected rheumatic/autoimmune cohorts.	Dose selection may reflect renal function, frailty, expected intolerance, or center protocols.	Low-dose arms may have lower baseline risk and multiple zero-event arms.	Indirect evidence may favor low dose more than direct evidence supports.	Do not claim low-dose superiority; direct evidence was neutral.
Pentamidine vs no prophylaxis	Sparse autoimmune/transplant data; alternative-agent contexts.	Often used when TMP-SMX was unsuitable or in selected high-risk patients.	Severe channeling/indication bias; pentamidine recipients may not be comparable with no-prophylaxis arms.	Unfavorable OR may reflect patient selection rather than drug effect.	Do not interpret as causal harm or inferiority.
Atovaquone sensitivity node	Single connected comparative context, largely after TMP-SMX intolerance/discontinuation.	Second-line or intolerance-related prophylaxis context.	Not exchangeable with first-line TMP-SMX/no-prophylaxis cohorts.	May bias atovaquone against TMP-SMX in active comparisons.	Sensitivity/narrative only; no ranking.

Node / comparison	Main contributing populations	Prophylaxis context	Exchangeability concern	Direction of concern	Final interpretation constraint
Dapsone non-estimable node	No sufficient connected A-E comparative arm-level PCP incidence evidence.	Mostly descriptive or second-line contexts.	Disconnected from the estimable base-case network.	No stable network estimate can be generated.	Prespecified but non-estimable; not evidence of no effect.

Note. This table states the clinical exchangeability concerns that limit active-regimen ranking. These concerns were used to constrain interpretation rather than to change the pre-specified prophylaxis nodes.

Supplementary Table S17. TMP-SMX study-arm dose classification across A-E, A-S, and Route B.

Study	Arm	Source dose/frequency	Author term	Final class	Basis	Dose uncertain?	Analysis	Sensitivity handling
Aggoun 2025[50]	low-dose SXT	20/100 mg	low-dose SXT	Explicit low/reduced dose	Explicit low-dose TMP-SMX prophylaxis.	No	A-E base-case; A-S base-case; Route B main	Retained in A-E strict-dose sensitivity
Aggoun 2025	standard-dose SXT	80/400 mg	standard-dose SXT	Standard/conventional dose	Explicit standard/conventional dose.	No	A-E base-case; A-S base-case; Route B main	Retained in A-E strict-dose sensitivity
Chan 2021[35]	co-trimoxazole	co-trimoxazole ≥2 weeks	co-trimoxazole	Unclear/standard	Dose not extractable; not described as low-dose; classify as standard/unspecified with strict-dose sensitivity exclusion.	Yes	A-E base-case	Excluded from A-E strict-dose sensitivity
Chapman 2019[30]	unclear/standard	TMP-SMX 3 vs 6 month protocol	unclear/standard	Unclear/standard	Dose/frequency was not extractable; retained only with a dose-unclear flag and never classified as low dose.	Yes	Route B main	Retained in the final Route B dose-subgroup analysis
Chen 2022[41]	daily TMP-SMX	20/100 mg daily	daily TMP-SMX	Explicit low/reduced dose	20/100 mg daily/every-other-day is below SS daily; explicit low-dose regimen.	No	A-E base-case	Retained in A-E strict-dose sensitivity
Chen 2022	every-other-day TMP-SMX	20/100 mg every other day	every-other-day TMP-SMX	Explicit low/reduced dose	20/100 mg daily/every-other-day is below SS daily; explicit low-dose regimen.	No	A-E base-case	Retained in A-E strict-dose sensitivity
Chen Tongqing 2011[15]	TMP-SMX 1 year	0.48 g/day for 1 year	TMP-SMX 1 year	Explicit low/reduced dose	Explicit low-dose TMP-SMX prophylaxis.	No	A-E base-case	Excluded from A-E strict-dose sensitivity
Chen Tongqing 2011	TMP-SMX 6 months	0.48 g/day for 6 months	TMP-SMX 6 months	Explicit low/reduced dose	Explicit low-dose TMP-SMX prophylaxis.	No	A-E base-case	Excluded from A-E strict-dose sensitivity
Elinder 1992[2]	cotrimoxazole	480 mg daily/every other day renal-adjusted	cotrimoxazole	Explicit low/reduced dose	Explicit low-dose TMP-SMX prophylaxis.	No	A-E base-case	Retained in A-E strict-dose sensitivity

Study	Arm	Source dose/frequency	Author term	Final class	Basis	Dose uncertain?	Analysis	Sensitivity handling
Enomoto 2008[11]	TMP-SMX	TMP-SMX prophylaxis	TMP-SMX	Unclear/standard	TMP-SMX prophylaxis not explicitly described as low-dose; pragmatically mapped to standard dose with uncertainty flag.	Yes	A-E base-case	Excluded from A-E strict-dose sensitivity
Faraji 2022[42]	cotrimoxazole	two adult tablets 80/400 mg each, 3 days/week	cotrimoxazole	Standard/conventional dose	Intermittent standard-strength cotrimoxazole regimen = standard/conventional dose.	No	A-E base-case; Route B main	Retained in A-E strict-dose sensitivity
Havele 2021[36]	standard/conventional	TMP-SMX DS 800/160 mg three times weekly with methotrexate	standard/conventional	Standard/conventional dose	Dose/frequency or an explicit author dose label was extractable from the source report.	No	Route B main	Retained in the final Route B dose-subgroup analysis
Higgins 1989[1]	low-dose cotrimoxazole	480 mg once daily	low-dose cotrimoxazole	Explicit low/reduced dose	Explicit low-dose TMP-SMX prophylaxis.	No	A-E base-case; Route B main	Retained in A-E strict-dose sensitivity
Hiramoto 2024[47]	unclear/mixed	SMX/TMP prophylaxis; mostly one tablet or half tablet per day	unclear/mixed	Unclear/mixed	Dose/frequency was not extractable; retained only with a dose-unclear flag and never classified as low dose.	Yes	Route B main	Retained in the final Route B dose-subgroup analysis
Honda 2021[37]	TMP-SMX	TMP-SMX prophylaxis	TMP-SMX	Unclear/standard	Dose not extractable; not described as low-dose; classify as standard/unspecified with strict-dose sensitivity exclusion.	Yes	A-E base-case; Route B main	Excluded from A-E strict-dose sensitivity
Hudson/Tinkham 2025[52]	unclear/standard	TMP/SMX prophylaxis after abdominal SOT	unclear/standard	Unclear/standard	Dose/frequency was not extractable; retained only with a dose-unclear flag and never classified as low dose.	Yes	Route B main	Retained in the final Route B dose-subgroup analysis
Izuka 2021[38]	unclear/standard	prophylactic TMP-SMX	unclear/standard	Unclear/standard	Dose/frequency was not extractable; retained only with a dose-unclear flag and never classified as low dose.	Yes	Route B main	Retained in the final Route B dose-subgroup analysis
Jinno 2022[43]	unclear/standard	TMP-SMX prophylaxis	unclear/standard	Unclear/standard	Dose/frequency was not extractable; retained only with a dose-unclear flag and never classified as low dose.	Yes	Route B main; A-E atovaquone sensitivity	Retained in the final Route B dose-subgroup analysis
Katsuyama 2014[20]	unclear/standard	TMP-SMX prophylaxis	unclear/standard	Unclear/standard	Dose/frequency was not extractable; retained only with a dose-unclear flag and never classified as low dose.	Yes	Route B main	Retained in the final Route B dose-subgroup analysis

Study	Arm	Source dose/frequency	Author term	Final class	Basis	Dose uncertain?	Analysis	Sensitivity handling
Kimura 2008[12]	TMP-SMX	TMP-SMX	TMP-SMX	Unclear/standard	TMP-SMX dose not extractable; not described as low-dose; classify as standard/unspecified with sensitivity exclusion.	Yes	A-E base-case	Excluded from A-E strict-dose sensitivity
Kitazawa 2019[31]	unclear/standard	initial TMP-SMX then switch if AE	unclear/standard	Unclear/standard	Dose/frequency was not extractable; retained only with a dose-unclear flag and never classified as low dose.	Yes	Route B main	Retained in the final Route B dose-subgroup analysis
Liu Wenjie 2012	TMP-SMX	0.48 g/day	TMP-SMX	Explicit low/reduced dose	Explicit low-dose TMP-SMX prophylaxis.	No	A-E base-case	Retained in A-E strict-dose sensitivity
Maezawa 2012[18]	unclear/standard	prophylactic TMP-SMX	unclear/standard	Unclear/standard	Dose/frequency was not extractable; retained only with a dose-unclear flag and never classified as low dose.	Yes	Route B main	Retained in the final Route B dose-subgroup analysis
McLaughlin 2017[26]	unclear/standard	initial TMP-SMX prophylaxis	unclear/standard	Unclear/standard	Dose/frequency was not extractable; retained only with a dose-unclear flag and never classified as low dose.	Yes	Route B main	Retained in the final Route B dose-subgroup analysis
Mitsides 2014[22]	unclear/standard	TMP-SMX prophylaxis	unclear/standard	Unclear/standard	Dose/frequency was not extractable; retained only with a dose-unclear flag and never classified as low dose.	Yes	Route B main	Retained in the final Route B dose-subgroup analysis
Munroe 2025[53]	low/reduced	SMX/TMP single-strength twice weekly	low/reduced	Low/reduced-intensity strategy	Reduced-frequency regimen (single-strength twice weekly); classified as lower intensity based on schedule, not on an explicit low-dose author label.	No	Route B main	Retained in the final Route B dose-subgroup analysis
Munthananuchat 2024[48]	TMP-SMX	eGFR-adjusted 80/400 mg regimen (two tablets daily, one daily, or one three times/week)	TMP-SMX	Standard/renal-adjusted	PDF verified renal-adjusted conventional regimen = standard/conventional dose.	No	A-E base-case; Route B main	Retained in A-E strict-dose sensitivity
Ogawa 2005[9]	TMP-SMX	TMP-SMX prophylaxis	TMP-SMX	Unclear/standard	TMP-SMX prophylaxis not explicitly described as low-dose; pragmatically mapped to standard dose with uncertainty flag.	Yes	A-E base-case	Excluded from A-E strict-dose sensitivity

Study	Arm	Source dose/frequency	Author term	Final class	Basis	Dose uncertain?	Analysis	Sensitivity handling
Ohmura 2024[49]	conventional-dose SMX/TMP	one tablet per day	conventional-dose SMX/TMP	Standard/conventional dose	Explicit standard/conventional dose.	No	A-E base-case; A-S base-case; Route B main	Retained in A-E strict-dose sensitivity
Ohmura 2024	low-dose SMX/TMP	one tablet twice weekly	low-dose SMX/TMP	Explicit low/reduced dose	Explicit low-dose TMP-SMX prophylaxis.	No	A-E base-case; A-S base-case; Route B main	Retained in A-E strict-dose sensitivity
Okada 1999[6]	ST 1 tablet/day	1 tablet/day	ST 1 tablet/day	Low/reduced-intensity strategy	Within-study lower-dose arm (one tablet/day versus two tablets/day); not explicitly labelled low dose by the authors.	No	A-E base-case; A-S base-case; Route B main	Retained in A-E strict-dose sensitivity
Okada 1999	ST 2 tablets/day	2 tablets/day	ST 2 tablets/day	Standard/conventional dose	Explicit standard/conventional dose.	No	A-E base-case; A-S base-case; Route B main	Retained in A-E strict-dose sensitivity
Olsen 1993[3]	daily TMP-SMX	TMP 160/SMX 800 twice daily daily	daily TMP-SMX	Standard/conventional dose	High/intensive TMP-SMX regimen; mapped to standard dose with sensitivity flag.	No	A-E base-case; Route B main	Retained in A-E strict-dose sensitivity
Olsen 1993	intermittent TMP-SMX	TMP 160/SMX 800 twice daily on Mon/Tue/Wed	intermittent TMP-SMX	Standard/conventional dose	High/intermittent TMP-SMX regimen; mapped to standard dose with sensitivity flag.	No	A-E base-case; Route B main	Retained in A-E strict-dose sensitivity
Park 2018[28]	TMP-SMX	SS daily or DS three times weekly	TMP-SMX	Standard/renal-adjusted	Standard prophylaxis with renal adjustment; mapped to standard dose.	No	A-E base-case	Retained in A-E strict-dose sensitivity
Park 2019[32]	TMP-SMX	SS daily; renal adjustment	TMP-SMX	Standard/renal-adjusted	Standard prophylaxis with renal adjustment; mapped to standard dose.	No	A-E base-case; Route B main	Retained in A-E strict-dose sensitivity
Park 2022[44]	TMP-SMX	SS daily or DS three times weekly; renal-adjusted	TMP-SMX	Standard/renal-adjusted	PDF verified: SS daily or DS three-times-weekly; renal-adjusted equivalent = standard/conventional dose.	No	A-E base-case	Retained in A-E strict-dose sensitivity
Park 2023[46]	TMP-SMX	SS daily or DS three times weekly; renal-adjusted	TMP-SMX	Standard/renal-adjusted	PDF verified: SS daily or DS three-times-weekly; renal-adjusted equivalent = standard/conventional dose.	No	A-E base-case	Retained in A-E strict-dose sensitivity
Peterson 2021[39]	unclear/standard	lifelong universal TMP-SMX prophylaxis	unclear/standard	Unclear/standard	Dose/frequency was not extractable; retained only with a dose-unclear flag and never classified as low dose.	Yes	Route B main	Retained in the final Route B dose-subgroup analysis

Study	Arm	Source dose/frequency	Author term	Final class	Basis	Dose uncertain?	Analysis	Sensitivity handling
Prasad 2019[33]	standard/conventional	daily single-strength TMP-SMX with dose reductions	standard/conventional	Standard/conventional dose	Dose/frequency or an explicit author dose label was extractable from the source report.	No	Route B main	Retained in the final Route B dose-subgroup analysis
Soejima 2007[10]	unclear/standard	prophylactic co-trimoxazole	unclear/standard	Unclear/standard	Dose/frequency was not extractable; retained only with a dose-unclear flag and never classified as low dose.	Yes	Route B main	Retained in the final Route B dose-subgroup analysis
Sonomoto 2022[45]	SMX/TMP	SMX/TMP	SMX/TMP	Unclear/standard	Dose not extractable; not described as low-dose; classify as standard/unspecified with strict-dose sensitivity exclusion.	Yes	A-E base-case	Excluded from A-E strict-dose sensitivity
Takenaka 2013[19]	dose-escalation	10% SS escalating to SS daily	dose-escalation	Low/reduced-intensity strategy	Dose-escalation strategy starting at 10% of a single-strength tablet and increasing toward one tablet daily; not a fixed low-dose regimen.	No	A-E base-case; A-S base-case; Route B main	Retained in A-E strict-dose sensitivity
Takenaka 2013	routine	1 SS tablet daily	routine	Standard/conventional dose	Explicit standard/conventional dose.	No	A-E base-case; A-S base-case; Route B main	Retained in A-E strict-dose sensitivity
Torre-Cisneros 1996[5]	low-dose cotrimoxazole	480 mg once daily	low-dose cotrimoxazole	Explicit low/reduced dose	Explicit low-dose TMP-SMX prophylaxis.	No	A-E base-case; Route B main	Retained in A-E strict-dose sensitivity
Torre-Cisneros 1999[7]	low/reduced	daily low-dose TMP-SMX	low/reduced	Explicit low/reduced dose	Dose/frequency or an explicit author dose label was extractable from the source report.	No	Route B main	Retained in the final Route B dose-subgroup analysis
Urbancic 2018[29]	unclear/standard	TMP-SMX prophylaxis with ADR/rechallenge protocol	unclear/standard	Unclear/standard	Dose/frequency was not extractable; retained only with a dose-unclear flag and never classified as low dose.	Yes	Route B main	Retained in the final Route B dose-subgroup analysis
Utsunomiya 2017[27]	ES	40/8 mg escalating to 200/40 mg daily	ES	Explicit low/reduced dose	Dose-escalation low-dose strategy; mapped to low dose with sensitivity flag.	No	A-E base-case; A-S base-case; Route B main	Retained in A-E strict-dose sensitivity
Utsunomiya 2017	HS	200/40 mg daily	HS	Explicit low/reduced dose	Explicit low-dose TMP-SMX prophylaxis.	No	A-E base-case; A-S base-case; Route B main	Retained in A-E strict-dose sensitivity

Study	Arm	Source dose/frequency	Author term	Final class	Basis	Dose uncertain?	Analysis	Sensitivity handling
Utsunomiya 2017	SS	400/80 mg daily	SS	Standard/conventional dose	Explicit standard/conventional dose.	No	A-E base-case; A-S base-case; Route B main	Retained in A-E strict-dose sensitivity
Vananuvat 2011[14]	SMZ/TMP	SMZ/TMP single-strength once daily	SMZ/TMP	Standard/conventional dose	SS daily is classified as standard/conventional in base-case; sensitivity can reclassify/exclude historical low-dose framing.	No	A-E base-case	Retained in A-E strict-dose sensitivity
Yamamoto 2014[23]	daily TMP-SMX	80/400 mg daily	daily TMP-SMX	Standard/conventional dose	Explicit standard/conventional dose.	No	A-E base-case; A-S base-case; Route B main	Retained in A-E strict-dose sensitivity
Yamamoto 2014	twice weekly TMP-SMX	80/400 mg twice weekly	twice weekly TMP-SMX	Explicit low/reduced dose	Intermittent low-dose strategy; mapped to low dose with sensitivity flag.	No	A-E base-case; A-S base-case; Route B main	Retained in A-E strict-dose sensitivity
Yamanaga 2020[34]	low/reduced	very-low-dose TMP-SMX re-administration	low/reduced	Explicit low/reduced dose	Dose/frequency or an explicit author dose label was extractable from the source report.	No	Route B main	Retained in the final Route B dose-subgroup analysis
Yang 2021[40]	standard/conventional	TWDS or DSS TMP-SMX	standard/conventional	Standard/conventional dose	Dose/frequency or an explicit author dose label was extractable from the source report.	No	Route B main; A-E schedule sensitivity	Retained in the final Route B dose-subgroup analysis
Zhu 2010[13]	SMZ-TMP prophylaxis	SMZ-TMP 2 tablets daily for 1 month	SMZ-TMP prophylaxis	Standard/conventional dose	Standard-dose short-course regimen; mapped to standard dose with sensitivity flag.	No	A-E base-case; Route B main	Excluded from A-E strict-dose sensitivity

Summary: This table includes 46 unique studies and 56 unique TMP-SMX arms. Explicit dosing information was available for 37 arms from 27 studies, whereas 19 arms from 19 studies had unclear dosing. Six dose-unclear arms entered the A-E standard-dose node with a dose-unclear flag. The strict-dose A-E sensitivity analysis excluded eight studies comprising nine arms. No unspecified-dose arm was classified as low dose.

Supplementary Table S18. Sensitivity analyses for the A-E primary comparative effectiveness network meta-analysis.

Sensitivity analysis	Evidence base / nodes	Comparison	Network OR (95% CI)	tau	Conclusion
Base-case random-effects REML	27 studies; 4 nodes	TMP-SMX standard dose vs no prophylaxis	0.30 (0.18–0.48)	0.1153	Primary protective contrast; direction stable.

Sensitivity analysis	Evidence base / nodes	Comparison	Network OR (95% CI)	tau	Conclusion
Base-case random-effects REML	27 studies; 4 nodes	TMP-SMX low dose vs no prophylaxis	0.08 (0.03–0.18)	0.1153	Protective direction; magnitude limited by sparse events/transitivity.
Base-case random-effects REML	27 studies; 4 nodes	TMP-SMX low dose vs TMP-SMX standard dose	0.26 (0.10–0.66)	0.1153	Network favors low dose but direct evidence was neutral; interpret cautiously.
Base-case random-effects REML	27 studies; 4 nodes	Pentamidine vs no prophylaxis	6.37 (2.60–15.64)	0.1153	Sparse/channeling-biased node; not causal harm.
Strict-dose sensitivity	19 studies; 4 nodes; dose-unclear/borderline studies excluded	TMP-SMX standard dose vs no prophylaxis	0.37 (0.21–0.66)	0.0001	Protective direction retained.
Strict-dose sensitivity	19 studies; 4 nodes; dose-unclear/borderline studies excluded	TMP-SMX low dose vs no prophylaxis	0.10 (0.04–0.25)	0.0001	Protective direction retained.
Strict-dose sensitivity	19 studies; 4 nodes; dose-unclear/borderline studies excluded	TMP-SMX low dose vs TMP-SMX standard dose	0.26 (0.09–0.73)	0.0001	Indirect pattern retained, but direct evidence remains neutral and node-splitting remains borderline.
Pentamidine-exclusion sensitivity	27 studies; 3 nodes; pentamidine removed	TMP-SMX standard dose vs no prophylaxis	0.27 (0.15–0.46)	0.3583	Protective direction retained.
Pentamidine-exclusion sensitivity	27 studies; 3 nodes; pentamidine removed	TMP-SMX low dose vs no prophylaxis	0.08 (0.03–0.18)	0.3583	Protective direction retained.
Pentamidine-exclusion sensitivity	27 studies; 3 nodes; pentamidine removed	TMP-SMX low dose vs TMP-SMX standard dose	0.28 (0.11–0.74)	0.3583	Core caution remains; direct evidence did not show superiority.
Atovaquone sensitivity network	28 studies; 5 nodes; atovaquone added through Jinno 2022[43] context	TMP-SMX standard dose vs no prophylaxis	0.30 (0.18–0.48)	0.1153	Core TMP-SMX estimate unchanged.
Atovaquone sensitivity network	28 studies; 5 nodes; atovaquone added through Jinno 2022 context	TMP-SMX low dose vs no prophylaxis	0.08 (0.03–0.18)	0.1153	Core TMP-SMX estimate unchanged.
Atovaquone sensitivity network	28 studies; 5 nodes; atovaquone added through Jinno 2022 context	Atovaquone vs TMP-SMX standard dose	6.84 (1.29–36.35)	0.1153	Sensitivity/narrative only; likely indication/channeling bias.

Sensitivity analysis	Evidence base / nodes	Comparison	Network OR (95% CI)	tau	Conclusion
Fixed-effect vs random-effects comparison	Base-case 4-node network	Key estimates	FE and RE estimates were materially similar for the primary TMP-SMX contrasts.	FE tau=0; RE tau=0.1153	Supports numerical robustness, but does not remove transitivity or RoB limitations.

Note. Sensitivity analyses were used to test robustness of the established analytical choices, not to introduce active-regimen ranking. Atovaquone remained a sensitivity/narrative node because its comparative data largely reflected second-line use after TMP-SMX intolerance or discontinuation.

Supplementary Table S19. Effect-measure and protocol-consistency assessment.

Protocol item	Protocol plan	Final handling	Judgement	Action / evidence
Binary effect measure	Risk ratios (RRs) were specified for binary outcomes in the protocol.	The main A-E NMA used odds ratios (ORs) from a sparse-event contrast-based random-effects netmeta framework.	Transparent analytical adaptation.	Report in Methods and Limitations; keep OR as the primary model.
Reason for OR adaptation	Use a consistent binary effect measure for connected comparative synthesis.	Rare PCP events, zero-event active arms, sparse active nodes, and model implementation favored the OR-based contrast framework.	Justified by data structure.	Certainty ratings were not driven solely by the OR/RR choice.
RR sensitivity: TMP-SMX standard vs no prophylaxis	RR robustness where feasible.	Post hoc RR sensitivity using the same arm-level data.	Directionally concordant.	RR 0.35 (95% CI 0.21–0.56).
RR sensitivity: TMP-SMX low vs no prophylaxis	RR robustness where feasible.	Post hoc RR sensitivity using the same arm-level data.	Directionally concordant.	RR 0.06 (95% CI 0.02–0.18).
RR sensitivity: TMP-SMX low vs TMP-SMX standard	RR robustness where feasible.	Post hoc RR sensitivity using the same arm-level data.	Direct active-dose evidence remained neutral.	RR 0.93 (95% CI 0.20–4.27).
RR sensitivity: pentamidine vs no prophylaxis	RR robustness where feasible.	Post hoc RR sensitivity using the same arm-level data.	Unfavorable but very-low-certainty and channeling-biased.	RR 5.79 (95% CI 2.31–14.47).
Atovaquone node	Prespecified prophylaxis regimen.	Excluded from base-case; sensitivity/narrative only.	Protocol-consistent limitation due to transitivity.	Do not rank or include in base-case estimates.
Dapsone node	Prespecified prophylaxis regimen.	Not estimable in A-E base-case.	Protocol-consistent data limitation.	State as prespecified but non-estimable; not evidence of no effect.

Note. RR sensitivity analyses were performed as post hoc robustness checks using the same arm-level data and did not replace the primary OR-based NMA model.

Supplementary Table S20. GRADE-CINeMA-informed certainty of evidence for the primary comparative effectiveness analysis.

Part A. Domain judgments.

Comparison	Within-study bias / RoB	Reporting bias	Indirectness	Imprecision	Heterogeneity	Incoherence / inconsistency	Transitivity / exchangeability	Overall certainty
TMP-SMX standard dose vs no prophylaxis / observation / placebo	Serious concern. Most contributing comparative evidence was non-randomized or older open-label evidence, with confounding by indication, historical controls, and incomplete outcome-reporting detail.	Some concern. The comparison is comparatively well represented, but small-study and selective-reporting patterns cannot be excluded.	Some concern. Populations span rheumatic/autoimmune disease and solid-organ transplantation; the clinical question remains directly relevant.	No major concern for direction; CI excludes no effect and RR sensitivity is concordant. Absolute certainty in magnitude remains limited by rare events.	No major statistical concern; direct I2 4.3%, tau2 0.050, but numerical heterogeneity is less informative in sparse-event data.	No major local signal; node-splitting p=0.193 for the primary contrast.	Some concern from variable baseline risk and observational treatment selection.	Low
TMP-SMX low dose vs no prophylaxis / observation / placebo	Serious concern. Much evidence was observational; dose selection may reflect clinician judgment, kidney function, frailty, or expected intolerance.	Some concern, possibly serious. Fewer studies and sparse events make publication or availability bias difficult to assess; formal reporting-bias assessment was not reliable owing to few studies and sparse events.	Serious concern. Low-dose studies may have lower baseline risk, different populations, and zero-event arms, limiting exchangeability with standard-dose and no-prophylaxis cohorts.	Serious concern for magnitude. Only 3 events occurred in 1689 low-dose participants in direct evidence; several active arms had zero events. Direction is more reliable than precise effect size.	Some concern; direct I2 31.3% and tau2 0.830.	No primary local incoherence signal for low dose vs no prophylaxis, but interpretation is affected by the low-vs-standard loop.	Serious concern: low-dose arms may not be clinically exchangeable with higher-risk standard-dose or no-prophylaxis arms.	Low
TMP-SMX low dose vs TMP-SMX standard dose	Serious concern. Active-dose allocation was often not randomized and may reflect tolerability-driven or renal-function-driven selection.	Some concern, possibly serious. Sparse active-comparator evidence and very few events limit assessment; formal reporting-bias assessment was not reliable owing to few studies and sparse events.	Serious concern. Indirect evidence comparing dose strategies is vulnerable to differences in baseline PCP risk and prophylaxis context.	Very serious concern. Direct evidence contained 0/467 versus 1/365 events and was statistically neutral; the network estimate is driven by indirect structure.	No major numerical heterogeneity in direct comparison (I2 0%), but this is uninformative with extremely sparse events.	Serious concern. Direct evidence was neutral whereas the network estimate favored low dose; node-splitting was borderline inconsistent (p=0.045, similar in sensitivity analyses).	Serious concern: zero-event low-dose arms and lower-risk settings may make low dose appear more favorable indirectly.	Very low

Comparison	Within-study bias / RoB	Reporting bias	Indirectness	Imprecision	Heterogeneity	Incoherence / inconsistency	Transitivity / exchangeability	Overall certainty
Pentamidine vs no prophylaxis / observation / placebo	Very serious concern. Pentamidine was often used in alternative-agent or intolerance-related contexts, with high likelihood of confounding by indication.	Serious concern. Only two direct studies; formal reporting-bias assessment is not reliable.	Very serious concern. Pentamidine recipients may not be exchangeable with no-prophylaxis cohorts because treatment choice may reflect intolerance, contraindication, or high-risk clinical status.	Serious concern. Only 5 pentamidine events among 137 participants in direct evidence; the CI is wide in relative terms despite excluding the null.	No numerical heterogeneity in the two-study direct comparison, but sparse data make this unpersuasive.	No statistical local inconsistency signal (p=0.986), but this does not resolve clinical intransitivity.	Very serious concern: strong channeling/indication bias and sparse active-node evidence.	Very low

Part B. Effect estimates and interpretation consequence.

Comparison	Evidence base and effect estimate	Overall certainty	Interpretation consequence
TMP-SMX standard dose vs no prophylaxis / observation / placebo	15 direct studies; direct OR 0.32 (95% CI 0.19-0.54); network OR 0.30 (95% CI 0.18-0.48); RR sensitivity 0.35 (95% CI 0.21-0.56).	Low	Supports a protective association of standard-dose TMP-SMX versus no prophylaxis. The conclusion should be framed as clinically important but not as high-certainty causal proof.
TMP-SMX low dose vs no prophylaxis / observation / placebo	7 direct studies; direct OR 0.05 (95% CI 0.02-0.17); network OR 0.08 (95% CI 0.03-0.18); RR sensitivity 0.06 (95% CI 0.02-0.18).	Low	Supports a low-certainty protective association versus no prophylaxis, but confidence relates mainly to direction rather than precise magnitude. Do not infer that low-dose TMP-SMX is definitively preferable to standard dose.
TMP-SMX low dose vs TMP-SMX standard dose	6 direct studies; direct OR 0.93 (95% CI 0.20-4.39); network OR 0.26 (95% CI 0.10-0.66); RR sensitivity 0.93 (95% CI 0.20-4.27).	Very low	Does not establish low-dose superiority. Phrase as uncertainty about comparative effectiveness between low- and standard-dose TMP-SMX.
Pentamidine vs no prophylaxis / observation / placebo	2 direct studies; direct OR 6.01 (95% CI 2.32-15.56); network OR 6.37 (95% CI 2.60-15.64); RR sensitivity 5.79 (95% CI 2.31-14.47).	Very low	The unfavorable estimate should not be interpreted as causal evidence that pentamidine is harmful or inferior. It should be reported as very-low-certainty, channeling-biased evidence.

Note. Certainty was rated using a GRADE-CINeMA-informed approach. Ratings refer primarily to confidence in the direction of association rather than the precise magnitude of effect, especially for sparse-event and zero-event contrasts.

Supplementary Table S21. Certainty rationale, interpretation constraints, and summary of findings.

Comparison or node	Effect estimate for PCP incidence	Certainty of evidence	Summary-of-Findings interpretation
TMP-SMX standard dose vs no prophylaxis / observation / placebo	Network OR 0.30 (95% CI 0.18-0.48); direct OR 0.32 (95% CI 0.19-0.54); RR sensitivity 0.35 (95% CI 0.21-0.56).	Low	Standard-dose TMP-SMX was associated with lower PCP incidence than no prophylaxis. Confidence in a protective association is strengthened by concordant direct, network, and RR sensitivity results, but certainty is limited by predominantly non-randomized evidence.
TMP-SMX low dose vs no prophylaxis / observation / placebo	Network OR 0.08 (95% CI 0.03-0.18); direct OR 0.05 (95% CI 0.02-0.17); RR sensitivity 0.06 (95% CI 0.02-0.18).	Low	Low-dose TMP-SMX was associated with lower PCP incidence than no prophylaxis, but the apparent magnitude should be interpreted cautiously. The low-certainty rating primarily supports the direction of association because sparse events, zero-event arms, and transitivity concerns limit confidence in the precise effect size.
TMP-SMX low dose vs TMP-SMX standard dose	Network OR 0.26 (95% CI 0.10-0.66), but direct OR 0.93 (95% CI 0.20-4.39) and RR sensitivity 0.93 (95% CI 0.20-4.27).	Very low	The evidence does not establish low-dose TMP-SMX as superior to standard-dose TMP-SMX. The network estimate favoring low dose conflicts with neutral direct evidence and borderline node-splitting inconsistency.
Pentamidine vs no prophylaxis / observation / placebo	Network OR 6.37 (95% CI 2.60-15.64); direct OR 6.01 (95% CI 2.32-15.56); RR sensitivity 5.79 (95% CI 2.31-14.47).	Very low	Pentamidine had an unfavorable estimate, but this should not be interpreted as causal harm because the evidence is sparse and likely affected by channeling/indication bias.
Atovaquone	Not included in the base-case; sensitivity/narrative node only.	Not rateable for base-case comparative effect	Available data largely reflected second-line use after TMP-SMX intolerance/discontinuation; atovaquone should not be ranked as a first-line active regimen in this evidence base.
Dapsone	No connected estimable base-case A-E comparison.	Not estimable	Dapsone was prespecified but lacked sufficient connected comparative effectiveness data. This is absence of estimable evidence, not evidence of no effect.

Note. Active-regimen ranking is not supported by this evidence base. Atovaquone was evaluated only as a sensitivity/narrative node, dapsone was prespecified but non-estimable, low-dose TMP-SMX superiority over standard dose was not established, and the pentamidine estimate should not be interpreted as causal harm or inferiority.

Section S6. Route A-S: active-regimen safety analysis

Outcome: discontinuation due to adverse events, adverse reactions, or toxicity among active TMP-SMX regimens. Comparison: low/reduced-dose or reduced-strategy TMP-SMX versus standard/conventional-dose TMP-SMX. No-prophylaxis, observation, or placebo arms were not coded as zero discontinuations because participants in those arms had no prophylactic drug to discontinue.

Supplementary Table S22. A-S base-case input and model output.

Study	Population	Design	Low/reduced events/total	Standard/conventional events/total	Study OR (95% CI)	Evidence note	Weight (%)
Ohmura 2024[49] SRD low vs conventional	rheumatic/autoimmune disease	retrospective cohort	0/60	43/126	0.016 (0.001–0.263)	Extraction note: discontinuation of SMX/TMP 0.0% in low-dose vs 34.1% in conventional-dose group.	2.9
Aggoun 2025[50] PAPTO LOCO HT	heart transplant	retrospective cohort	16/251	26/108	0.215 (0.110–0.420)	Extraction note: primary safety outcome was adverse effects leading to SXT discontinuation; 6.4% low-dose vs 24.1% standard-dose.	51.0
Takenaka 2013[19] dose escalation RD	rheumatic/autoimmune disease	retrospective cohort	0/13	5/28	0.158 (0.008–3.089)	Extraction note: retention 100% in dose-escalation group; routine group 5 patients discontinued TMP/SMX.	2.6
Okada 1999[6] CTD ST	other eligible immunocompromised	historical cohort	1/11	11/26	0.136 (0.015–1.228)	Extraction note: ST administration stopped after adverse reaction in 1/11 in 1-tablet group and 11/26 in 2-tablet group.	4.7
Utsunomiya 2017[27] optimal regimen RD	rheumatic/autoimmune disease	RCT	10/114	12/58	0.369 (0.149–0.914)	Extraction note: discontinuation due to AEs lower in HS/ES than SS; arm counts extracted as SS 12/58, HS 5/59, ES 5/55.	27.9
Yamamoto 2014[23] autoimmune RCT	rheumatic/autoimmune disease	RCT/pilot	4/17	9/18	0.308 (0.072–1.315)	Extraction note: 9/10 AEs in daily and 4/5 AEs in twice-weekly group led to discontinuation by investigator decision.	10.9

Note. Base-case active-comparator synthesis included 6 studies, 12 arms, 137 discontinuations among 830 participants. Pooled random-effects OR for low/reduced versus standard/conventional TMP-SMX was 0.234 (95% CI 0.145–0.377), $tau^2=0$, $I^2=0.0\%$. OR <1 favors low/reduced TMP-SMX for discontinuation. This is a tolerability signal only, not proof of causal safety superiority.

Supplementary Table S23. A-S sensitivity analyses and interpretation constraints.

Analysis	Studies	Low/reduced events/total	Standard/conventional events/total	OR	95% CI	tau²	I²	Interpretation
Base-case active comparator	6	31/466	106/364	0.234	0.145–0.377	0.000	0.0%	DL random-effects = fixed-effect because tau² estimated as 0; continuity correction 0.5 applied only to studies with zero cells.
Exclude heart-transplant cohort (rheumatic/autoimmune only)	5	15/215	80/256	0.226	0.098–0.520	0.173	17.8%	Direction unchanged.
RCT/pilot RCT only	2	14/131	21/76	0.350	0.162–0.757	0.000	0.0%	Only Utsunomiya 2017[27] and Yamamoto 2014; interpret cautiously.
Exclude Yamamoto 2014[23] due safety denominator caveat	5	27/449	97/346	0.216	0.114–0.408	0.098	17.3%	Direction unchanged.
Exclude older Okada 1999[6] historical cohort	5	30/455	95/338	0.237	0.131–0.426	0.077	15.8%	Direction unchanged.
Exclude studies with zero low-dose events	4	31/393	58/210	0.257	0.157–0.420	0.000	0.0%	Direction unchanged.

Note. All sensitivity analyses retained the same direction as the base-case estimate. Open-label discontinuation, non-randomized dose selection, renal-function-driven dosing, and tolerability-driven allocation may bias estimates. A-S should be interpreted as an active-comparator TMP-SMX dose/schedule tolerability analysis only.

Section S7. Route B: TMP-SMX discontinuation burden

Outcome: single-arm TMP-SMX discontinuation due to adverse events, adverse reactions, or toxicity. Route B estimates the observed discontinuation burden across eligible arms/cohorts and is not a comparative regimen-effect analysis.

Supplementary Table S24. Route B arm-level extraction and base-case input summary.

Study	Population	Clinical subgroup	Dose/strategy category	Events/total	Rate	Reported discontinuation reasons
Hudson/Tinkham 2025[52]	solid organ transplant	abdominal SOT	unclear/standard	184/930	19.8%	hyperkalemia 39%, cytopenias 35%; early discontinuation
Munthananu-chat 2024[48]	rheumatic/autoimmune	SLE	standard/renal-adjusted	10/70	14.3%	ADR leading to stop/withdrawal
Ohmura 2024[49]	rheumatic/autoimmune	systemic rheumatic disease	low/reduced	0/60	0.0%	SMX/TMP discontinuation due ADR
Ohmura 2024	rheumatic/autoimmune	systemic rheumatic disease	standard/conventional	43/126	34.1%	SMX/TMP discontinuation due ADR; 34.1%
Izuka 2021[38]	rheumatic/autoimmune	CTD; SLE subgroup available	unclear/standard	40/424	9.4%	ADR after TMP-SMX prophylaxis leading to discontinuation
Peterson 2021[39]	solid organ transplant	kidney transplant	standard/renal-adjusted	3/237	1.3%	increase in creatinine
Aggoun 2025[50]	solid organ transplant	heart transplant	low/reduced	16/251	6.4%	adverse effects leading to SXT discontinuation; cytopenia leading cause
Aggoun 2025	solid organ transplant	heart transplant	standard/conventional	26/108	24.1%	adverse effects leading to SXT discontinuation; cytopenia leading cause
Munroe 2025[53]	solid organ transplant	lung transplant	low/reduced	23/105	21.9%	SMX/TMP discontinued; leukopenia most common reason
Olsen 1993[3]	solid organ transplant	heart transplant	standard/conventional	0/19	0.0%	no discontinuation necessary
Olsen 1993	solid organ transplant	heart transplant	standard/conventional	0/20	0.0%	no discontinuation necessary
Chapman 2019[30]	solid organ transplant	kidney transplant	standard/conventional	18/580	3.1%	allergy/intolerance 11, lymphopenia 3, hyperkalemia 2, abnormal liver function 2
Urbancic 2018[29]	solid organ transplant	kidney transplant	unclear/standard	42/119	35.3%	TMP-SMX ADR/intolerance; A-type and B-type reactions
Yamanaga 2020[34]	solid organ transplant	kidney transplant	low/reduced	0/51	0.0%	no adverse events and no intolerance discontinuation

Study	Population	Clinical subgroup	Dose/strategy category	Events/total	Rate	Reported discontinuation reasons
Jinno 2022[43]	rheumatic/autoimmune	CTD on high-dose glucocorticoids	standard/conventional	102/383	26.6%	adverse events leading to discontinuation
Honda 2021[37]	rheumatic/autoimmune	rheumatic diseases on high-dose glucocorticoids	standard/conventional	70/376	18.6%	cytopenia 26, rash 23, electrolyte abnormality 10, kidney impairment 6, liver dysfunction 5
Prasad 2019[33]	solid organ transplant	kidney transplant	standard/conventional	19/438	4.3%	cessation due persistent hyperkalemia 12 and leukopenia 7
Katsuyama 2014[20]	rheumatic/autoimmune	RA receiving biologics	unclear/standard	11/82	13.4%	AE leading to switch to aerosolized pentamidine
Faraji 2022[42]	rheumatic/autoimmune	pemphigus receiving rituximab	standard/conventional	0/235	0.0%	no cotrimoxazole-related side effects observed
Havele 2021[36]	rheumatic/autoimmune	chronic inflammatory/autoimmune disease with methotrexate	standard/conventional	3/100	3.0%	TMP-SMX stopped in 3 patients due AE; 6 had combination therapy interrupted
Park 2019[32]	rheumatic/autoimmune	rheumatic disease with non-high-dose steroids	standard/conventional	9/45	20.0%	TMP-SMX withdrawn due ADR
Kitazawa 2019[31]	rheumatic/autoimmune	CTD	standard/conventional	41/96	42.7%	AE leading to discontinuation; switched to pentamidine/atovaquone
Yang 2021[40]	solid organ transplant	kidney transplant	standard/conventional	4/189	2.1%	elevated creatinine 3 and leukopenia 1; 8 total early discontinuations
Mitsides 2014[22]	solid organ transplant	kidney transplant	standard/conventional	110/290	37.9%	complications leading to stopping TMP-SMX; creatinine 63, GI 15, leukopenia 5 etc.
Takenaka 2013[19]	rheumatic/autoimmune	rheumatic disease	low/reduced	0/13	0.0%	no discontinuation in dose-escalation group
Takenaka 2013	rheumatic/autoimmune	rheumatic disease	standard/conventional	5/28	17.9%	TMP-SMX discontinued due adverse events
Torre-Cisneros 1996[5]	solid organ transplant	liver transplant	low/reduced	2/60	3.3%	leukopenia leading to discontinuation
McLaughlin 2017[26]	solid organ transplant	kidney transplant	standard/conventional	31/155	20.0%	neutropenia, hypersensitivity, hyperkalemia, pruritus, AKI, liver injury etc.
Okada 1999[6]	rheumatic/autoimmune	CTD	low/reduced	1/11	9.1%	adverse reaction/lab abnormality leading to stop
Okada 1999	rheumatic/autoimmune	CTD	standard/conventional	11/26	42.3%	adverse reaction/lab abnormality leading to stop
Utsunomiya 2017[27]	rheumatic/autoimmune	systemic rheumatic disease	standard/conventional	12/58	20.7%	discontinuation due AEs
Utsunomiya 2017	rheumatic/autoimmune	systemic rheumatic disease	low/reduced	5/59	8.5%	discontinuation due AEs
Utsunomiya 2017	rheumatic/autoimmune	systemic rheumatic disease	low/reduced	5/55	9.1%	discontinuation due AEs
Soejima 2007[10]	rheumatic/autoimmune	SLE	unclear/standard	2/54	3.7%	severe AE leading to discontinuation

Study	Population	Clinical subgroup	Dose/strategy category	Events/total	Rate	Reported discontinuation reasons
Maezawa 2012[18]	other eligible immunocompromised	CTD plus pulmonary disease patients	unclear/standard	28/539	5.2%	AE leading to discontinuation
Torre-Cisneros 1999[7]	solid organ transplant	liver transplant	low/reduced	3/60	5.0%	leukopenia leading to discontinuation in two; liver dysfunction improved after study drug discontinued in one
Higgins 1989[1]	solid organ transplant	kidney transplant	low/reduced	8/61	13.1%	suspected nephrotoxicity 5; suspected leukopenia/thrombocytopenia 3
Yamamoto 2014[23]	rheumatic/autoimmune	systemic autoimmune disease	standard/conventional	9/18	50.0%	AEs leading investigator to discontinue
Yamamoto 2014	rheumatic/autoimmune	systemic autoimmune disease	low/reduced	4/17	23.5%	AEs leading investigator to discontinue
Hiramoto 2024[47]	rheumatic/autoimmune	rheumatic and musculoskeletal diseases	unclear/standard	82/438	18.7%	ADRs associated with SMX/TMP; events improved after discontinuation
Zhu 2010[13]	solid organ transplant	kidney transplant	standard/conventional	1/10	10.0%	transient renal dysfunction, recovered after stopping SMZ-TMP

Note. The base-case Route B dataset comprised 33 independent TMP-SMX studies/cohorts, 983 discontinuations, and 6996 TMP-SMX recipients. Conference abstracts were excluded from the base case and all quantitative sensitivity analyses.

Supplementary Table S25. Overall, population-subgroup, and dose-category analyses of TMP-SMX discontinuation.

Analysis	Studies/arms, n	Events/total	Pooled proportion (95% CI)	I^2	Prediction interval	Interpretation
Overall	33 studies/cohorts	983/6996	12.3% (9.4–15.9)	93.0%	2.9%–39.9%	Primary burden analysis; conference abstracts excluded.
Population: rheumatic/autoimmune	16 studies/cohorts	465/2774	17.1% (12.9–22.3)	87.4%	6.0%–40.0%	Descriptive subgroup; not a causal comparative effect.
Population: solid organ transplant	16 studies/cohorts	490/3683	9.0% (5.5–14.3)	94.7%	1.4%–40.5%	Descriptive subgroup; not a causal comparative effect.
Population: other eligible immunocompromised	1 study/cohort	28/539	5.2% (3.6–7.4)	0.0%	Not estimable	Descriptive subgroup; not a causal comparative effect.
Dose/strategy: low/reduced	12 arms	67/803	8.5% (5.2–13.7)	67.3%	2.1%–28.3%	Descriptive subgroup; not a causal comparative effect.
Dose/strategy: standard/conventional	13 arms	133/1375	11.2% (5.6–21.2)	91.2%	1.0%–61.1%	Descriptive subgroup; not a causal comparative effect.
Dose/strategy: standard/renal-adjusted	2 arms	19/115	16.7% (10.9–24.7)	0.0%	Not estimable	Descriptive subgroup; not a causal comparative effect.
Dose/strategy: unclear/standard	13 arms	682/4265	14.3% (9.5–21.1)	96.1%	3.0%–47.1%	Descriptive subgroup; not a causal comparative effect.
Dose/strategy: unclear/mixed	1 arm	82/438	18.7% (15.3–22.7)	0.0%	Not estimable	Descriptive subgroup; not a causal comparative effect.

Note. Random-effects logit single-arm proportion meta-analysis. Overall pooled discontinuation was 12.3% (95% CI 9.4%–15.9%), with prediction interval 2.9%–39.9% and substantial heterogeneity (I^2=93.0%). Population and dose/strategy subgroup estimates are descriptive burden summaries, not causal comparisons.

Supplementary Table S26. Reported reasons for TMP-SMX discontinuation.

Reason category	Studies reporting category, n	Studies reporting the category
Cytopenia / haematological toxicity	11	Aggoun 2025[50] PAPTO LOCO HT; Higgins 1989[1] renal lowdose; Honda 2021[37] RD high GC; Hudson 2025[52] abdominal SOT; McLaughlin 2017 KT atovaquone switch; Mitsides 2014 KT TMP complications; Munroe 2025[53] lung twice weekly; Prasad 2019[33] KT dose reduction; Torre Cisneros 1996 liver lowdose; Torre Cisneros 1999 liver RCT; Yang 2021[40] KT TWDS vs DSS
Renal dysfunction / creatinine increase	7	Honda 2021 RD high GC; McLaughlin 2017 KT atovaquone switch; Mitsides 2014 KT TMP complications; Peterson 2021[39] kidney lifelong; Yamanaga 2020[34] very low dose KT; Yang 2021 KT TWDS vs DSS; Zhu 2010[13] kidney transplant
Hyperkalemia / electrolyte abnormality	5	Chapman 2019[30] renal duration; Honda 2021 RD high GC; Hudson 2025 abdominal SOT; McLaughlin 2017 KT atovaquone switch; Prasad 2019 KT dose reduction
Rash / hypersensitivity	5	Chapman 2019 renal duration; Honda 2021 RD high GC; McLaughlin 2017 KT atovaquone switch; Urbancic 2018 kidney ADR rechallenge; Yamanaga 2020 very low dose KT
Liver toxicity	4	Chapman 2019 renal duration; Honda 2021 RD high GC; McLaughlin 2017 KT atovaquone switch; Torre Cisneros 1999 liver RCT
Gastrointestinal intolerance	1	Mitsides 2014 KT TMP complications

Note. Categories were not mutually exclusive. Study-level reason reporting varied in granularity and ascertainment; counts indicate how often each category was reported among studies with extractable reason information.

Supplementary Table S27. Route B sensitivity analyses and study-selection decisions.

Panel A. Sensitivity analyses.

Scenario	k	Events/total	Pooled proportion (95% CI)	I²	Prediction interval	Interpretation
Base-case only	33	983/6996	12.3% (9.4–15.9)	93.0%	2.9%–39.9%	Primary estimate; conference abstracts were excluded from this analysis and all quantitative sensitivity analyses.
Add mixed/all-cause Zmarlicka only	34	993/7084	12.2% (9.4–15.8)	92.9%	2.9%–39.6%	Direction and magnitude remained similar.
Add age-uncertain Chantapoh only	34	992/7061	12.3% (9.5–15.9)	92.8%	2.9%–39.8%	Direction and magnitude remained similar.
Add A-S inherited Ogawa only	34	986/7045	12.1% (9.2–15.6)	92.9%	2.8%–39.5%	Direction and magnitude remained similar.
Add all non-abstract sensitivity-only candidates	36	1005/7198	12.1% (9.4–15.5)	92.5%	2.9%–39.1%	Direction and magnitude remained similar.
Replace strict with all-cause where available	33	1002/6996	12.6% (9.7–16.1)	92.8%	3.1%–39.3%	All-cause replacements were available for Chapman 2019[30], Honda 2021[37], Yang 2021[40], and Munroe 2025[53]; direction and magnitude remained similar.

Note. All Route B sensitivity analyses used eligible full-length reports; conference abstracts were not reintroduced. Replacing strict adverse-event discontinuation counts with all-cause discontinuation counts where available yielded 12.6% (95% CI 9.7%–16.1%; prediction interval 3.1%–39.3%; I^2=92.8%). Study-selection decisions are presented in Supplementary Table S27, Panel B.

Supplementary Table S27 (continued). Route B sensitivity analyses and study-selection decisions.

Panel B. Study-selection decisions.

Study	Decision	Reason
Park 2018[28]	Not included in the primary Route B analysis	The incidence of adverse drug reactions and serious adverse drug reactions was reported, but the total number of discontinuations due to adverse events was not extractable. The reported 2/262 serious discontinuations were therefore not included in the primary analysis.
He 2025[51]	Not included in Route B; included in Route C	The cohort comprised recipients of alternative prophylaxis, but the total number of patients initiating TMP-SMX in the source population was unavailable. Therefore, the 257 patients who switched prophylaxis could not be used to estimate the TMP-SMX discontinuation proportion.
Mitsides 2014[21, 22]	Potentially overlapping reports	The two reports originated from a related renal-transplant setting and may have included overlapping patients. The dapsone report contributed only to Route C, whereas the more complete TMP-SMX report was used for the Route B discontinuation analysis, avoiding duplicate use of patient data within the same analysis.
Park 2023[46]	Not included in the primary Route B analysis	Severe adverse events and overall adverse-event incidence were reported, but the number of discontinuations attributable to adverse events was not extractable.
Zmarlicka 2015[55]	Included only in sensitivity analysis	Early discontinuation included non-adverse-event or inadequately specified reasons, and reason-specific counts were unavailable; therefore, the study did not meet the strict primary outcome definition.
Chantapoh 2025[56]	Included only in sensitivity analysis	The report described the population as adults, but the eligibility criteria permitted participants aged ≥15 years and age-stratified data were unavailable.
Liu 2012[17]	Not included in the primary Route B analysis	Adverse events, including rash, liver injury, and bone-marrow suppression, were reported, but the number of TMP-SMX discontinuations attributable to adverse events was not explicitly provided.
Kim 2020 (see Supplementary Table S2)	Not included in the primary Route B analysis	The study evaluated prophylaxis duration and PCP occurrence after discontinuation; treatment discontinuations attributable to adverse events were not extractable.
Bonnet 2018 (see Supplementary Table S2)	Not included in quantitative Route B analyses	Only a conference abstract was available, with no corresponding full-length peer-reviewed report. The discontinuation denominator could not be verified.
Ogawa 2005[9]	Not included in the primary Route B analysis	Discontinuation data were available for the active-regimen safety analysis, but Route B-specific discontinuation data could not be independently confirmed from the published report. The study was therefore not included in the strict Route B analysis.
Kobayashi 2014 (see Supplementary Table S2)	Not included in quantitative Route B analyses	This conference abstract was an earlier report from a cohort subsequently described more completely by Honda 2021 [37]. It was not included separately to avoid double counting and to ensure that quantitative analyses were based on the full peer-reviewed report.

Note. Studies that did not meet the strict Route B outcome definition were either excluded from the primary analysis or examined only in sensitivity analyses, as specified above. Conference abstracts were excluded from all quantitative analyses; corresponding full-length peer-reviewed reports were used when available.

Section 8. Route C: second-line prophylaxis after TMP-SMX intolerance or discontinuation

Route C describes breakthrough PCP during second-line prophylaxis after TMP-SMX intolerance, contraindication, allergy, toxicity, or discontinuation. It is descriptive only; no network meta-analysis, regimen ranking, or equivalence claim was made.

Supplementary Table S28. Study selection and analytical handling for second-line prophylaxis.

Study	Population	Design	Decision	Reason	Key Route C data
He 2025[51]	Solid-organ transplant	Single-center retrospective cohort	Included in quantitative breakthrough analysis; safety described narratively	Alternative prophylaxis was used after sulfa allergy, hyperkalemia, leukopenia, or other TMP-SMX intolerance/contraindication; drug-specific denominators and PCP events were extractable.	Atovaquone 0/319; dapsone 0/14; no pentamidine; no PCP.
Hudson/Tinkham 2025[52]	Solid-organ transplant	Single-center retrospective cohort	Included in quantitative breakthrough analysis; drug-specific zero events inferred	The early TMP-SMX discontinuation group had clear alternative-prophylaxis denominators, and no PCP events occurred in the overall discontinuation group.	Pentamidine 0/79; dapsone 0/41; atovaquone 0/9; 55 received no prophylaxis; no PCP in the TMP-SMX discontinuation group.
Mitsides 2014[21]	Solid-organ transplant	Observational outbreak-response cohort	Included in quantitative breakthrough analysis; safety described narratively	Dapsone was used as second-line prophylaxis after documented TMP-SMX intolerance; the denominator, PCP outcome, and methemoglobinemia events were extractable.	Dapsone 0/26 PCP; methemoglobinemia 12/26; hospital admission 5/26; transfusion 2/26.
Evans 2015[24]	Solid-organ transplant	Retrospective matched cohort	Included in quantitative breakthrough analysis	Once-weekly dapsone was used in patients with contraindications or intolerance to TMP-SMX, and PCP outcomes were reported.	Dapsone 0/79 PCP at 12 months in the matched dapsone group.
Meyers 2001[8]	Solid-organ transplant	Prospective single-arm study	Included in quantitative breakthrough and tolerability analyses	Atovaquone was administered after TMP-SMX withdrawal because of drug-related adverse effects; PCP and atovaquone tolerability outcomes were extractable.	Atovaquone 0/28 PCP; 4/28 discontinued treatment because of tolerability issues.
Gabardi 2012[16]	Solid-organ transplant	Retrospective comparative cohort	Included in quantitative breakthrough and tolerability analyses	Atovaquone was used because of prior TMP-SMX intolerance or G6PD deficiency; PCP and premature discontinuation due to toxicity were reported.	Atovaquone 0/25 PCP; 0/25 discontinued prematurely because of toxicity.
Urbancic 2018[29]	Solid-organ transplant	Protocol implementation study	Narrative description only; not included quantitatively	The report described TMP-SMX adverse drug reactions and the use of dapsone or aerosolized pentamidine, but drug-specific second-line PCP and discontinuation outcomes were not extractable.	Dapsone used in four patients; use of aerosolized pentamidine decreased; no quantitative second-line outcome.

Study	Population	Design	Decision	Reason	Key Route C data
Jinno 2022[43]	Rheumatic/autoimmune disease	Retrospective comparative cohort with propensity weighting	Included in quantitative breakthrough analysis	Ninety-five patients switched from TMP-SMX to atovaquone after adverse events, while 12 received atovaquone initially; outcomes for the combined atovaquone group were extractable.	Atovaquone 5/107 PCP; no atovaquone discontinuation due to adverse events; switch-only outcomes were not separable.
Katsuyama 2014[20]	Rheumatic/autoimmune disease	Retrospective protocol-implementation cohort	Included in quantitative breakthrough analysis; denominator examined in sensitivity analysis	Aerosolized pentamidine was used either initially for contraindications or after TMP-SMX adverse events. The main denominator included both groups; the switch-only denominator was 11.	Pentamidine 0/23 in the main analysis and 0/11 in the switch-only sensitivity analysis.
Kitazawa 2019[31]	Rheumatic/autoimmune disease	Retrospective cohort	Included in quantitative breakthrough and tolerability analyses	Patients discontinuing initial TMP-SMX switched mainly to pentamidine or atovaquone; PCP and discontinuation outcomes were extractable.	Pentamidine 0/33 PCP and 5/33 discontinuations; atovaquone 0/7 PCP and 0/7 discontinuations.
Saukkonen 1996[4]	Solid-organ transplant	Retrospective cohort	Included in quantitative breakthrough and tolerability analyses	Aerosolized pentamidine was used in adult liver or kidney transplant recipients with TMP-SMX sensitivity or intolerance; PCP and toxicity outcomes were extractable.	Pentamidine 0/35 PCP; adverse events 5/35; discontinuation for severe bronchospasm 1/35.
McLaughlin 2017[26]	Solid-organ transplant	Retrospective cohort	Narrative description only; not included quantitatively	The study reported switching from TMP-SMX to atovaquone and subsequent TMP-SMX rechallenge, but atovaquone-specific PCP and discontinuation outcomes were not extractable.	Thirty-one patients switched to atovaquone; 14 were successfully rechallenged; no extractable second-line outcome.
Macesic 2016[25]	Solid-organ transplant	Retrospective cohort	Included in quantitative breakthrough and tolerability analyses	Aerosolized pentamidine was used initially or after intolerance to TMP-SMX or dapsone-pyrimethamine; PCP and treatment-limiting toxicity were extractable.	Pentamidine 0/56 PCP; adverse-event discontinuation 5/56; bronchospasm 4/56; one ICU-level reaction.
Lor 2022[57]	Solid-organ transplant	Retrospective cohort	Safety and toxicity described quantitatively; PCP outcome unavailable	Dapsone was used as alternative prophylaxis after sulfa allergy or intolerance; anemia-related discontinuation was extractable, but the PCP outcome was not reported.	Dapsone-associated anemia requiring discontinuation 8/36; hospitalization 2/36; transfusion 1/36.
Simpson 2025 (see Supplementary Table S2)	Solid-organ transplant	Conference abstract	Not included in quantitative Route C analyses	Only a conference abstract was available, with no corresponding full-length peer-reviewed report; the information was insufficient for reliable verification and risk-of-bias assessment.	Reported dapsone adverse-event discontinuation 72/187; not used quantitatively.
Bonnet 2018 (see Supplementary Table S2)	Solid-organ transplant	Conference abstract	Not included in quantitative Route C analyses	Only a conference abstract was available, with no corresponding full-length peer-reviewed report; the information was insufficient for reliable verification and risk-of-bias assessment.	Reported atovaquone 1/50 PCP; not used quantitatively.
Schumacher 2025[54]	Solid-organ transplant	Retrospective single-center cohort	Included in quantitative breakthrough analysis; safety described narratively	The full peer-reviewed report evaluated once-weekly dapsone after sulfa allergy or TMP-SMX intolerance and provided a drug-specific denominator.	Dapsone 0/175 PCP; hemoglobin, readmission, and bacteremia outcomes reported.

Note. Studies were included in quantitative Route C analyses only when drug-specific denominators and breakthrough PCP outcomes were extractable from full-length peer-reviewed reports. Potentially overlapping reports were handled conservatively to avoid double counting. Conference abstracts were excluded from all quantitative analyses and are identifiable in Supplementary Table S2.

Supplementary Table S29. Arm-level extraction dataset for breakthrough PCP during second-line prophylaxis.

Study	Drug	Population	n	Events	Crude proportion (exact 95% CI)	Reason for second-line use	Outcome note
He 2025[51]	atovaquone	solid organ transplant	319	0	0.0% (0.0–1.1)	Initial sulfa allergy or switch after TMP-SMX hyperkalemia/leukopenia/other intolerance	Drug-specific alternative prophylaxis cohort; no PCP
Hudson/ Tinkham 2025[52]	atovaquone	solid organ transplant	9	0	0.0% (0.0–33.6)	Early TMP-SMX discontinuation due hyperkalemia, cytopenias, transaminitis, AKI, hypersensitivity or intolerance	Per-agent PCP zero inferred because overall TMP-SMX discontinuation group had 0 PCP
Meyers 2001[8]	atovaquone	solid organ transplant	28	0	0.0% (0.0–12.3)	TMP-SMX withdrawn for rash, Stevens-Johnson syndrome/angioedema, marrow suppression, diarrhea or renal dysfunction	No PCP during study period or subsequent year
Gabardi 2012[16]	atovaquone	solid organ transplant	25	0	0.0% (0.0–13.7)	History of TMP-SMX intolerance or G6PD deficiency	No PCP at 12 months
Jinno 2022[43]	atovaquone	rheumatic/autoimmune	107	5	4.7% (1.5–10.6)	Mostly switch after TMP-SMX adverse events; denominator includes 95 switch + 12 initial atovaquone	Atovaquone denominator includes initial atovaquone patients; outcomes not separable for 95 switch-only
Kitazawa 2019[31]	atovaquone	rheumatic/autoimmune	7	0	0.0% (0.0–41.0)	Switch after TMP-SMX adverse events; some patients switched from pentamidine to atovaquone	No PCP in atovaquone group
He 2025[51]	dapsone	solid organ transplant	14	0	0.0% (0.0–23.2)	Initial sulfa allergy or switch after TMP-SMX intolerance	No PCP
Hudson/ Tinkham 2025[52]	dapsone	solid organ transplant	41	0	0.0% (0.0–8.6)	Early TMP-SMX discontinuation	Per-agent PCP zero inferred because overall TMP-SMX discontinuation group had 0 PCP
Mitsides 2014[21]	dapsone	solid organ transplant	26	0	0.0% (0.0–13.2)	Documented TMP-SMX intolerance	No PCP in dapsone prophylaxis patients

Study	Drug	Population	n	Events	Crude proportion (exact 95% CI)	Reason for second-line use	Outcome note
Evans 2015[24]	dapsone	solid organ transplant	79	0	0.0% (0.0–4.6)	Contraindications/intolerability to TMP-SMZ; common switch reasons AKI/leukopenia/hyperkalemia	No documented PCP at 6 or 12 months
Hudson/ Tinkham 2025[52]	pentamidine	solid organ transplant	79	0	0.0% (0.0–4.6)	Early TMP-SMX discontinuation	Per-agent PCP zero inferred because overall TMP-SMX discontinuation group had 0 PCP
Katsuyama 2014[20]	pentamidine	rheumatic/autoi mmune	23	0	0.0% (0.0–14.8)	Initial renal dysfunction/cytopenia/other contraindication or switch after TMP-SMX adverse events	Main denominator 23 includes 12 initial AP + 11 TMP-SMX AE switch; strict switch-only sensitivity 0/11
Kitazawa 2019[31]	pentamidine	rheumatic/autoi mmune	33	0	0.0% (0.0–10.6)	Switch after TMP-SMX adverse events	No PCP in pentamidine group
Saukkonen 1996[4]	pentamidine	solid organ transplant	35	0	0.0% (0.0–10.0)	Prior TMP-SMX sensitivity/intolerance or switch after TMP-SMX adverse events	No PCP
Macesic 2016[25]	pentamidine	solid organ transplant	56	0	0.0% (0.0–6.4)	Initial or switch because of TMP-SMX/dapsone-pyrimethamine intolerance/contraindication	No PCP during prophylaxis
Schumacher 2025[54]	dapsone	solid organ transplant	175	0	0.0% (0.0–1.1)	Sulfa allergy or TMP-SMX intolerance; dapsone 100 mg once weekly	Schumacher 2025 Table 2: dapsone n=175; positive PJP via PCR, 0 (0). The 2023 ACCP abstract is preliminary/duplicate and is not the source of this event count or denominator.

Note. The primary Route C analysis included 12 reports, 16 arms, 1,056 second-line prophylaxis recipients, and 5 breakthrough PCP events. Conference abstracts were excluded from the main analysis and all quantitative sensitivity analyses. Drug-specific estimates are crude exact-binomial proportions and are not comparative pooled treatment effects.

Supplementary Table S30. Breakthrough PCP summary and sensitivity analyses.

Analysis scope	Drug	Arms	Events/participants	Crude proportion (exact 95% CI)	Interpretation note
Main	Overall	16	5/1056	0.5% (0.2–1.1)	Crude exact-binomial summary; not a meta-analytic pooled effect.
Main	atovaquone	6	5/495	1.0% (0.3–2.3)	Crude exact-binomial summary; not a meta-analytic pooled effect.
Main	dapsone	5	0/335	0.0% (0.0–1.1)	Crude exact-binomial summary; not a meta-analytic pooled effect.

Analysis scope	Drug	Arms	Events/participants	Crude proportion (exact 95% CI)	Interpretation note
Main	pentamidine	5	0/226	0.0% (0.0–1.6)	Crude exact-binomial summary; not a meta-analytic pooled effect.
Sensitivity: exclude Jinno 2022[43]	Overall	15	0/949	0.0% (0.0–0.4)	Crude exact-binomial summary; not a meta-analytic pooled effect.
Sensitivity: exclude Jinno 2022	atovaquone	5	0/388	0.0% (0.0–0.9)	Crude exact-binomial summary; not a meta-analytic pooled effect.
Sensitivity: Katsuyama 2014[20] strict switch-only denominator	Overall	16	5/1044	0.5% (0.2–1.1)	Crude exact-binomial summary; not a meta-analytic pooled effect.
Sensitivity: Katsuyama 2014 strict switch-only denominator	pentamidine	5	0/214	0.0% (0.0–1.7)	Crude exact-binomial summary; not a meta-analytic pooled effect.

Note. Overall breakthrough PCP was 5/1,056 (0.5%, exact 95% CI 0.2%–1.1%). Atovaquone was 5/495 (1.0%, exact 95% CI 0.3%–2.3%); dapsone was 0/335 (upper exact 95% CI 1.1%); aerosolized pentamidine was 0/226 (upper exact 95% CI 1.6%). All-zero dapsone and pentamidine subgroups should not be interpreted as superiority.

Supplementary Table S31. Descriptive second-line safety and toxicity findings.

Panel A. Drug-level descriptive safety and toxicity summary.

Drug	Outcome	Studies, n	Events/denominator	Crude proportion (exact 95% CI)	Note
atovaquone	AE/tolerability discontinuation where extractable	4	4/167	2.4% (0.7–6.0)	Descriptive only; definitions varied and some studies did not report second-line AE discontinuation.
dapsone	AE/tolerability discontinuation where extractable	1	8/36	22.2% (10.1–39.2)	Based on the eligible full-length report; conference abstracts were excluded from all quantitative summaries and sensitivity analyses.
pentamidine	AE/tolerability discontinuation where extractable	3	11/124	8.9% (4.5–15.3)	Descriptive only; definitions varied and some studies did not report second-line AE discontinuation.
dapsone	Important dapsone toxicity (methemoglobinemia/anemia/ADE) where reported	2	20/62	32.3% (20.9–45.3)	Main descriptive summary; toxicity definitions differ across studies.

Note. These are descriptive crude proportions, not pooled comparative estimates. Conference abstracts were excluded from all quantitative safety summaries and sensitivity analyses. Definitions and denominators differed by drug and study; therefore, cross-agent safety comparisons should not be made.

Supplementary Table S31 (continued). Descriptive second-line safety and toxicity findings.

Panel B. Study-level second-line safety/toxicity notes.

Study	Drug	n	AE/tolerability discontinuations	Toxicity events	Toxicity type	Meta-usable status
He 2025[51]	atovaquone	319			Not specifically reported; access/cost barriers reported; opportunistic infections included toxoplasmosis and nocardiosis on atovaquone	No; AE discontinuation not extractable
Meyers 2001[8]	atovaquone	28	4	14	GI adverse events common; 3 diarrhea discontinuations + 1 unpleasant taste/patient request	Yes for tolerability discontinuation; includes patient request due taste
Gabardi 2012[16]	atovaquone	25	0		No premature removal owing to toxicity	Yes
Jinno 2022[43]	atovaquone	107	0		No atovaquone discontinuation due AEs	Yes
Kitazawa 2019[31]	atovaquone	7	0		No atovaquone discontinuation	Yes
Mitsides 2014[21]	dapsone	26		12	Methemoglobinemia; 5 admissions; 2 transfusions; program-level cessation of dapsone use	Toxicity yes; individual discontinuation no
Lor 2022[57]	dapsone	36	8	8	Clinically significant anemia attributed to dapsone, resolved after discontinuation; 2 hospitalized; 1 transfusion	Yes for anemia-related discontinuation
Katsuyama 2014[20]	pentamidine	23		0	No severe adverse events requiring admission after switch to AP; discontinuation not reported	No; discontinuation not extractable
Kitazawa 2019	pentamidine	33	5	5	Pentamidine discontinued; asthma most frequent reason (2 patients)	Yes
Saukkonen 1996[4]	pentamidine	35	1	5	Bronchospasm 2, dyspnea 1, cough 1, nausea 1; severe bronchospasm caused discontinuation in 1	Yes for discontinuation; any AE also reported
Macesic 2016[25]	pentamidine	56	5	5	AP-associated AE requiring discontinuation; bronchospasm 4; one ICU/epinephrine event	Yes
Schumacher 2025[54]	dapsone	175			Greater hemoglobin decrease than SMX-TMP; readmission 55.7% and bacteremia 10.8%; no dichotomous toxicity denominator extracted for pooling.	Descriptive only; not meta-usable for toxicity proportion

Note. This table supports the descriptive safety summary and should not be read as a comparative toxicity ranking.

References

1. Higgins, R.M., et al., *The risks and benefits of low-dose cotrimoxazole prophylaxis for Pneumocystis pneumonia in renal transplantation.* Transplantation, 1989. **47**(3): p. 558-560.
2. Elinder, C.G., et al., *Effectiveness of low-dose cotrimoxazole prophylaxis against Pneumocystis carinii pneumonia after renal and/or pancreas transplantation.* Transplant International : Official Journal of the European Society For Organ Transplantation, 1992. **5**(2): p. 81-84.
3. Olsen, S.L., et al., *Prevention of Pneumocystis carinii pneumonia in cardiac transplant recipients by trimethoprim sulfamethoxazole.* Transplantation, 1993. **56**(2): p. 359-362.
4. Saukkonen, K., R. Garland, and H. Koziel, *Aerosolized pentamidine as alternative primary prophylaxis against Pneumocystis carinii pneumonia in adult hepatic and renal transplant recipients.* Chest, 1996. **109**(5): p. 1250-1255.
5. Torre-Cisneros, J., et al., *Effectiveness of daily low-dose cotrimoxazole prophylaxis for Pneumocystis carinii pneumonia in liver transplantation--an open clinical trial.* Transplantation, 1996. **62**(10): p. 1519-1521.
6. Okada, J., et al., *Efficacy of sulfamethoxazole-trimethoprim administration in the prevention of Pneumocystis carinii pneumonia in patients with connective tissue disease.* Kansenshogaku Zasshi. The Journal of the Japanese Association For Infectious Diseases, 1999. **73**(11): p. 1123-1129.
7. Torre-Cisneros, J., et al., *Randomized trial of weekly sulfadoxine/pyrimethamine vs. daily low-dose trimethoprim-sulfamethoxazole for the prophylaxis of Pneumocystis carinii pneumonia after liver transplantation.* Clinical Infectious Diseases : an Official Publication of the Infectious Diseases Society of America, 1999. **29**(4): p. 771-774.
8. Meyers, B., F. Borrego, and G. Papanicolaou, *Pneumocystis carinii pneumonia prophylaxis with atovaquone in trimethoprim-sulfamethoxazole-intolerant orthotopic liver transplant patients: a preliminary study.* Liver Transplantation : Official Publication of the American Association For the Study of Liver Diseases and the International Liver Transplantation Society, 2001. **7**(8): p. 750-751.
9. Ogawa, J., et al., *Prediction of and prophylaxis against Pneumocystis pneumonia in patients with connective tissue diseases undergoing medium- or high-dose corticosteroid therapy.* Modern Rheumatology, 2005. **15**(2): p. 91-96.
10. Soejima, M., et al., *Association of the diplotype configuration at the N-acetyltransferase 2 gene with adverse events with co-trimoxazole in Japanese patients with systemic lupus erythematosus.* Arthritis Research & Therapy, 2007. **9**(2): p. R23.
11. Enomoto, T., et al., *Preventive effect of sulfamethoxasole-trimethoprim on Pneumocystis jiroveci pneumonia in patients with interstitial pneumonia.* Internal Medicine (Tokyo, Japan), 2008. **47**(1): p. 15-20.

12. Kimura, M., et al., *Comparison of trimethoprim-sulfamethoxazole and aerosolized pentamidine for primary prophylaxis of Pneumocystis jiroveci pneumonia in immunocompromised patients with connective tissue disease.* Rheumatology International, 2007. **28**(7): p. 673-676.

13. Zhu, Y.-S., et al., *Prevention of opportunistic infection after renal transplantation (in Chinese).* Organ Transplantation, 2010. **1**(4): p. 211-215. https://doi.org/10.3969/j.issn.1674-7445.2010.04.005

14. Vananuvat, P., et al., *Primary prophylaxis for Pneumocystis jirovecii pneumonia in patients with connective tissue diseases.* Seminars In Arthritis and Rheumatism, 2011. **41**(3): p. 497-502.

15. Chen, T.-Q., et al., *Prevention effect of compound sulfamethoxazole on Pneumocystis carinii pneumonia after kidney transplantation (in Chinese).* Chinese Journal of Tissue Engineering Research, 2011. **15**(5): p. 898-900. https://doi.org/10.3969/j.issn.1673-8225.2011.05.033

16. Gabardi, S., et al., *Atovaquone versus trimethoprim-sulfamethoxazole as Pneumocystis jirovecii pneumonia prophylaxis following renal transplantation.* Clinical Transplantation, 2012. **26**(3): p. E184-E190.

17. Liu, W.-J. and Zhao, M., *Small-dose trimethoprim-sulfamethoxazole prevents Pneumocystis pneumonia after renal transplantation (in Chinese).* Chinese Journal of Tissue Engineering Research, 2012. **16**(40): p. 7421-7425. https://doi.org/10.3969/j.issn.2095-4344.2012.40.003

18. Maezawa, R., et al., *Positivity for anti-RNP antibody is a risk factor for adverse effects caused by trimethoprim-sulfamethoxazole, a prophylactic agent for P. jiroveci pneumonia, in patients with connective tissue diseases.* Modern Rheumatology, 2012. **23**(1): p. 62-70.

19. Takenaka, K., et al., *A dose-escalation regimen of trimethoprim-sulfamethoxazole is tolerable for prophylaxis against Pneumocystis jiroveci pneumonia in rheumatic diseases.* Modern Rheumatology, 2012. **23**(4): p. 752-758.

20. Katsuyama, T., et al., *Prophylaxis for Pneumocystis pneumonia in patients with rheumatoid arthritis treated with biologics, based on risk factors found in a retrospective study.* Arthritis Research & Therapy, 2014. **16**(1): p. R43.

21. Mitsides, N., et al., *Dapsone-induced methemoglobinemia in renal transplant recipients: more prevalent than previously thought.* Transplant Infectious Disease : an Official Journal of the Transplantation Society, 2013. **16**(1): p. 37-43.

22. Mitsides, N., et al., *Complications and outcomes of trimethoprim-sulphamethoxazole as chemoprophylaxis for pneumocystis pneumonia in renal transplant recipients.* Nephrology (Carlton, Vic.), 2014. **19**(3): p. 157-163.

23. Yamamoto, T., et al., *A Feasibility Study Assessing Tolerability of Daily versus Twice Weekly Trimethoprim-Sulfamethoxazole Regimen for Prophylaxis against Pneumocystis Pneumonia in Patients with Systemic Autoimmune Diseases on Glucocorticoid Therapy.* Rinsho yakuri/Japanese Journal of Clinical Pharmacology and Therapeutics, 2014. **45**(3): p. 89-92.

24. Evans, R.A., et al., *Efficacy of once-weekly dapsone dosing for Pneumocystis jirovecii pneumonia prophylaxis post transplantation.* Transplant Infectious Disease :

an Official Journal of the Transplantation Society, 2015. **17**(6): p. 816-821.

25. Macesic, N., et al., *Is Aerosolized Pentamidine for Pneumocystis Pneumonia Prophylaxis in Renal Transplant Recipients Not as Safe as We Might Think?* Antimicrobial Agents and Chemotherapy, 2016. **60**(4): p. 2502-2504.
26. McLaughlin, M.M., et al., *Switch to atovaquone and subsequent re-challenge with trimethoprim-sulfamethoxazole for Pneumocystis prophylaxis in a kidney transplant population.* Transplant Infectious Disease : an Official Journal of the Transplantation Society, 2017. **19**(6).
27. Utsunomiya, M., et al., *Optimal regimens of sulfamethoxazole-trimethoprim for chemoprophylaxis of Pneumocystis pneumonia in patients with systemic rheumatic diseases: results from a non-blinded, randomized controlled trial.* Arthritis Research & Therapy, 2017. **19**(1): p. 7.
28. Park, J.W., et al., *Prophylactic effect of trimethoprim-sulfamethoxazole for pneumocystis pneumonia in patients with rheumatic diseases exposed to prolonged high-dose glucocorticoids.* Annals of the Rheumatic Diseases, 2017. **77**(5): p. 644-649.
29. Urbancic, K.F., et al., *Taking the challenge: A protocolized approach to optimize Pneumocystis pneumonia prophylaxis in renal transplant recipients.* American Journal of Transplantation : Official Journal of the American Society of Transplantation and the American Society of Transplant Surgeons, 2017. **18**(2): p. 462-466.
30. Chapman, F.A., et al., *Impact of Increased Duration of Trimethoprim-Sulfamethoxazole Prophylaxis for Pneumocystis Pneumonia After Renal Transplant.* Annals of Transplantation, 2019. **24**: p. 625-630.
31. Kitazawa, T., et al., *Efficacies of atovaquone, pentamidine, and trimethoprim/sulfamethoxazole for the prevention of Pneumocystis jirovecii pneumonia in patients with connective tissue diseases.* Journal of Infection and Chemotherapy : Official Journal of the Japan Society of Chemotherapy, 2019. **25**(5): p. 351-354.
32. Park, J.W., et al., *Pneumocystis pneumonia in patients with rheumatic diseases receiving prolonged, non-high-dose steroids-clinical implication of primary prophylaxis using trimethoprim-sulfamethoxazole.* Arthritis Research & Therapy, 2019. **21**(1): p. 207.
33. Prasad, G.V.R., et al., *Safety and efficacy of prophylaxis for Pneumocystis jirovecii pneumonia involving trimethoprim-sulfamethoxazole dose reduction in kidney transplantation.* BMC Infectious Diseases, 2019. **19**(1): p. 311.
34. Yamanaga, S., et al., *Impact of Very Low-Dose Trimethoprim-Sulfamethoxazole on Serum Creatinine after Renal Transplantation: A Retrospective Study.* Transplantation Proceedings, 2020. **52**(6): p. 1757-1761.
35. Chan, S.C.W., et al., *Epidemiology, mortality and effectiveness of prophylaxis for Pneumocystis jiroveci pneumonia among rheumatic patients: a territory-wide study.* Annals of Clinical Microbiology and Antimicrobials, 2021. **20**(1): p. 78.
36. Havele, S.A., et al., *Safety of trimethoprim-sulfamethoxazole for Pneumocystis jirovecii pneumonia prophylaxis in patients taking methotrexate.* Journal of the American Academy of Dermatology, 2020. **84**(1): p. 166-168.
37. Honda, N., et al., *Reduction of Pneumocystis jirovecii pneumonia and bloodstream infections by trimethoprim-sulfamethoxazole prophylaxis in patients with*

rheumatic diseases. Scandinavian Journal of Rheumatology, 2021. **50**(5): p. 365-371.

38. Izuka, S., et al., *Adverse drug reactions to trimethoprim-sulfamethoxazole in systemic lupus erythematosus.* Lupus, 2021. **30**(10): p. 1679-1683.

39. Peterson, K., et al., *Lifelong, universal Pneumocystis jirovecii pneumonia prophylaxis: Patient uptake and adherence after kidney transplant.* Transplant Infectious Disease : an Official Journal of the Transplantation Society, 2020. **23**(3): p. e13509.

40. Yang, H., et al., *Effectiveness and safety evaluation of thrice weekly double strength vs daily single strength trimethoprim-sulfamethoxazole for prophylaxis of pneumocystis jirovecii pneumonia after kidney transplantation: a two year prospective cohort study.* Journal of Pharmacy & Pharmaceutical Sciences : a Publication of the Canadian Society For Pharmaceutical Sciences, Societe Canadienne Des Sciences Pharmaceutiques, 2021. **24**: p. 220-226.

41. Chen, R.-Y., et al., *Prophylactic effect of low-dose trimethoprim-sulfamethoxazole for Pneumocystis jirovecii pneumonia in adult recipients of kidney transplantation: a real-world data study.* International Journal of Infectious Diseases : IJID : Official Publication of the International Society For Infectious Diseases, 2022. **125**: p. 209-215.

42. Faraji, H., et al., *Evaluating the risk-to-benefit ratio of using cotrimoxazole as a pneumocystis pneumonia preventative intervention among pemphigus patients treated with rituximab: A retrospective study with 494 patients.* Dermatologic Therapy, 2021. **35**(2): p. e15257.

43. Jinno, S., et al., *Comparative effectiveness of trimethoprim-sulfamethoxazole versus atovaquone for the prophylaxis of pneumocystis pneumonia in patients with connective tissue diseases receiving prolonged high-dose glucocorticoids.* Rheumatology International, 2021. **42**(8): p. 1403-1409.

44. Park, J.W., et al., *Primary Prophylaxis for Pneumocystis jirovecii Pneumonia in Patients Receiving Rituximab.* Chest, 2021. **161**(5): p. 1201-1210.

45. Sonomoto, K., et al., *Prophylaxis against pneumocystis pneumonia in rheumatoid arthritis patients treated with b/tsDMARDs: insights from 3787 cases in the FIRST registry.* Rheumatology (Oxford, England), 2022. **61**(5): p. 1831-1840.

46. Park, J.W., et al., *Risk-Benefit Analysis of Primary Prophylaxis Against Pneumocystis Jirovecii Pneumonia in Patients With Rheumatic Diseases Receiving Rituximab.* Arthritis & Rheumatology (Hoboken, N.J.), 2023. **75**(11): p. 2036-2044.

47. Hiramoto, K., M. Akiyama, and Y. Kaneko, *Risk factors for adverse drug reactions to sulfamethoxazole-trimethoprim prophylaxis in patients with rheumatic and musculoskeletal diseases.* Modern Rheumatology, 2024. **35**(1).

48. Munthananuchat, P., et al., *The role of trimethoprim/sulfamethoxazole in preventing opportunistic infections in systemic lupus erythematosus patients receiving low-level immunosuppressive treatment: an open-label, randomized, controlled trial.* Clinical and Experimental Medicine, 2024. **24**(1): p. 241.

49. Ohmura, S.-I., et al., *Effectiveness and safety of lower dose sulfamethoxazole/trimethoprim for Pneumocystis jirovecii pneumonia prophylaxis in patients with systemic rheumatic diseases receiving moderate-to high-dose glucocorticoids.* Respiratory Investigation, 2024. **62**(6): p. 1195-1201.

50. Aggoun, D., et al., *Prophylaxis against Pneumocystis jirovecii pneumonia and toxoplasmosis with low-dose Trimethoprim-sulfamethoxazole (cotrimoxazole 20/100*

mg) in heart transplant patients. The PAPTO-LOCO observational comparative study. The Journal of Antimicrobial Chemotherapy, 2025. **80**(5): p. 1394-1401.
51. He, K.D., et al., *Alternative Pneumocystis Pneumonia Prophylaxis in Solid Organ Transplants.* Transplant Infectious Disease : an Official Journal of the Transplantation Society, 2024. **27**(1): p. e14410.
52. Hudson, M.R., et al., *Premature Discontinuation of Trimethoprim/Sulfamethoxazole Prophylaxis in Abdominal Transplant Recipients: A Deeper Dive.* Transplant Infectious Disease : an Official Journal of the Transplantation Society, 2025. **27**(5): p. e70057.
53. Munroe, C., et al., *Twice Weekly Sulfamethoxazole/Trimethoprim for Pneumocystis jiroveci Pneumonia Prophylaxis in Lung Transplant Recipients.* Transplant Infectious Disease : an Official Journal of the Transplantation Society, 2025. **27**(5): p. e70086.
54. Schumacher, L., et al., *Safety of Once-Weekly Dapsone for Pneumocystis jirovecii Pneumonia Prophylaxis in Kidney Transplant Recipients.* Transplant Infectious Disease : an Official Journal of the Transplantation Society, 2025. **27**(3): p. e70012.
55. Zmarlicka, M., et al., *Tolerability of low-dose sulfamethoxazole/trimethoprim for Pneumocystis jirovecii pneumonia prophylaxis in kidney transplant recipients.* Progress In Transplantation (Aliso Viejo, Calif.), 2015. **25**(3): p. 210-216.
56. Chantapoh, W., et al., *Daily single-strength vs. thrice-weekly double-strength trimethoprim/sulfamethoxazole for Pneumocystis jirovecii prophylaxis in kidney transplant recipients: a non-inferiority randomized control trial.* BMC Infectious Diseases, 2025. **25**(1): p. 1372.
57. Lor, K.W., et al., *Dapsone-Associated Anemia in Heart Transplant Recipients with Normal Glucose-6-Phosphate Dehydrogenase Activity.* Journal of Clinical Medicine, 2022. **11**(21).
